# Short-term risk of falls among initiators of controlled-release tapentadol versus oxycodone in New South Wales between 2014 and 2020: A population-based retrospective cohort study

**DOI:** 10.1371/journal.pmed.1004950

**Published:** 2026-08-11

**Authors:** Ximena Camacho, Andrea L. Schaffer, Jonathan Brett, Ria Hopkins, Natasa Gisev, Steven Marsh, Kristian B. Filion, Nicole Pratt, David Henry, Sallie-Anne Pearson

**Affiliations:** 1 Centre of Research Excellence in Medicines Intelligence, Sydney, New South Wales, Australia; 2 School of Population Health, Faculty of Medicine and Health, UNSW Sydney, Sydney, New South Wales, Australia; 3 The Bennett Institute for Applied Data Science, Nuffield Department of Primary Care Health Sciences, University of Oxford, Oxford, United Kingdom; 4 Clinical Pharmacology and Toxicology, St Vincent’s Hospital, Sydney, New South Wales, Australia; 5 National Drug and Alcohol Research Centre, UNSW Sydney, Sydney, New South Wales, Australia; 6 Centre for Clinical Epidemiology, Lady Davis Institute for Medical Research, Jewish General Hospital, Montreal, Québec, Canada; 7 Department of Epidemiology, Biostatistics and Occupational Health, McGill University, Montreal, Québec, Canada; 8 Department of Medicine, McGill University, Montreal, Québec, Canada; 9 Quality Use of Medicines and Pharmacy Research Centre, School of Pharmacy and Biomedical Science, College of Health, Adelaide University, Adelaide, South Australia, Australia; 10 Institute for Evidence Based Healthcare, Bond University, Gold Coast, Queensland, Australia; University of melbourne, AUSTRALIA

## Abstract

**Background:**

Clinical trials comparing sustained-release (SR) tapentadol with controlled-release (CR) oxycodone have suggested that tapentadol (SR) may be associated with fewer nervous system side effects. However, it remains important to evaluate the relative safety of these agents in real-world clinical settings. Given the potential severity and economic burden of falls, together with their established association with opioid use, we compared the short-term risk of falls following initiation of tapentadol (SR) versus oxycodone (CR) in routine clinical practice.

**Methods and findings:**

We conducted an active comparator, new user retrospective cohort study using linked routinely collected health data on residents of New South Wales, Australia (2014–2020). We included people aged ≥18 years initiating publicly subsidised tapentadol (SR) or oxycodone (CR). Our outcome was a composite measure of fall-related emergency department presentations, hospitalisations or deaths. We used propensity score matching to adjust for baseline confounding and approximated relative risks (RR) of falls at 7, 14, and 28 days after initiation using conditional logistic regression models. We calculated absolute risk differences and estimated the number of people that would need to be treated with oxycodone (CR) versus tapentadol (SR) for one additional fall to occur (NNTH). We conducted subgroup analyses restricted to people aged ≥65 and ≥80 years and by recent opioid exposure (within 90 days prior to initiation).

We identified 103,924 tapentadol (SR) and 419,732 oxycodone (CR) initiators; after matching each cohort comprised 103,758 initiators. Most people (74%) were aged between 45 and 84 years, and slightly more than half were female. Within 28 days of initiation, 652 (0.6%) oxycodone (CR) initiators and 457 (0.4%) tapentadol initiators experienced a fall. Across all time points, tapentadol (SR) initiation was associated with a lower risk of falls compared with oxycodone (CR) (7 days: RR 0.57 [95% CI: 0.48, 0.69]; 14 days: RR 0.62 [95% CI: 0.54, 0.71]; 28 days: RR 0.70 [95% CI: 0.62, 0.79]). Absolute differences were small at all time points (approximately 1–2 fewer falls per 1,000 patients treated), corresponding to NNTH for one additional fall ranging from 739 to 529. These patterns persisted regardless of recent opioid exposure. Relative risks were similar in the older age groups while absolute differences were slightly larger (≥65 years: 2–3 fewer falls/1,000; ≥80 years: 4–8 fewer falls/1,000). The greatest absolute differences were among opioid-naïve people aged ≥80 years (6–10 fewer falls/1,000, corresponding to NNTH ranging from 173 to 97). Fall risks were attenuated among people aged ≥80 years with recent opioid exposure. The main limitations of this study were that we did not have data on immediate-release tapentadol, or on falls that did not result in emergency department presentations or hospitalisations but may have otherwise impacted independence and mobility.

**Conclusions:**

Tapentadol (SR) was associated with a lower risk of falls than oxycodone (CR) up to four weeks after initiation, although absolute differences were small. The reduction in risk may be an important consideration in older patients where the consequences of falls are most severe.

## Author summary

**Why was this study done?:**

- Falls can have catastrophic consequences for people and substantial economic impacts on health systems.
- Opioids are associated with increased risks of falls, particularly in the first 28 days of use.
- Clinical trials comparing two similar opioids, sustained-release (SR) tapentadol and controlled-release (CR) oxycodone, have suggested that tapentadol has fewer side effects, but it is unclear whether people starting tapentadol (SR) in real-world settings experience fewer falls than people starting oxycodone (CR).

**What did the researchers do and find?:**

- We compared the rates of falls that resulted in either a hospital admission, emergency department visit, or death (which we termed “falls”) among 207,516 people who started tapentadol (SR) versus oxycodone (CR). These people could also have been prescribed other opioids at the same time.
- We further examined falls among older adults and among people who were being prescribed opioids for the first time.
- We found that people starting tapentadol (SR) were less likely to fall in the first 28 days of use compared to people starting oxycodone (CR), but these differences were small (approximately 1–2 fewer falls per 1,000 patients treated).
- People starting tapentadol (SR) who were aged 80 years and older and who were being prescribed opioids for the first time were the least likely to fall (approximately 6–10 fewer falls per 1,000 patients treated).

**What do these findings mean?:**

- Even though the difference in fall risk between tapentadol (SR) and oxycodone (CR) was small, given the widespread use of opioids this could translate to a substantial reduction in falls and their associated impacts on well-being and costs.
- The reduction in risk may be important to consider when making opioid prescribing decisions, especially among older people where the consequences of falls are most severe.
- The main limitations of this study were the lack of data on immediate-release tapentadol and on all falls (not just falls that required hospital or emergency care or resulted in death).

## Abstract 3

In a population-based retrospective cohort study, Ximena Camacho and colleagues investigate the short-term risk of falls resulting in emergency department presentations, hospital admissions, or death, following initiation of tapentadol compared to initiation of oxycodone in New South Wales, Australia from 2014 to 2020.

## Introduction

Tapentadol is a µ-opioid agonist, moderate noradrenaline reuptake inhibitor (NRI) and weak serotonin reuptake inhibitor. Real-world safety of its sustained-release (SR) formulation is not yet fully understood [1,2]. Central nervous system disorders, including dizziness and somnolence, were among the most common side effects (occurring in ≥1 in 10 people) reported in all tapentadol (SR) clinical trials [3]. Nonetheless, clinical trials of tapentadol (SR) versus controlled-release (CR) oxycodone, morphine CR, and oxycodone/naloxone combination products suggest that tapentadol (SR) has better tolerability [4] and a lower incidence of serious adverse events than the comparator opioids [4,5]. This may be due to pharmacological differences (e.g., comparatively lower opioid potency or NRI effect with tapentadol (SR)) [4]. Both phase III and long-term safety trials have identified lower rates of dizziness and somnolence in tapentadol (SR) treatment arms versus oxycodone (CR), respectively [3,6,7].

However, populations studied in clinical trials do not necessarily reflect people treated with these medicines in real-world settings. Specifically, trial participants were not permitted to use other non-study opioids. This situation does not reflect real-world clinical practice, where multiple opioids including immediate-release formulations are often used to manage pain [8]. Moreover, the trials were designed to assess efficacy in reducing pain and underpowered to assess safety outcomes. In light of clinical trial evidence demonstrating that tapentadol (SR) and oxycodone (CR) achieve comparable analgesic effects and improvements in quality of life [4,9], it is relevant to compare the safety of these products in real-world settings.

Opioids are associated with increased risks of falls compared to non-use [10,11]; this risk is highest in the first 7 days following initiation [12,13], in older people [11], and persists through the first 28 days of use [12,14]. Falls can have devastating consequences, including mortality and life-long impacts on independence and mobility, particularly among older people [14]. Given the known prevalence of central nervous system side effects observed in tapentadol (SR) trials and the established link between opioid use and falls, we aimed to quantify the short-term risk of falls resulting in emergency department (ED) presentations, hospital admissions, or death, following initiation of tapentadol (SR) compared to initiation of oxycodone (CR) in real-world settings that included people who concurrently used multiple opioids. Based on prior clinical trial evidence, we hypothesised that tapentadol (SR) would be associated with a lower incidence of falls than oxycodone (CR).

## Methods

We conducted an active comparator, new user retrospective cohort study to assess the risk of falls in individuals aged ≥18 years, in a real-world population that includes people who use other opioids alongside the opioids of interest. This study is reported in accordance with the Reporting of studies Conducted using Observational Routinely-collected health Data (RECORD) statement [15] (S1 Checklist). The study protocol is provided in S1 Protocol.

### Patient and public involvement

One of the co-investigators of this study (SM) is a consumer with lived experience being prescribed opioids (including tapentadol (SR)) to manage chronic pain. He has collaborated with us since study conception and was instrumental in helping refine the study question to focus on outcomes of tapentadol (SR) and oxycodone (CR) in a real-world context, including the inclusion of people who are prescribed multiple opioids to manage pain. He has provided input on the interpretation of findings to ensure they resonate with medicines end-users and will be involved in creating materials to disseminate study findings to patient groups and the wider community.

### Setting and data source

Australia has a universal healthcare system (Medicare) providing subsidised access to health services, including prescription medicines, and hospital care to all Australian citizens, permanent residents, and citizens of countries with reciprocal arrangements (e.g., New Zealand, United Kingdom, Ireland, and some European countries). The Pharmaceutical Benefits Scheme (PBS) is a national program that provides access to subsidised prescription medicines for all Medicare-eligible people. This study used the Medicines Intelligence (MedIntel) Data Platform [16]. Briefly, the platform comprises longitudinal, linked population-level data for all adult Medicare-eligible residents of New South Wales (NSW), Australia’s most populous state (8.5 million), between 2005 and 2020. The platform includes data on presentations to EDs and admissions to hospitals in NSW, cancer notifications, dispensing records for publicly subsidised medicines, and fact and cause of death information. More details on individual datasets are provided in S1 Table.

### Study population and study opioids

The study population comprised all adults (aged 18 years and over on the date of initiation) who initiated either tapentadol (SR) or oxycodone (CR) between 1 September 2014 and 3 December 2020.

The exposure of interest was publicly subsidised tapentadol (SR). Tapentadol prescribing (all formulations) has increased in Australia [17], and emerging evidence suggests tapentadol is replacing oxycodone as the preferred analgesic for post-surgical pain management [18]. Although both immediate-release and sustained-release formulations are approved for use in Australia, only tapentadol (SR) is publicly subsidised. Consequently, most tapentadol sold in Australia is sustained-release, and most tapentadol (SR) sold is publicly subsidised [17,19]. Immediate-release tapentadol is only accessible on the private market, and individual-level data on privately prescribed use were not available. Private sales of all prescribed opioids account for 27% of the overall opioid market in Australia (in oral morphine equivalent milligrams [OME]) [20].

We selected oxycodone (CR) as our comparator to align with pre-registration clinical trials of tapentadol (SR). Oxycodone (CR) was the comparator most often used in trials [4] and most aligns with the severity and nature of pain treated with tapentadol (SR). Both tapentadol (SR) and oxycodone (CR) are subsidised by the PBS (Australia’s public medicines subsidy program) and have identical listed indications for use in patients with chronic severe pain requiring daily, continuous, long-term opioid treatment [21–23]. The comparator group included both single oxycodone (CR) and combination oxycodone (CR) + naloxone products (see S2 Table for definitions).

Tapentadol (SR) was first listed on the PBS in June 2014. We therefore considered 1 June–31 August 2014 as a washout period to allow for switching from private dispensing. We excluded anyone with a publicly subsidised dispensing for tapentadol (SR) during this period.

We defined initiation as a dispensing during the accrual period with no prior dispensings for either tapentadol (SR) or oxycodone (CR) in the previous 365 days. We defined the index date as the first date of initiation with either tapentadol (SR) or oxycodone (CR) during the accrual period (S1 Fig). For people who met the inclusion criteria more than once, we only included the first initiation.

We excluded people who: died or were non-residents of NSW on the day of initiation; lived in NSW for less than 1 year prior to initiation (in order to ensure sufficient data to ascertain comorbidities and history of falls); had no dispensing history in the two years prior to cohort entry; initiated both tapentadol (SR) and oxycodone (CR) on the same day; had a fall resulting in hospitalisation or ED visit in the 60 days prior to and including initiation (to minimise the risk of protopathic bias). A graphical presentation of the study design is presented in S1 Fig. The PBS is a national program and people retain eligibility when moving between Australian states and territories; hence, the lookback period for dispensing history exceeds the requirement for residence in NSW.

### Exposure and follow-up

We considered three different follow-up periods to ascertain outcomes: 7, 14, and 28 days following initiation. We chose the maximum 28-day period as it is known to be the highest risk period for falls following opioid initiation [12–14]. We followed each person from initiation until the first of: outcome of interest (fall), end of follow-up (7, 14, or 28 days), non-fall-related hospital admission (occurred in ≤0.1% of the cohort), death, or study end date (31 December 2020). We defined exposure using an intention-to-treat approach, where we assumed people were exposed to the initiated opioid (tapentadol (SR) or oxycodone (CR)) for the duration of follow-up [24].

### Outcomes

We defined a composite measure of falls resulting in hospitalisation, ED presentation, or death (hereafter referred to as “falls”) occurring during follow-up. We ascertained falls from the hospital data using the International Classification of Diseases (ICD), 10th edition, Australian Modification (ICD-10-AM). We classified a diagnosis code representing a fall recorded in any data field (i.e., either principal or additional diagnosis) as an outcome. We excluded falls that had a flag indicating they occurred in a hospital setting. From ED records, we identified falls where a fall-related code was recorded as the principal diagnosis using ICD-10-AM, ICD-9, and Systematized Nomenclature of Medicine-Clinical Terms-Australian version (SNOMED-CT-AU) codes; and from death records where a fall-related ICD-10 code was recorded as either the underlying or contributing cause of death [14] (S3 Table).

### Covariates

We included covariates that could potentially confound the association between opioids and falls, based on the literature and clinical input. We ascertained these using hospital diagnoses and dispensing claims (S4 Table). For each person, we obtained information on their demographic characteristics at the time of opioid initiation (age, sex, relative social disadvantage [25], remoteness of residence [26]), comorbidities [27–31] (cancer, history of illicit drug use, any prior overdose, depression, anxiety, hypertension, atrial fibrillation or flutter, diabetes, heart failure, thyroid disease, postural hypotension, osteoporosis, stroke, renal impairment, hepatic impairment), and frailty (defined using the Hospital Frailty Risk Score [32,33]). We also identified recent surgeries [34,35] (30 days prior to initiation) and history of falls (assessed in the 2 years to 61 days prior to initiation).

We assessed exposure to non-study opioids (i.e., dispensing of any opioid other than tapentadol (SR) or oxycodone (CR)) in the 90 days prior to initiation and defined people without prior exposure as “opioid-naïve”. We also examined exposure to medicines with known risks of falls [10,36,37] (falls-risk medicines; FRIDs), gabapentinoids, anticholinergic medicines [38], and statins [39–41] in the 30 days prior to initiation. Finally, we quantified initiation of non-study opioids on the same day as tapentadol (SR)/oxycodone (CR) initiation; initiation was defined as a dispensing for a non-study opioid with no other dispensings for that opioid in the prior year.

### Statistical analysis

We summarised the characteristics of tapentadol (SR) and oxycodone (CR) initiators using descriptive statistics and assessed group balance using standardised differences, with values >0.1 indicating meaningful imbalance [42].

We used propensity scores to adjust for baseline confounding. We defined a propensity score model using logistic regression with the study opioid as the dependent variable and all the covariates defined above (except for initiation of non-study opioids) as independent predictors. We matched each tapentadol (SR) initiator to an oxycodone (CR) initiator (1:1) based on month and year of initiation, exposure to non-study opioids in the previous 90 days, and propensity score (using a standard calliper width of 0.2 * standard deviation of the logit of the propensity score [43]). We chose to match on propensity score (rather than weight or stratify) to carefully account for underlying trends in opioid dispensing (e.g., increasing dispensing of tapentadol over time, decreasing use of other publicly subsidised opioids, and changes to opioid prescribing policies [including codeine being moved to a prescription-only medicine]) [19] and the impact of prior opioid exposure.

#### Main analyses

We calculated separately the unadjusted relative risk (RR) under the exposure (tapentadol) and comparator (oxycodone) treatments for each outcome at 7, 14, and 28 days following initiation. We used conditional logistic regression models to approximate relative risks in the matched sample, with robust standard errors to account for the matched data. We also calculated an adjusted estimate whereby the model included a term for simultaneous initiation of study and non-study opioids. We estimated the risk differences for falls on tapentadol (SR) and oxycodone (CR) from the product of the absolute risks in the oxycodone (control) cohorts and the adjusted RR value for the relevant cohort. We estimated the number of people that would need to be treated with oxycodone (CR), rather than tapentadol (SR), for one additional fall to occur (number-needed-to-treat-to-harm; NNTH), from the inverse of the risk difference [44,45].

#### Subgroup analyses

As risks of falls and related morbidity increase with age [14], we created separate cohorts comprising people aged 65 years and over (65+), and people aged 80 years and older (80+) at the time of opioid initiation. We conducted our main analyses in each of these cohorts.

To assess potential confounding from recent exposure to non-study opioids, we conducted stratified analyses examining the risk of falls among people with and without non-study opioid exposure in the 90 days before treatment initiation. We conducted these analyses among our main study population and older age cohorts (65+ years, 80+ years).

#### Sensitivity analyses

We conducted several sensitivity analyses to assess potential confounding or effect modification of concurrent use of non-study opioids on the risk of falls. To try to isolate the effect of the study opioids, we first restricted our study population to people who were dispensed only tapentadol (SR) or oxycodone (CR) at initiation (i.e., had no dispensings of any non-study opioids on that date). We then further restricted that cohort to people without exposure to non-study opioids in the previous 90 days.

Given that both sustained- and immediate-release opioids are often used together in practice [8], we were interested in assessing the risk of falls in people simultaneously initiating both the study opioid and an immediate-release formulation. We restricted our study population to people who initiated both oxycodone IR (the most commonly used immediate-release opioid in Australia [20]) and study opioids on the same day.

We used a Kaplan–Meier approach to assess whether the time to falls differed between the matched tapentadol (SR) and oxycodone (CR) groups and plotted cumulative incidence curves.

We calculated the E-value [46] to evaluate the robustness of our main findings against unmeasured confounding.

To assess the magnitude of any residual confounding we included cataract surgery as a negative control outcome. We ascertained cataract surgeries occurring during follow-up from the hospital records using Australian Classification of Health Interventions (ACHI) procedure codes (S3 Table).

Finally, as our primary outcome is a composite, we calculated event rates and relative risks of falls separately for each constituent component (ED presentation, hospital admission, and death).

All analyses were conducted with SAS v9.4 (SAS Institute, Cary NC). This project received ethical approval from the New South Wales Population and Health Services Research Ethics Committee (PHSREC; approval number 2020/ETH02273) and the Australian Institute of Health and Welfare Human Research Ethics Committee (AIHW HREC; approval number EO2021/1/1233). This study was approved via a waiver of informed consent as the data were anonymised.

## Results

**Fig 1:**
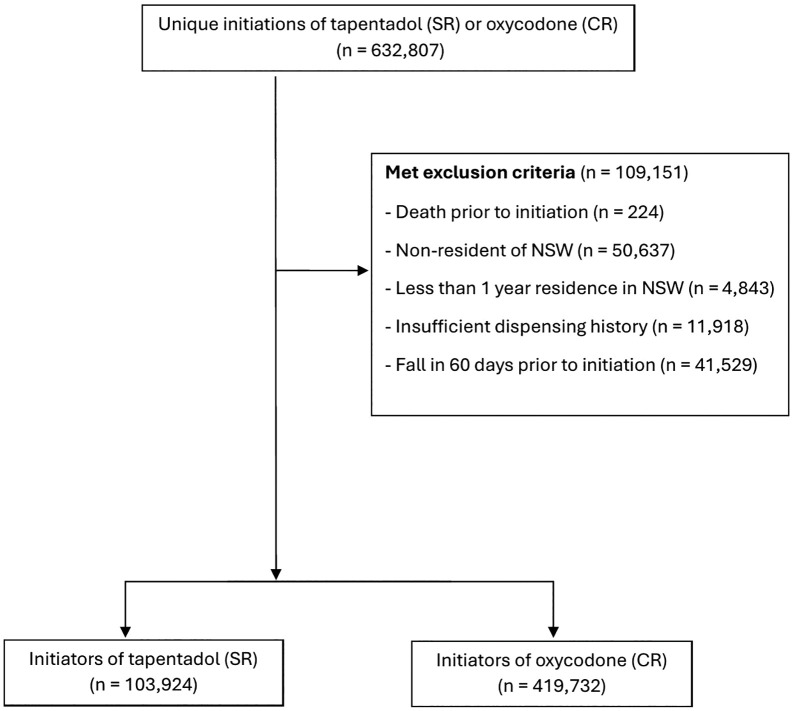
Cohort flow diagram for tapentadol (SR) and oxycodone (CR) initiators, 1 September 2014–3 December 2020. SR, sustained release; CR, controlled release; NSW, New South Wales.

We identified 103,924 tapentadol (SR) and 419,732 oxycodone (CR) initiators between 1 September 2014 and 3 December 2020; nearly three quarters were aged between 45 and 84 years, and slightly more than half were female. Approximately 40% of initiators of both study drugs had been dispensed non-study opioids in the prior 90 days (Table 1; Fig 1). We matched 103,758 tapentadol (SR) initiators to 103,758 oxycodone (CR) initiators on propensity scores, year and month of initiation, and exposure to non-study opioids in the prior 90 days (99% match rate).

**Table 1:** Baseline characteristics of tapentadol (SR) and oxycodone (CR) initiators, 1 September 2014–3 December 2020, before and after propensity score matching.

Characteristic	Unmatched			Matched		
	Tapentadol SR	Oxycodone CR	SD	Tapentadol SR	Oxycodone CR	SD
	*N* = 103,924	*N* = 419,732		*N* = 103,758	*N* = 103,758	
Year of initiation*						
2014	2,911 (2.8%)	26,035 (6.2%)	.16	2,910 (2.8%)	2,910 (2.8%)	.00
2015	9,746 (9.4%)	79,182 (18.9%)	.27	9,740 (9.4%)	9,740 (9.4%)	.00
2016	11,399 (11.0%)	83,189 (19.8%)	.25	11,394 (11.0%)	11,394 (11.0%)	.00
2017	17,232 (16.6%)	81,052 (19.3%)	.07	17,220 (16.6%)	17,220 (16.6%)	.00
2018	19,726 (19.0%)	65,941 (15.7%)	.09	19,712 (19.0%)	19,712 (19.0%)	.00
2019	24,331 (23.4%)	52,067 (12.4%)	.29	24,308 (23.4%)	24,308 (23.4%)	.00
2020	18,579 (17.9%)	32,266 (7.7%)	.31	18,474 (17.8%)	18,474 (17.8%)	.00
Sex						
Female	59,588 (57.3%)	223,611 (53.3%)	.08	59,455 (57.3%)	59,482 (57.3%)	.00
Male	44,336 (42.7%)	196,121 (46.7%)		44,303 (42.7%)	44,276 (42.7%)	
Age group (years)						
18–44	21,027 (20.2%)	85,667 (20.4%)	.00	21,023 (20.3%)	21,238 (20.5%)	.01
45–64	36,656 (35.3%)	134,311 (32.0%)	.07	36,591 (35.3%)	36,417 (35.1%)	.00
65–84	40,395 (38.9%)	163,146 (38.9%)	.00	40,317 (38.9%)	39,956 (38.5%)	.01
85+	5,846 (5.6%)	36,608 (8.7%)	.12	5,827 (5.6%)	6,147 (5.9%)	.01
Remoteness area						
Major cities	68,288 (65.7%)	273,008 (65.0%)	.01	68,187 (65.7%)	68,593 (66.1%)	.01
Inner regional	27,917 (26.9%)	109,882 (26.2%)	.02	27,857 (26.9%)	27,650 (26.7%)	.00
Outer regional	6,787 (6.5%)	32,307 (7.7%)	.05	6,783 (6.5%)	6,580 (6.3%)	.01
Remote	436 (0.4%)	2,380 (0.6%)	.02	435 (0.4%)	464 (0.5%)	.00
Very remote	59 (0.1%)	270 (0.1%)	.00	59 (0.1%)	56 (0.1%)	.00
Missing	437 (0.4%)	1,885 (0.5%)	.00	437 (0.4%)	415 (0.4%)	.00
Decile of relative disadvantage						
1 (most disadvantage)	7,529 (7.2%)	31,519 (7.5%)	.01	7,518 (7.3%)	7,437 (7.2%)	.00
2	9,496 (9.1%)	38,327 (9.1%)	.00	9,480 (9.1%)	9,410 (9.1%)	.00
3	8,349 (8.0%)	34,966 (8.3%)	.01	8,338 (8.0%)	8,106 (7.8%)	.01
4	12,276 (11.8%)	52,375 (12.5%)	.02	12,264 (11.8%)	12,152 (11.7%)	.00
5	13,443 (12.9%)	47,019 (11.2%)	.05	13,386 (12.9%)	13,418 (12.9%)	.00
6	11,808 (11.4%)	48,230 (11.5%)	.00	11,791 (11.4%)	11,895 (11.5%)	.00
7	10,448 (10.1%)	42,044 (10.0%)	.00	10,434 (10.1%)	10,584 (10.2%)	.00
8	9,262 (8.9%)	38,540 (9.2%)	.01	9,256 (8.9%)	9,335 (9.0%)	.00
9	11,762 (11.3%)	46,468 (11.1%)	.01	11,748 (11.3%)	11,752 (11.3%)	.00
10 (least disadvantage)	9,087 (8.7%)	38,298 (9.1%)	.01	9,079 (8.8%)	9,236 (8.9%)	.01
Missing	464 (0.5%)	1,946 (0.5%)	.00	464 (0.5%)	433 (0.4%)	.00
Exposure to non-study opioids (prior 90 days)						
None	65,715 (63.2%)	250,030 (59.6%)	.08	65,592 (63.2%)	65,592 (63.2%)	.00
Yes	38,209 (36.8%)	169,702 (40.4%)		38,166 (36.8%)	38,166 (36.8%)	
Comorbidities						
Cancer	8,891 (8.6%)	61,314 (14.6%)	.19	8,873 (8.6%)	9,026 (8.7%)	.01
Substance use disorder	4,729 (4.6%)	19,648 (4.7%)	.01	4,718 (4.6%)	4,617 (4.5%)	.00
Prior overdose	243 (0.2%)	984 (0.2%)	.00	242 (0.2%)	224 (0.2%)	.00
Depression	35,804 (34.5%)	128,261 (30.6%)	.08	35,692 (34.4%)	35,677 (34.4%)	.00
Anxiety	14,149 (13.6%)	53,353 (12.7%)	.03	14,112 (13.6%)	13,728 (13.2%)	.01
Hypertension	21,741 (20.9%)	92,326 (22.0%)	.03	21,655 (20.9%)	22,884 (22.1%)	.03
Atrial fibrillation or flutter	2,118 (2.0%)	12,539 (3.0%)	.06	2,115 (2.0%)	2,100 (2.0%)	.00
Diabetes	16,223 (15.6%)	69,008 (16.4%)	.02	16,193 (15.6%)	15,920 (15.3%)	.01
Congestive heart failure	14,253 (13.7%)	65,586 (15.6%)	.05	14,215 (13.7%)	14,221 (13.7%)	.00
Thyroid disease	8,998 (8.7%)	34,471 (8.2%)	.02	8,969 (8.6%)	9,149 (8.8%)	.01
Postural hypotension	516 (0.5%)	2,751 (0.7%)	.02	512 (0.5%)	519 (0.5%)	.00
Osteoporosis	12,923 (12.4%)	65,530 (15.6%)	.09	12,890 (12.4%)	13,057 (12.6%)	.00
Stroke	22,140 (21.3%)	105,878 (25.2%)	.09	22,119 (21.3%)	21,014 (20.3%)	.03
Renal impairment	1,625 (1.6%)	10,326 (2.5%)	.06	1,619 (1.6%)	1,560 (1.5%)	.00
Hepatic impairment	1,007 (1.0%)	7,010 (1.7%)	.06	1,006 (1.0%)	875 (0.8%)	.01
Surgery (prior 30 days)	20,835 (20.1%)	108,991 (26.0%)	.14	20,818 (20.1%)	20,953 (20.2%)	.00
Falls-risk medicines (FRIDs; prior 30 days)						
Yes	52,655 (50.7%)	208,948 (49.8%)	.02	52,528 (50.6%)	51,948 (50.1%)	.01
Number of FRIDs:						
1	30,347 (57.6%)	116,957 (56.0%)	.03	30,292 (57.7%)	29,195 (56.2%)	.03
2	14,772 (28.1%)	59,630 (28.5%)	.01	14,728 (28.0%)	14,829 (28.6%)	.01
3	5,509 (10.5%)	23,212 (11.1%)	.02	5,489 (10.5%)	5,749 (11.1%)	.02
4	1,552 (3.0%)	7,023 (3.4%)	.02	1,545 (2.9%)	1,682 (3.2%)	.02
5+	475 (0.9%)	2,126 (1.0%)	.01	474 (0.9%)	493 (1.0%)	.00
Type of FRID						
*Medicines associated with high risk of falls*						
Antipsychotics (excluding lithium)	1,922 (3.7%)	8,841 (4.2%)	.03	1,916 (3.7%)	2,170 (4.2%)	.03
Anxiolytics	4,298 (8.2%)	17,562 (8.4%)	.01	4,288 (8.2%)	4,487 (8.6%)	.02
Hypnotics and sedatives	2,803 (5.3%)	13,367 (6.4%)	.05	2,797 (5.3%)	2,866 (5.5%)	.01
Antidepressants	20,282 (38.5%)	74,326 (35.6%)	.06	20,211 (38.5%)	21,146 (40.7%)	.05
*Medicines that cause orthostatism/hypotension*						
Vasodilators used in cardiac disease (e.g., nitrates)	1,522 (2.9%)	8,153 (3.9%)	.06	1,520 (2.9%)	1,604 (3.1%)	.01
Antihypertensives	2,116 (4.0%)	7,867 (3.8%)	.01	2,104 (4.0%)	2,013 (3.9%)	.01
Diuretics	3,415 (6.5%)	17,478 (8.4%)	.07	3,406 (6.5%)	3,594 (6.9%)	.02
Beta blockers	7,875 (15.0%)	34,166 (16.4%)	.04	7,858 (15.0%)	7,856 (15.1%)	.00
Calcium channel blockers	8,262 (15.7%)	35,483 (17.0%)	.03	8,239 (15.7%)	8,339 (16.1%)	.01
Renin-angiotensin acting agents	30,161 (57.3%)	118,397 (56.7%)	.01	30,086 (57.3%)	29,065 (56.0%)	.03
Alpha adrenoreceptor blockers	1,168 (2.2%)	4,741 (2.3%)	.00	1,166 (2.2%)	1,114 (2.1%)	.01
Dopaminergic agents	1,278 (2.4%)	4,638 (2.2%)	.01	1,275 (2.4%)	1,140 (2.2%)	.02
Other medicines associated with falls (prior 30 days)						
Gabapentinoids	12,226 (11.8%)	34,542 (8.2%)	.12	12,113 (11.7%)	11,844 (11.4%)	.01
Anticholinergic medicines	33 (0.0%)	177 (0.0%)	.01	33 (0.0%)	30 (0.0%)	.00
Statins	26,591 (25.6%)	103,916 (24.8%)	.02	26,513 (25.6%)	26,369 (25.4%)	.00
Hospital frailty risk score						
Low: < 5	91,984 (88.5%)	351,919 (83.8%)	.14	91,838 (88.5%)	91,944 (88.6%)	.00
Medium: 5–15	8,961 (8.6%)	48,387 (11.5%)	.10	8,954 (8.6%)	8,751 (8.4%)	.01
High: > 15	2,979 (2.9%)	19,426 (4.6%)	.09	2,966 (2.9%)	3,063 (3.0%)	.01
History of falls						
Falls in the past 2 years to 60 days	3,884 (3.7%)	21,089 (5.0%)	.06	3,871 (3.7%)	3,971 (3.8%)	.01

Note: cohort matched on propensity score, year and month of initiation, and exposure to non-study opioids in the prior 90 days.

* Study period: 01 September 2014–03 December 2020.

SR, sustained release; CR, controlled release; SD, absolute standardised difference.

Covariates that were unbalanced between the two groups prior to propensity score matching were: year of initiation; age; prior cancer diagnosis; recent surgery; gabapentinoid exposure, and Hospital Frailty Risk Score. All covariates were balanced following matching.

Initiation of non-study opioids on the same day as tapentadol (SR)/oxycodone (CR) initiation differed between the two groups before and after propensity score matching (absolute standardised difference 0.36; Table 2). Immediate-release oxycodone was the most common non-study opioid dispensed, followed by codeine and tramadol. People initiating oxycodone (CR) were more likely to be dispensed immediate-release oxycodone and less likely to receive codeine and tramadol compared to people initiating tapentadol (SR) (Table 2).

**Table 2:** Initiation of non-study opioids at the same time as tapentadol (SR) or oxycodone (CR) initiation, for unmatched and propensity score matched cohorts.

	Unmatched			Matched		
	Tapentadol SR	Oxycodone CR	SD	Tapentadol SR	Oxycodone CR	SD
	*N* = 103,924	*N* = 419,732		*N* = 103,758	*N* = 103,758	
Any non-study opioid initiated	10,657 (10.3%)	98,981 (23.6%)	.36	10,646 (10.3%)	24,523 (23.6%)	.36
Type of non-study opioid initiated:						
Codeine	1,192 (11.2%)	6,677 (6.8%)	.16	1,190 (11.2%)	1,621 (6.6%)	.16
Oxycodone	8,470 (79.5%)	88,239 (89.2%)	.27	8,462 (79.5%)	22,058 (90.0%)	.29
Fentanyl	37 (0.4%)	219 (0.2%)	.02	37 (0.4%)	35 (0.1%)	.04
Hydromorphone	72 (0.7%)	179 (0.2%)	.08	72 (0.7%)	35 (0.1%)	.08
Tramadol	800 (7.5%)	5,135 (5.2%)	.10	799 (7.5%)	1,189 (4.9%)	.11
Buprenorphine^1^	238 (2.2%)	1,085 (1.1%)	.09	238 (2.2%)	260 (1.1%)	.09
Morphine	72 (0.7%)	765 (0.8%)	.01	72 (0.7%)	142 (0.6%)	.01
Methadone^1^	10 (0.1%)	18 (0.0%)	.03	10 (0.1%)	n.p.	n.p.

Note: cohort matched on propensity score, year and month of initiation, and exposure to non-study opioids in the prior 90 days.

^1^Only buprenorphine and methadone formulations with indications for pain were included in the study.

SR, sustained release; CR, controlled release; SD, absolute standardised difference; n.p., value not presented to avoid residual disclosure of small cells.

### Main analyses

There were 190 (0.2%), 302 (0.3%), and 457 (0.4%) falls among tapentadol (SR) initiators at 7, 14, and 28 days following initiation, respectively. Among oxycodone (CR) initiators, there were 330 (0.3%), 487 (0.5%), and 652 (0.6%) falls at 7, 14, and 28 days post initiation, respectively.

Following propensity score matching, risks of falls were approximately 40% lower among tapentadol (SR) initiators compared to oxycodone (CR) initiators at 7 days (relative risk [RR] 0.57, 95% CI [0.48, 0.69]) and 14 days (RR 0.62; 95% CI [0.54, 0.71]), and were 30% lower at 28 days (RR 0.70, 95% CI [0.62, 0.79]) post initiation. Adjustment for same-day initiation of non-study opioids had minimal impact on the effect estimates (Table 3). Unadjusted relative risks are presented in S7 Table.

**Table 3:** Relative risks and risk differences for falls between propensity score-matched initiators of tapentadol (SR) and oxycodone (CR), for the total study population, people aged 65+, and people aged 80+.

Outcome	Exposure	No. exposed	No. events	Matched RR^1^ (95% CI)	Adjusted^2^ RR^1^ (95% CI)	Baseline risk	Risk difference (95% CI)	Number needed to harm^3^ (95% CI)
Overall population								
7 days post-initiation	Oxycodone CR	103,758	330	0.57 (0.48,0.69)	0.57 (0.48,0.69)	0.003	−0.0014 (−0.0018, −0.0009)	739 (556,1,112)
	Tapentadol SR	103,758	190					
14 days post-initiation	Oxycodone CR	103,758	487	0.62 (0.54,0.71)	0.61 (0.53,0.71)	0.005	−0.0018 (−0.0023, −0.0013)	560 (435,770)
	Tapentadol SR	103,758	302					
28 days post-initiation	Oxycodone CR	103,758	652	0.70 (0.62,0.79)	0.68 (0.60,0.77)	0.006	−0.0019 (−0.0025, −0.0013)	529 (400,770)
	Tapentadol SR	103,758	457					
People aged 65 years and older								
7 days post-initiation	Oxycodone CR	41,546	221	0.57 (0.46,0.71)	0.57 (0.46,0.72)	0.005	−0.0023 (−0.0032, −0.0014)	436 (313,715)
	Tapentadol SR	41,546	126					
14 days post-initiation	Oxycodone CR	41,546	327	0.60 (0.50,0.72)	0.61 (0.51,0.73)	0.008	−0.0031 (−0.0042, −0.0020)	319 (239,500)
	Tapentadol SR	41,546	198					
28 days post-initiation	Oxycodone CR	41,546	451	0.67 (0.58,0.77)	0.67 (0.57,0.78)	0.011	−0.0036 (−0.0049, −0.0023)	277 (205,435)
	Tapentadol SR	41,546	303					
People aged 80 years and older								
7 days post-initiation	Oxycodone CR	10,783	122	0.62 (0.47,0.83)	0.65 (0.48,0.87)	0.011	−0.0043 (−0.0069, −0.0017)	233 (145,589)
	Tapentadol SR	10,783	76					
14 days post-initiation	Oxycodone CR	10,783	183	0.64 (0.51,0.81)	0.67 (0.52,0.85)	0.017	−0.0061 (−0.0092, −0.0029)	164 (109,345)
	Tapentadol SR	10,783	118					
28 days post-initiation	Oxycodone CR	10,783	263	0.68 (0.56,0.82)	0.70 (0.57,0.85)	0.024	−0.0079 (−0.0115, −0.0039)	127 (87,257)
	Tapentadol SR	10,783	180					

^1^Approximated by odds ratio.

^2^Conditional logistic regression models adjusted for simultaneous initiation of study and non-study opioids.

^3^Number of people that would need to be treated with oxycodone (instead of tapentadol) for one additional fall to occur.

SR, sustained release; CR, controlled release; RR, relative risk; CI, confidence interval.

We estimated a small absolute reduction in risk of falls in the whole population treated with tapentadol (SR) compared with oxycodone (CR) across all three time periods (7 days: risk difference [RD] −0.0014, 95% CI [−0.0018, −0.0009]; 14 days: RD −0.0018, 95% CI [−0.0023, −0.0013]; 28 days: RD −0.0019, 95% CI [−0.0025, −0.0013]). The number needed to treat with oxycodone (CR) rather than tapentadol (SR) for one additional fall to occur (NNTH) ranged from 739 (95% CI [556, 1,112]) at 7 days to 529 (95% CI [400, 770]) at 28 days after initiation (Table 3).

### Subgroup analysis: Older age groups

Among initiators aged 65 years and older, crude, matched, and adjusted RR estimates were similar to those of the whole population at all time points (Tables 3 and S7). Risk differences were greater in the 80 + age group (7 days: RD −0.0043, 95% CI [−0.0069, −0.0017]; 14 days: RD −0.0061, 95% CI [−0.0092, −0.0029]; 28 days: RD −0.0079, 95% CI [−0.0115, −0.0039]). The estimated NNTH values in this age group ranged from 233 (95% CI [145, 589]) at 7 days to 127 (95% CI [87, 257]) at 28 days post-initiation (Table 3).

### Subgroup analysis: Risks by exposure to non-study opioids (prior 90 days)

The patterns seen in the overall population remained in our subgroup analyses. Risks of falls were lower among tapentadol (SR) initiators compared to oxycodone (CR) initiators regardless of exposure to non-study opioids in the prior 90 days (Tables 4 and 5).

**Table 4:** Relative risks and risk differences for falls between propensity score-matched initiators of tapentadol (SR) and oxycodone (CR) with no recent exposure to non-study opioids, for the total study population, people aged 65+, and people aged 80+.

Outcome	Exposure	No. exposed	No. events	Matched RR^1^ (95% CI)	Adjusted^2^ RR^1^ (95% CI)	Baseline risk	Risk difference (95% CI)	Number needed to harm^3^ (95% CI)
Overall population								
7 days post-initiation	Oxycodone CR	65,592	202	0.59 (0.47,0.74)	0.58 (0.46,0.73)	0.003	−0.0013 (−0.0018, −0.0007)	787 (556,1,429)
	Tapentadol SR	65,592	119					
14 days post-initiation	Oxycodone CR	65,592	289	0.62 (0.52,0.75)	0.60 (0.50,0.73)	0.004	−0.0017 (−0.0023, −0.0010)	601 (435,1,000)
	Tapentadol SR	65,592	180					
28 days post-initiation	Oxycodone CR	65,592	392	0.68 (0.58,0.79)	0.64 (0.55,0.76)	0.006	−0.0019 (−0.0027, −0.0011)	522 (371,910)
	Tapentadol SR	65,592	267					
People aged 65 years and older								
7 days post-initiation	Oxycodone CR	27,056	135	0.58 (0.43,0.76)	0.57 (0.42,0.76)	0.005	−0.0021 (−0.0032, −0.0011)	472 (313,910)
	Tapentadol SR	27,056	78					
14 days post-initiation	Oxycodone CR	27,056	200	0.57 (0.45,0.72)	0.57 (0.45,0.73)	0.007	−0.0032 (−0.0045, −0.0019)	314 (223,527)
	Tapentadol SR	27,056	114					
28 days post-initiation	Oxycodone CR	27,056	279	0.63 (0.52,0.76)	0.61 (0.50,0.75)	0.010	−0.0039 (−0.0054, −0.0023)	259 (186,435)
	Tapentadol SR	27,056	176					
People aged 80 years and older								
7 days post-initiation	Oxycodone CR	6,812	86	0.54 (0.38,0.78)	0.55 (0.38,0.81)	0.013	−0.0058 (−0.0091, −0.0024)	173 (110,417)
	Tapentadol SR	6,812	47					
14 days post-initiation	Oxycodone CR	6,812	129	0.54 (0.40,0.72)	0.56 (0.41,0.75)	0.019	−0.0087 (−0.0128, −0.0047)	115 (79,213)
	Tapentadol SR	6,812	70					
28 days post-initiation	Oxycodone CR	6,812	178	0.60 (0.47,0.77)	0.62 (0.48,0.80)	0.0261	−0.0104 (−0.0150, −0.0053)	97 (67,189)
	Tapentadol SR	6,812	109					

^1^Approximated by odds ratio.

^2^Conditional logistic regression models adjusted for simultaneous initiation of study and non-study opioids.

^3^Number of people that would need to be treated with oxycodone (instead of tapentadol) for one additional fall to occur.

SR, sustained release; CR, controlled release; RR, relative risk; CI, confidence interval.

**Table 5:** Relative risks and risk differences for falls between propensity score matched initiators of tapentadol (SR) and oxycodone (CR) with exposure to non-study opioids in the 90 days prior to initiation, for the total study population, people aged 65+, and people aged 80+.

Outcome	Exposure	No. exposed	No. events	Matched RR^1^ (95% CI)	Adjusted^2^ RR^1^ (95% CI)	Baseline risk	Risk difference (95% CI)	Number needed to harm^3^ (95% CI)
Overall population								
7 days post-initiation	Oxycodone CR	38,166	128	0.56 (0.42,0.74)	0.57 (0.42,0.76)	0.003	−0.0015 (−0.0022, −0.0008)	671 (455,1,250)
	Tapentadol SR	38,166	71					
14 days post-initiation	Oxycodone CR	38,166	198	0.61 (0.49,0.77)	0.62 (0.49,0.78)	0.005	−0.0020 (−0.0029, −0.0011)	500 (345,910)
	Tapentadol SR	38,166	122					
28 days post-initiation	Oxycodone CR	38,166	260	0.73 (0.61,0.88)	0.73 (0.60,0.88)	0.007	−0.0018 (−0.0029, −0.0007)	544 (345,1,429)
	Tapentadol SR	38,166	190					
People aged 65 years and older								
7 days post-initiation	Oxycodone CR	14,490	86	0.56 (0.39,0.80)	0.58 (0.41,0.83)	0.006	−0.0026 (−0.0044, −0.0012)	382 (228,834)
	Tapentadol SR	14,490	48					
14 days post-initiation	Oxycodone CR	14,490	127	0.65 (0.49,0.86)	0.67 (0.51,0.89)	0.009	−0.0030 (−0.0050, −0.0010)	329 (200,1,000)
	Tapentadol SR	14,490	84					
28 days post-initiation	Oxycodone CR	14,490	172	0.73 (0.58,0.93)	0.75 (0.59,0.94)	0.012	−0.0032 (−0.0055, −0.0008)	317 (182,1,250)
	Tapentadol SR	14,490	127					
People aged 80 years and older								
7 days post-initiation	Oxycodone CR	3,971	36	0.81 (0.49,1.31)	0.88 (0.53,1.45)	0.009	−0.0018 (−0.0059,0.0023)	569 (NNTH 170 to ∞ and NNTB 434 to ∞)
	Tapentadol SR	3,971	29					
14 days post-initiation	Oxycodone CR	3,971	54	0.89 (0.60,1.32)	0.94 (0.62,1.42)	0.014	−0.0016 (−0.0066,0.0035)	640 (NNTH 152 to ∞ and NNTB 285 to ∞)
	Tapentadol SR	3,971	48					
28 days post-initiation	Oxycodone CR	3,971	85	0.83 (0.60,1.14)	0.84 (0.60,1.16)	0.0214	−0.0036 (−0.0097,0.0026)	277 (NNTH 104 to ∞ and NNTB 384 to ∞)
	Tapentadol SR	3,971	71					

1 Approximated by odds ratio.

2 Conditional logistic regression models adjusted for simultaneous initiation of study and non-study opioids.

3 Number of people that would need to be treated with oxycodone (instead of tapentadol) for one additional fall to occur.

SR, sustained release; CR, controlled release; RR, relative risk; CI, confidence interval; NNTH, number of people that need to be treated for one additional person to be harmed; NNTB, number of people that need to be treated for one additional person to benefit.

The lowest risks (and largest risk differences) were among opioid-naïve adults initiating tapentadol (SR) and aged 80 years and over (Table 4), with estimated NNTH ranging from 173 (95% CI [110, 417]) to 97 (95% CI [67, 189]) at 7 and 28 days post-initiation, respectively. However, associations were attenuated among initiators aged 80+ years with exposure to opioids in the prior 90 days (Table 5).

### Sensitivity analyses

Results of our sensitivity analyses were consistent with those of our primary analysis (S9–S13, and S19 Tables).

There was no notable difference in the average follow-up time between the tapentadol (SR) and oxycodone (CR) groups (S14–S16 Tables; S3–S5 Figs).

The E-value for falls indicated that an unmeasured confounder would have to have a RR ≥ 2.9 with both exposure and falls, after adjustment for all other measured confounders, to explain away the observed RR (0.57) at 7 days post-initiation. A confounder with RR ≥ 2.3 would cause the confidence interval to include 1. At 28 days post-initiation, slightly weaker confounder relationships would explain away the observed associations; an unmeasured confounder with RR ≥ 2.2 would explain away the observed RR (0.70) and one with RR ≥ 1.9 would cause the confidence interval to include 1 (S17 Table). We found similar results among our 65+ and 80+ population groups.

We did not find any differences in risks of cataract surgeries (negative control outcome) between tapentadol (SR) and oxycodone (CR) initiators at any time point or among any of our population groups (S18 Table).

Nearly everyone experiencing a fall presented to hospital or emergency department settings; very few falls resulted in death (S19 Table).

## Discussion

In our population-based cohort study, we found significantly lower risks of falls resulting in ED presentations, hospitalisations, or death among people initiating tapentadol (SR) compared to those of people initiating oxycodone (CR). Tapentadol (SR) was associated with an approximately 40% lower risk of falls within the first 14 days following initiation and a 30% lower risk within 28 days of initiation. However, absolute overall risk reductions were small, with numbers-needed-to-treat with oxycodone (CR) instead of tapentadol (SR) for one additional fall to occur of 739 and 529 people at 7 and 28 days post-initiation, respectively. We found greater reductions in fall risks among older people (aged 65+ years) who were opioid-naïve. The greatest absolute reductions in fall risks were seen in opioid-naïve people aged 80+, with NNTH values for one additional fall to occur ranging from 173 to 97 people (7 and 28 days post-initiation, respectively). However, fall risks were attenuated among people aged 80+ years who had exposure to other opioids in the 90 days prior to tapentadol (SR)/oxycodone (CR) initiation.

Our results are consistent with findings from randomised clinical trials. Lower rates of central nervous system effects in tapentadol (SR) treatment arms have been reported in head-to-head comparisons with oxycodone (CR) [3,6,7]. This suggests plausibility for lower risks of falls associated with tapentadol (SR) initiation, which are likely to be mediated through these nervous system effects, particularly those affecting balance. Interestingly, Liu and colleagues [47] found that tapentadol (any formulation) was associated with higher post-surgical risks of delirium compared to oxycodone (any formulation). Falls are multi-factorial and delirium is one of many potential considerations. However, delirium occurring in the immediate post-surgical period may not necessarily influence falls occurring post-discharge (the focus of our study).

Among the older age groups (65+ and 80+ years) in our study, we found that fall risks differed depending on prior exposure to non-study opioids; effect sizes were largest among people who were opioid-naïve. The risk of serious falls is highest in the first 28 days following initiation [14]; accordingly, if the true effect of tapentadol (SR) was indeed protective (in comparison to oxycodone (CR)), then presumably this would be most evident in an opioid-naïve population. Furthermore, it is reasonable to assume that a true protective effect would be more pronounced among opioid-naïve older adults where the risk of falls is highest [14], which is consistent with our findings. We observed some attenuation of associations among older initiators (particularly people in the 80+ group) with previous exposure to non-study opioids (prior 90 days), which suggests that existing opioid tolerance may diminish any protective benefits conferred by tapentadol (SR).

This study leverages linked population-level data to assess outcomes of tapentadol (SR) and oxycodone (CR) in a real-world clinical setting, which includes people who are prescribed multiple opioids to manage pain. To mitigate the possibility that people prescribed tapentadol (SR) represent a comparatively ‘healthier’ cohort than people prescribed oxycodone (CR), we conducted a new user active comparator study, included a substantial list of potential confounders in the propensity score, performed cohort restriction (according to prior and concurrent use of non-study opioids) and included a suite of sensitivity analyses and a negative control outcome. We used exact and propensity score matching rather than other approaches such as inverse probability weighting or stratification because it better accounted for changes over time in opioid use and prescribing policies during the study period.

This study had several potential limitations. First, data on dispensing of immediate-release (IR) tapentadol were not available, so people who appeared to have no opioid exposure or concurrent initiation of non-study opioids may have purchased IR tapentadol privately and be therefore misclassified. This may differentially impact our exposure groups, as people prescribed tapentadol (SR) may have been more likely to have also been prescribed tapentadol (IR). However, our findings were consistent across our main and sensitivity analyses, suggesting that the impact of misclassification was minimal. Furthermore, E-values suggested that an unmeasured confounder would require a relatively strong association with both tapentadol (SR) exposure and falls after adjustment for all measured confounders to explain away the observed relative risks. It is unlikely that tapentadol (IR) would have such a strong positive association where tapentadol (SR) appeared to have a protective effect. Nonetheless, it is critical to replicate this analysis in other data, particularly where information on tapentadol (IR) is available. Second, the data only permit observation of dispensings, but not whether medicines were actually ingested or taken as prescribed. Third, information on dose was not available in our data, and the impact of dose requires further study. Fourth, we were unable to assess duration of therapy and emulated an intention-to-treat analysis. Fifth, we were unable to completely ascertain previous history of falls; we did not have access to self-report or primary care records, which would have captured falls not resulting in ED attendance or hospital admission. This may affect the generalisability of our findings. However, the degree of under-ascertainment should be unrelated to the treatment, and thus should not affect the internal validity of the results. Sixth, some variables may be subject to misclassification or incomplete capture (e.g., history of falls). Seventh, we only assessed the first fall in the four weeks following opioid initiation; future studies with longer follow-up duration should investigate repeated fall events. Lastly, our study endpoint captures severe falls resulting in contact with the healthcare system and does not capture the full health and social impact of all falls that may have occurred following study opioid initiation. Despite these limitations, our results were consistent across our main, subgroup and sensitivity analyses with largely constant effect sizes, and found no association between our choice of opioid and our negative control outcome (cataract surgery), lending further credibility to our findings.

Falls can have devastating consequences, including mortality and life-long impacts on independence and mobility. In light of trial evidence demonstrating that tapentadol (SR) and oxycodone (CR) achieve comparable analgesic effects and improvements in functionality and quality of life [4,9], tapentadol (SR) may be a safer (yet equally effective) choice of pain relief, particularly among older people where the consequences of falls are most severe. Indeed, this may be most pertinent for opioid-naïve people aged 80 years and older, where less serious fall events may affect confidence in walking, subsequently reducing mobility and quality of life and increasing social costs; tapentadol (SR) may be preferable over oxycodone (CR) in this group. Clinicians should also consider the interaction between prior opioid exposure and underlying fall risks when making opioid prescribing decisions. In a broader context, however, falls are only one of many aspects that require consideration when choosing to prescribe opioids.

Falls are the leading cause of hospitalisations and deaths due to injury, accounting for nearly a quarter of a million hospitalisations and 6,700 deaths, and costing approximately AUD $5 billion [48,49] in 2023/24. Although the risk reductions we found were small, with 3.1 million Australians [50] estimated to use opioids (the majority of which is tapentadol and oxycodone [20]), and in light of the potential severity of falls outcomes, these small reductions could translate to fewer impacts on morbidity (via fewer falls) and lead to substantial health system savings.

In this real-world study comparing initiators of tapentadol (SR) and oxycodone (CR), including people who use other opioids alongside the opioids of interest, we found that tapentadol (SR) was associated with a lower risk of falls resulting in ED presentation, hospitalisation, or death. Although the estimated absolute difference in risk between tapentadol (SR) and oxycodone (CR) users was small, given the widespread use of opioids this may translate to a substantial reduction in falls and associated impacts on morbidity and costs. If the effect is causal, tapentadol (SR) may be a safer and equally effective choice of opioid, particularly among older people where the consequences of falls are most severe.

## Supporting information

S1 Checklist The Reporting of Studies Conducted using Observational Routinely-collected health Data (RECORD) checklist. Available from: https://www.record-statement.org/checklist.php.
(PDF)

S1 Table Data sources. (PDF)

S2 Table ATC and PBS item codes for identifying study opioids. (PDF)

S3 Table Outcome definitions. (PDF)

S4 Table Covariate definitions. (PDF)

S5 Table Baseline characteristics of tapentadol (SR) and oxycodone (CR) initiators aged 65 years and older, 1 September 2014–3 December 2020, before and after propensity score matching. (PDF)

S6 Table Baseline characteristics of tapentadol (SR) and oxycodone (CR) initiators aged 80 years and older, 1 September 2014–3 December 2020, before and after propensity score matching. (PDF)

S7 Table Unadjusted relative risks of falls among people initiating tapentadol (SR) compared to oxycodone (CR), overall and by recent exposure to non-study opioids, for the total study population, people aged 65+, and people aged 80+. (PDF)

S8 Table Baseline characteristics of tapentadol (SR) and oxycodone (CR) initiators with only study opioids dispensed on initiation date, 1 September 2014–3 December 2020, before and after propensity score matching. (PDF)

S9 Table Relative risks of falls and cataract surgeries among tapentadol (SR) and oxycodone (CR) initiators with only study opioids dispensed on initiation date. (PDF)

S10 Table Baseline characteristics of tapentadol (SR) and oxycodone (CR) initiators with only study opioids dispensed on initiation date and no exposure to non-study opioids in the previous 90 days, before and after propensity score matching. (PDF)

S11 Table Relative risks of falls and cataract surgeries among tapentadol (SR) and oxycodone (CR) initiators with only study opioids dispensed on initiation date and no exposure to non-study opioids in the prior 90 days. (PDF)

S12 Table Baseline characteristics of tapentadol (SR) and oxycodone (CR) initiators between 1 September 2014–3 December 2020 who concurrently initiated oxycodone (IR), before and after propensity score matching. (PDF)

S13 Table Relative risks of falls and cataract surgeries among tapentadol (SR) and oxycodone (CR) initiators who concurrently initiated oxycodone (IR). (PDF)

S14 Table Average follow-up time (days), by exposure. (PDF)

S15 Table Average follow-up time (days), by exposure, among initiators with no recent opioid exposure. (PDF)

S16 Table Average follow-up time (days), by exposure, among initiators with recent exposure to opioids. (PDF)

S17 Table E-values for relative risks of falls. (PDF)

S18 Table Relative risks of cataract surgeries (negative control outcome) for the total study population, people aged 65+, and people aged 80+. (PDF)

S19 Table Event rates and relative risks of falls, by source of presentation (ED visit, hospital admission, death), for the total study population. (PDF)

S1 Fig Study design. (PDF)

S2 Fig Propensity score distributions for tapentadol (SR) and oxycodone (CR) initiators. (PDF)

S3 Fig Cumulative incidence curves: falls following initiation of tapentadol (SR) or oxycodone (CR). (PDF)

S4 Fig Cumulative incidence curves: falls following initiation of tapentadol (SR) or oxycodone (CR) among initiators with no recent opioid exposure. (PDF)

S5 Fig Cumulative incidence curves: falls following initiation of tapentadol (SR) or oxycodone (CR) among initiators with recent exposure to opioids. (PDF)

S1 Protocol Study protocol. (PDF)

## Abbreviations

ACHI: Australian Classification of Health Interventions
AIHW HREC: Australian Institute of Health and Welfare Human Research Ethics Committee
CR: controlled-release
ED: emergency department
FRIDs: falls-risk medicines
ICD: International Classification of Diseases
IR: immediate-release
NNTH: number-needed-to-treat-to-harm
NRI: noradrenaline reuptake inhibitor
NSW: New South Wales
OME: oral morphine equivalent milligrams
PBS: Pharmaceutical Benefits Scheme
PHSREC: Population and Health Services Research Ethics Committee
RD: risk difference
RECORD: Reporting of studies Conducted using Observational Routinely-collected health Data
RR: relative risks
SNOMED-CT-AU: Systematized Nomenclature of Medicine-Clinical Terms-Australian version
SR: sustained-release
