# Supplementary material for: Short-term risk of falls among initiators of controlled-release tapentadol versus oxycodone in New South Wales between 2014 and 2020: A population-based retrospective cohort study
Source: PLoS Med. 2026 Aug 11;23(8):e1004950. doi: 10.1371/journal.pmed.1004950 (PMC13460682; doi:10.1371/journal.pmed.1004950)
Supplement: Supplementary file 2 [file pmed.1004950.s002.pdf]

S1 Table. Data sources

This study leveraged the Medicines Intelligence Data Platform, held by the Medicines Intelligence Research Program at the School of Population Health, UNSW Sydney and described elsewhere[1]. The Medicines Intelligence Data Platform comprises longitudinal, linked population-level data for adults residing in NSW between 2005 and 2020 who were eligible for Medicare (i.e., registered in the Medicare Consumer Directory).

Data dictionaries for NSW data are available from the Centre for Health Record Linkage (CHeReL)[2]. The CHeReL supports secure access to linked health data about people in New South Wales (NSW) and the Australian Capital Territory (ACT) for researchers, planners, and policy makers. The CHeReL is a node of the Population Health Research Network, which is an Australian Government initiative being conducted as part of the National Collaborative Research Infrastructure Strategy. The core functions of the CHeReL are funded by the NSW Ministry of Health.

Information on Commonwealth data collections are available from the Australian Institute for Health and Welfare (AIHW)[3]. The AIHW is a statutory Australian Government agency providing information and statistics to support policy and service delivery decisions.

Data source	Description
NSW data collections	
NSW Admitted Patient Data Collection (APDC)	A statutory data collection containing records of all hospital separations (including public hospitals, public psychiatric hospitals, public multi-purpose services, private hospitals, and private day procedure centres) in NSW. Data on diagnoses are coded according to the International Classification of Diseases, Version 10, Australian Modification (ICD-10-AM) and procedures coded according to the Australian Classification of Health Interventions (ACHI).
NSW Emergency Department Data Collection (EDDC)	A data collection containing all visits to participating emergency departments in NSW. Data on diagnoses are coded according to the International Classification of Diseases, Version 9/10 or SNOMED-CT-AU.
NSW Cancer Registry (NSWCR)	A statutory data collection containing all notifications of primary malignant neoplasms from 1 Jan 1972.
Commonwealth data collections	
Medicare Consumer Directory (MCD)	Listing of individuals registered with Medicare (Australian citizens, permanent residents, and foreigners with reciprocal health care arrangements).
Pharmaceutical Benefits Scheme (PBS)[4]	Claims for dispensed prescription medicines covered under the Pharmaceutical Benefits Scheme. All Medicare-eligible residents are eligible to receive medicines subsidised by the PBS. The PBS covers dispensings from community pharmacies and private hospitals; information on medicines provided in acute care, emergency departments and by private prescriptions is not available.
National Death Index (NDI)[5]	Listing of all deaths (with cause of death information) registered in Australia since 1980.

NSW = New South Wales; SNOMED-CT-AU: Systematized Nomenclature of Medicine-Clinical Terms-Australian version.

REFERENCES

1. Zoega H, Falster MO, Gillies MB, Litchfield M, Camacho X, Bruno C, et al. The Medicines Intelligence Data Platform: A Population-Based Data Resource From New South Wales, Australia. Pharmacoepidemiol Drug Saf. 2024;33(8):e5887. doi: 10.1002/pds.5887. PubMed PMID: 39145404.
2. Datasets: Centre for Health Record Linkage; 2025 [accessed: 16 October 2025]. Available from: https://www.cherel.org.au/datasets.
3. Our data collections: Australian Institute of Health and Welfare; 2025 [accessed: 16 October 2025]. Available from: https://www.aihw.gov.au/about-our-data/our-data-collections.
4. Pharmaceutical Benefits Scheme data collection: Australian Institute of Health and Welfare; 2025 [accessed: 16 October 2025]. Available from: https://www.aihw.gov.au/about-our-data/our-data-collections/pharmaceutical-benefits-scheme.
5. National Death Index: Australian Institute of Health and Welfare; 2025 [accessed: 16 October 2025]. Available from: https://www.aihw.gov.au/about-our-data/our-data-collections/national-death-index.
