# Supplementary material for: Short-term risk of falls among initiators of controlled-release tapentadol versus oxycodone in New South Wales between 2014 and 2020: A population-based retrospective cohort study
Source: PLoS Med. 2026 Aug 11;23(8):e1004950. doi: 10.1371/journal.pmed.1004950 (PMC13460682; doi:10.1371/journal.pmed.1004950)
Supplement: Supplementary file 3 [file pmed.1004950.s003.pdf]

S2 Table. ATC and PBS item codes for identifying study opioids

All exposures are ascertained from the Pharmaceutical Benefits Scheme (PBS) dispensing records.

Exposure	ATC code	PBS item codes
Tapentadol (SR)	N02AX06	10096J, 10094G, 10100N, 10091D, 10092E
Oxycodone (CR)	N02AA05	08385H, 08386J, 08387K, 08388L, 08681X, 09399Q, 09400R, 12510K, 12518W, 12525F, 12527H, 12538X, 12545G, 05015Y, 05016B, 05227D, 05247E, 05248F, 05249G, 05250H, 06058W, 06059X, 06060Y, 06061B, 06079Y, 07215R, 07216T
	N02AA55	08000C, 08934F, 08935G, 08936H, 10757E, 10758F, 10776E, 11102H, 11111T, 12471J, 12475N, 12486E, 12498T, 12511L, 12522C, 12523D, 12532N, 12540B

ATC: Anatomical Therapeutic Chemical classification; PBS: Pharmaceutical Benefits Scheme.
