# Supplementary material for: Short-term risk of falls among initiators of controlled-release tapentadol versus oxycodone in New South Wales between 2014 and 2020: A population-based retrospective cohort study
Source: PLoS Med. 2026 Aug 11;23(8):e1004950. doi: 10.1371/journal.pmed.1004950 (PMC13460682; doi:10.1371/journal.pmed.1004950)
Supplement: Supplementary file 4 [file pmed.1004950.s004.pdf]

S3 Table. Outcome definitions

Falls

Falls were defined as any hospitalisation, emergency department presentation, or death related to a fall, occurring during the follow-up period[1-4].

Hospital data: any ICD-10-AM code in any diagnosis field (principal or other) where the fall was not classified as an in-hospital event (i.e., condition onset flag ^= 1 [condition with onset during the episode of care]).

Emergency department data: any ICD-9 or SNOMED-CT-AU code in any diagnosis field.

Death data: any ICD-10 code in any cause of death field (underlying or other cause of death).

Cataract surgery

Cataract surgeries were defined as any hospitalisation related to cataract surgery occurring during the follow-up period. A procedure block or code in any position was sufficient to ascertain the event of interest[5].

Outcome	Data source	Codes
Falls resulting in hospitalisation, ED presentation, or death	ICD-10-AM code in EDDC or APDC	W00, W01, W02, W03, W04, W05, W06, W07, W08, W09, W10, W11, W12, W13, W14, W15, W16, W17, W18, W19
	ICD-10 code in death records	W00, W01, W02, W03, W04, W05, W06, W07, W08, W09, W10, W11, W12, W13, W14, W15, W16, W17, W18, W19
	ICD-9 code in EDDC	E880, E881, E882, E883, E884, E885, E886, E887, E888

Outcome	Data source	Codes
	SNOMED-CT-AU code in EDDC	298343000, 249994007, 298345007, 61683000, 161898004, 252318014, 298344006, 439017019, 408561005, 720141000168100, 249997000, 298346008, 439020010, 298347004, 439021014, 279992002, 417481019, 404911003, 2156786012, 249995008, 1144480005, 404912005, 2156787015, 363801007, 363802000, 298348009, 427206005, 398117008, 1889000100004100, 32121000119107, 242177005, 218250005, 1912002, 4301012, 217082002, 329287012, 329286015, 715157007, 715158002, 68062003, 13935005, 242399008, 242400001, 242401002, 242398000, 362715011, 57741007, 217173005, 217147000, 217106003, 242413007, 242417008, 20902002, 83468000, 138437018, 217142006, 242415000, 713397003, 242414001, 56307009, 242419006, 17886000, 217148005, 242126003, 429482004, 86591008, 217097004, 84026000, 242408008, 14047009, 217103006, 242409000, 242410005, 217107007, 217108002, 217112008, 242411009, 4521008, 242412002, 2617007, 217110000, 41411008, 72738009, 78331008, 13711007, 13278006, 217150002, 82947003, 217109005, 90639005, 40104005, 66810014, 74541001, 90619006, 44188002, 439570003, 216107001, 216108006, 216116002, 216111007, 216117006, 216113005, 216110008, 216115003, 216119009, 216123001, 216129002, 216125008, 216122006, 216127000, 216128005, 216154002, 242228005, 242227000, 216157009, 216163000, 216159007, 216156000, 216161003, 216162005, 216131006, 216135002, 216141009, 216137005, 216134003, 216140005, 242423003, 217130001, 68274007, 217124001, 217126004, 242420000, 242421001, 242426006, 242428007, 242427002, 217132009, 217133004, 217134005, 242425005, 242424009, 217135006, 242422008, 217123007, 39109007, 78331008, 242182003, 242183008, 242184002, 215644005, 215651001, 215646007, 215645006, 215650000, 215634008, 215641002, 215636005, 215635009, 215640001, 242185001, 75354000, 217083007, 217084001, 217086004, 217088003, 217090002, 414188008, 242109009, 214436006, 214437002, 214439004, 214441003, 214438007, 214443000, 214444006, 214442005, 414190009, 217092005, 217094006, 2534632013, 217093000, 242120009, 242122001, 242121008, 242123006, 33036003, 274919008, 288296009, 242402009, 217161005, 217162003, 274918000, 242397005, 217155007, 242391006, 242390007, 242387001, 242388006, 242389003, 242392004, 217157004, 217156008, 242394003, 242393009, 242395002, 242396001, 217158009, 269699007, 242404005, 242405006, 242406007, 242407003, 217154006, 215633002, 418111002, 48015001, 60594001, 242125004, 225054009, 78361000, 54670004, 82612009, 75941004, 213911003, 213925002, 242078003, 242079006, 213927005, 213926001, 213912005, 213914006, 213913000, 213917004, 213919001, 213922004, 213920007, 213918009, 429636000, 427849003, 429621003, 67223001, 56962005, 429012000, 215295004, 215298002, 215296003, 215297007, 242111000, 242112007, 214447004, 214448009, 214450001, 214452009, 214449001, 214455006, 214453004, 214538005, 214993007, 215227000, 1889000100004102, 217924002, 414189000

Outcome	Data source	Codes
Cataract surgery	ACHI block in APDC	Data years 2015/16, 2016/17: 195, 196, 197, 198, 199, 200, 201<br>Data years 2017/18: 200<br>Data years 2018/19: 200<br>Data years 2019/20, 2020/21: 200
	ACHI procedure code in APDC	Data years 2014/15: 42698-00 to 42698-05, 42702-00 to 42702-11, 42716-00, 42719-00, 42719-02, 42722-00, 42731-00, 42731-01, 42734-00, 42788-00<br>Data years 2017/18: 42734-01, 90077-00<br>Data years 2018/19: 42737-01, 42734-01<br>Data years 2019/20, 2020/21: 42737-01, 42734-01

APDC: Admitted Patient Data Collection; ACHI: Australian Classification of Health Interventions; EDDC: Emergency Department Data Collection; ICD-9: International Statistical Classification of Diseases and Related Health Problems, 9th Revision; ICD-10: International Classification of Diseases, 10th Revision; ICD-10-AM: International Classification of Diseases, 10th Revision, Australian Modification; SNOMED-CT-AU: Systematized Nomenclature of Medicine-Clinical Terms-Australian version.

REFERENCES

1. ICD-10 Version:2019: WHO; [accessed: 16 October 2025]. Available from: https://icd.who.int/browse10/2019/en.
2. Moreland BL, Burns ER, Haddad YK. National rates of non-fatal emergency department visits and hospitalisations due to fall-related injuries in older adults 2010-2014 and 2016: transitioning from ICD-9-CM to ICD-10-CM. Inj Prev. 2021;27(S1):i75-i8. doi: 10.1136/injuryprev-2019-043516. PubMed PMID: 33674338; PubMed Central PMCID: PMCPMC7948188.
3. Hopkins RE, Bharat C, Buizen L, Close J, Ivers R, Draper B, et al. Age-Related Risk of Serious Fall Events and Opioid Analgesic Use. JAMA Intern Med. 2024;184(4):394-401. doi: 10.1001/jamainternmed.2023.8154. PubMed PMID: 38373005; PubMed Central PMCID: PMCPMC10877504.
4. Australian Institute of Health and Welfare. Injury in Australia: Falls: AIHW; 2025 [updated 24 June 2025 accessed: 14 November 2025]. Available from: https://www.aihw.gov.au/reports/injury/falls.
5. Hospitals info & downloads: Australian Institute of Health and Welfare; 2024 [accessed: 10 July 2024]. Available from: https://www.aihw.gov.au/reports-data/myhospitals/content/data-downloads
