# Supplementary material for: Short-term risk of falls among initiators of controlled-release tapentadol versus oxycodone in New South Wales between 2014 and 2020: A population-based retrospective cohort study
Source: PLoS Med. 2026 Aug 11;23(8):e1004950. doi: 10.1371/journal.pmed.1004950 (PMC13460682; doi:10.1371/journal.pmed.1004950)
Supplement: Supplementary file 5 [file pmed.1004950.s005.pdf]

S4 Table. Covariate definitions

Relative disadvantage: We used a relative area-based socio-economic index based on Census data (Index of Relative Socio-Economic Disadvantage; IRSD) that summarises a range of information about economic and social conditions. The score is based on a combination of factors such as income, education, employment, housing, and family structure. Scores are calculated for each Statistical Area; deciles are then created by ordering the areas by disadvantage and dividing into ten equally sized groups, with decile 1 containing the most disadvantaged areas and decile 10 the most advantaged[1]. For each initiator, we used the Socio-Economic Index for Australia (SEIFA) correspondence file from the Australian Bureau of Statistics (ABS) to map the postal code at initiation to the corresponding IRSD decile (2016 SEIFA index).

Remoteness: We defined remoteness using the Australian Statistical Geographic Standard Remoteness Structure[2]. This structure divides Australia into five classes of remoteness characterised by relative geographic access to services, measured using the Accessibility/Remoteness Index of Australia Plus (ARIA+)[3]. For each initiator, we used an ABS correspondence file to map the postal code at the time of initiation to the corresponding remoteness area (2016 Remoteness Area structure).

Hospital frailty risk score: We defined baseline frailty using the Hospital Frailty Risk Score defined by Gilbert et al.[4]. The Hospital Frailty Risk Score is a validated method of deriving a frailty score from routinely-collected inpatient data, using ICD-10 diagnosis codes with individual weightings. We grouped the frailty score into three levels (low: <5, medium: 5-15, and high: >15), where the score was calculated using diagnosis codes from all inpatient admissions in the APDC from the previous 24 months[5].

Recent surgeries: We ascertained recent surgeries as per previous studies using the Medicines Intelligence Data Platform[6]. We defined recent surgeries using any inpatient hospital procedure with an ACHI surgical procedure code corresponding to ACHI code blocks identified in Gong et al.[7] in the 30 days prior to study opioid initiation. Defined as binary (1 = recent surgery, 0 = no recent surgery).

History of falls: We ascertained previous falls in the 2 years to 61 days (inclusive) prior to study opioid initiation. We looked for evidence of falls resulting in ED presentation or hospitalisation (defined as in the outcome; S3 Table), defined as a binary indicator (1 = previous fall, 0 = no previous fall).

Exposure to falls-risk medicines: We used an established list of Falls-Risk-Increasing Drugs (FRIDs) that has been used extensively in the literature and includes medicines known to increase the risk of falls and to cause orthostatism and hypotension[8, 9]. Defined as binary (1 = recent exposure to FRIDs, 0 = no recent exposure to FRIDs).

Covariates were ascertained as per the table below. Unless otherwise specified, diagnosis codes could occur in any position and any combination of codes present during the lookback window were sufficient to ascertain the condition or exposure of interest.

Covariate	Data source	Codes
Comorbidities		
Cancer<br>(Dichotomous: 1 = yes, 0 = no)	NSW Cancer Registry	Between (initiation - 365) and (initiation - 1):<br>any record with diagnosis date within the ascertainment period
	ICD-10-AM code in EDDC or APDC	Between (initiation - 365) and (initiation - 1):<br>C00-C26, C30-C34, C37- C41, C43-C58, C60- C76, C81-C85, C88, C90-C97, Z51.1
	ATC code in PBS	Between (initiation - 365) and (initiation - 1):<br>L01AA01-L01AX04, L01BA01, L01BA03- L01XX53, L02BG03, L02BG04, L02BG06, L02BB01-L02BB04, L02BX01-L02BX03, L04AX02, L04AX04, L04AX06, L02BA01, L02AE03, L02AE02
History of substance use disorder<br>(Dichotomous: 1 = yes, 0 = no)	ICD-10-AM code in EDDC or APDC	Between (initiation - 365) and (initiation - 1):<br>F11, F12-F19, Z71.5, Z72.2
	ATC code in PBS	Between (initiation - 365) and (initiation - 1):<br>N07B
History of overdose<br>(Dichotomous: 1 = yes, 0 = no)	ICD-10-AM code in EDDC or APDC	Between (initiation - 365) and (initiation - 1):<br>T40.0-T40.9, T42.3, T42.4, T42.6, T42.7, T43.6, T43.9, T51.0-T51.3, T51.8, T51.9, T52.0-T52.4, T52.8, T52.9, T53.0-T53.9
Depression<br>(Dichotomous: 1 = yes, 0 = no)	ICD-10-AM code in EDDC or APDC	Between (initiation - 365) and (initiation - 1):<br>F20.4, F31.3-F31.5, F32, F33, F34.1, F41.2, F43.2, U79.3
	ATC code in PBS	Between (initiation - 365) and (initiation - 1):<br>N06A
Anxiety<br>(Dichotomous: 1 = yes, 0 = no)	ICD-10-AM code in EDDC or APDC	Between (initiation - 365) and (initiation - 1):<br>F40-F41, F43.22, F43.23
	ATC code in PBS	Between (initiation - 365) and (initiation - 1):<br>N05BA01–N05BA12, N05BE01

Covariate	Data source	Codes
Hypertension<br>(Dichotomous: 1 = yes, 0 = no)	ICD-10-AM code in EDDC or APDC	Between (initiation - 365) and (initiation - 1):<br>I10-I15, U82.3
	ATC code in PBS	Between (initiation - 365) and (initiation - 1):<br>C02AB01-C02AC05, C02DB02-C02KX01
Atrial fibrillation / flutter<br>(Dichotomous: 1 = yes, 0 = no)	ICD-10-AM code in EDDC or APDC	Between (initiation - 365) and (initiation - 1):<br>I48
Diabetes[10, 11]<br>(Dichotomous: 1 = yes, 0 = no)	ICD-10-AM code in EDDC or APDC	Between (initiation - 365) and (initiation - 1):<br>E10-E14 (excl E12)
	ATC code in PBS	Between (initiation - 365) and (initiation - 1):<br>A10AA-A10AE, A10BA02, A10BD02, A10BD03, A10BD07, A10BD08, A10BD10, A10BD11, A10BD13, A10BB, A10BH, A10BD19, A10BD21, A10BD24, A10BG, A10BF
Congestive heart failure[11]<br>(Dichotomous: 1 = yes, 0 = no)	ICD-10-AM code in EDDC or APDC	Between (initiation - 365) and (initiation - 1):<br>I50
	ATC code in PBS	Between (initiation - 365) and (initiation - 1):<br>C03DA; C07AB02, C07AB07, C07AG02, C07AB12; (C03C) AND (C09A OR C09C)
Thyroid disease<br>(Dichotomous: 1 = yes, 0 = no	ICD-10-AM code in EDDC or APDC	Between (initiation - 365) and (initiation - 1):<br>E00-E07, E89.0
	ATC code in PBS	Between (initiation - 365) and (initiation - 1):<br>H03BA02 - H03BB01, H03AA01 - H03AA02
Postural hypotension<br>(Dichotomous: 1 = yes, 0 = no)	ICD-10-AM code in EDDC or APDC	Between (initiation - 365) and (initiation - 1):<br>I95.1
Osteoporosis[12]<br>(Dichotomous: 1 = yes, 0 = no)	ICD-10-AM code in EDDC or APDC	Between (initiation - 365) and (initiation - 1):<br>M80, M81, M82

Covariate	Data source	Codes
	ATC code in PBS	Between (initiation - 365) and (initiation - 1):<br>M05B
Stroke[13]<br>(Dichotomous: 1 = yes, 0 = no)	ICD-10-AM code in EDDC or APDC	Between (initiation - 365) and (initiation - 1):<br>I60, I61, I62, I64
	ATC code in PBS	Between (initiation - 365) and (initiation - 1):<br>B01A
Renal impairment[14]<br>(Dichotomous: 1 = yes, 0 = no)	ICD-10-AM code in EDDC or APDC	Between (initiation - 365) and (initiation - 1):<br>I12.0, I13.1, N03.2 - N03.7, N05.2 - N05.7, N18, N19, N25.0, Z49.0 - Z49.2, Z94.0, Z99.2
	ATC code in PBS	Between (initiation - 365) and (initiation - 1):<br>B03XA01-B03XA02, A11CC01-A11CC04, V03AE02
Hepatic impairment[14]<br>(Dichotomous: 1 = yes, 0 = no)	ICD-10-AM code in EDDC or APDC	Between (initiation - 365) and (initiation - 1):<br>B18, K70.0 - K70.3, K70.9, K71.3 - K71.5, K71.7, K73, K74, K76.0, K76.2 - K76.4, K76.8, K76.9, Z94.4
	ATC code in PBS	Between (initiation - 365) and (initiation - 1):<br>A06AD11
Hospital Frailty Risk Score		
Baseline frailty	ICD-10-AM code in APDC	Between (initiation - 730) and (initiation - 1):<br>A04, A09, A41, B95, B96, D64, E05, E16, E53, E55, E83, E86, E87, F00, F01, F03, F05, F10, F32, G20, G30, G31, G40, G45, G81, H54, H91, I63, I67, I69, I95, J18, J22, J69, J96, K26, K52, K59, K92, L03, L08, L89, L97, M15, M19, M25, M41, M48, M79, M80, M81, N17, N18, N19, N20, N28, N39, R00, R02, R11, R13, R26, R29, R31, R32, R33, R40, R41, R44, R45, R47, R50, R54, R55, R56, R63, R69, R79, R94, S00, S01, S06, S09, S22, S32, S42, S51, S72, S80, T83, U80, W01, W06, W10, W18, W19, X59, Y84, Y95, Z22, Z50, Z60, Z73, Z74, Z75, Z87, Z91, Z93, Z99
Previous medicine use		

Covariate	Data source	Codes
Recent opioid exposure<br>(Dichotomous: 1 = yes, 0 = no)	PBS	Between (initiation - 90) and (initiation - 1):<br>Record of dispensing with ATC N02AA, N02AA01, N02AA03, N02AA05 (except item codes for controlled-release oxycodone), N02AA59, N02AB02, N02AB03, N02AC, N02AC02, N02AC04, N02AE01, N02AJ06, N02AJ07, N02AX02
Falls-risk medicines (FRIDs)[15]<br>(Dichotomous: 1 = yes, 0 = no)	PBS	Between (initiation - 30) and (initiation - 1):<br>Record of dispensing with ATC N05A (excluding N05AN), N05B, N05C, N06A, C01D, C02, C03, C07, C08, C09, G04CA, N04B
Gabapentinoids<br>(Dichotomous: 1 = yes, 0 = no)	PBS	Between (initiation - 30) and (initiation - 1):<br>Record of dispensing with ATC N03AX12, N03AX16, N02BG
Anticholinergic medicines<br>(Dichotomous: 1 = yes, 0 = no)	PBS	Between (initiation - 30) and (initiation - 1):<br>Record of dispensing with ATC N04A
Statins<br>(Dichotomous: 1 = yes, 0 = no)	PBS	Between (initiation - 30) and (initiation - 1):<br>Record of dispensing with ATC C10

APDC: Admitted Patient Data Collection; ATC: Anatomical Therapeutic Chemical classification; EDDC: Emergency Department Data Collection; ICD-10-AM: International Classification of Diseases, 10th Revision, Australian Modification; NSW: New South Wales; PBS: Pharmaceutical Benefits Scheme.

REFERENCES

1. Socio-Economic Indexes for Areas (SEIFA), Australia: Australian Bureau of Statistics; [updated 27 April 2023 accessed: 25 September 2025]. Available from: https://www.abs.gov.au/statistics/people/people-and-communities/socio-economic-indexes-areas-seifa-australia/latest-release.
2. Remoteness Structure: Australian Bureau of Statistics; [updated 20 July 2021 accessed: 25 September 2025]. Available from: https://www.abs.gov.au/statistics/standards/australian-statistical-geography-standard-asgs-edition-3/jul2021-jun2026/remoteness-structure.
3. Remoteness Areas: Australian Bureau of Statistics; [updated 21 March 2023 accessed: 25 September 2025]. Available from: https://www.abs.gov.au/statistics/standards/australian-statistical-geography-standard-asgs-edition-3/jul2021-jun2026/remoteness-structure/remoteness-areas.
4. Gilbert T, Neuburger J, Kraindler J, Keeble E, Smith P, Ariti C, et al. Development and validation of a Hospital Frailty Risk Score focusing on older people in acute care settings using electronic hospital records: an observational study. Lancet. 2018;391(10132):1775-82. Epub 20180426. doi: 10.1016/s0140-6736(18)30668-8. PubMed PMID: 29706364; PubMed Central PMCID: PMCPMC5946808.
5. Street A, Maynou L, Gilbert T, Stone T, Mason S, Conroy S. The use of linked routine data to optimise calculation of the Hospital Frailty Risk Score on the basis of previous hospital admissions: a retrospective observational cohort study. Lancet Healthy Longev. 2021;2(3):e154-e62. doi: 10.1016/s2666-7568(21)00004-0. PubMed PMID: 33733245; PubMed Central PMCID: PMCPMC7934406.
6. Gillies MB, Chidwick K, Bharat C, Camacho X, Currow D, Gisev N, et al. Long-term prescribed opioid use after hospitalization or emergency department presentation among opioid naïve adults (2014-2020)-A population-based descriptive cohort study. Br J Clin Pharmacol. 2024. Epub 20240527. doi: 10.1111/bcp.16093. PubMed PMID: 38803009.
7. Gong J, Merry AF, Beyene KA, Campbell D, Frampton C, Jones P, et al. Persistent opioid use and opioid-related harm after hospital admissions for surgery and trauma in New Zealand: a population-based cohort study. BMJ Open. 2021;11(1):e044493. Epub 20210119. doi: 10.1136/bmjopen-2020-044493. PubMed PMID: 33468530; PubMed Central PMCID: PMCPMC7817825.
8. Seppala LJ, van de Glind EMM, Daams JG, Ploegmakers KJ, de Vries M, Wermelink A, et al. Fall-Risk-Increasing Drugs: A Systematic Review and Meta-analysis: III. Others. J Am Med Dir Assoc. 2018;19(4):372.e1-.e8. Epub 20180302. doi: 10.1016/j.jamda.2017.12.099. PubMed PMID: 29402646.
9. de Vries M, Seppala LJ, Daams JG, van de Glind EMM, Masud T, van der Velde N. Fall-Risk-Increasing Drugs: A Systematic Review and Meta-Analysis: I. Cardiovascular Drugs. J Am Med Dir Assoc. 2018;19(4):371.e1-.e9. Epub 20180212. doi: 10.1016/j.jamda.2017.12.013. PubMed PMID: 29396189.
10. Camacho X, Zoega H, Gomes T, Schaffer AL, Henry D, Pearson SA, et al. The association between psychostimulant use in pregnancy and adverse maternal and neonatal outcomes: results from a distributed analysis in two similar jurisdictions. Int J Epidemiol. 2023;52(1):190-202. doi: 10.1093/ije/dyac180. PubMed PMID: 36135973; PubMed Central PMCID: PMCPMC9908060.

11. Lin J, Pearson SA, Greenfield JR, Park KH, Havard A, Brieger D, et al. Trends in use of sodium-glucose co-transporter 2 inhibitors (SGLT2i) and glucagon-like peptide-1 receptor agonists (GLP-1RA) in Australia in the era of increased evidence of their cardiovascular benefits (2014-2022). Eur J Clin Pharmacol. 2023;79(9):1239-48. Epub 20230714. doi: 10.1007/s00228-023-03539-8. PubMed PMID: 37449993; PubMed Central PMCID: PMCPMC10427543.
12. Australian Institute of Health and Welfare. Chronic musculoskeletal conditions [Internet]. Canberra: 2024. Available from: https://www.aihw.gov.au/reports/chronic-musculoskeletal-conditions/musculoskeletal-conditions/contents/about
13. Youens D, Katzenellenbogen J, Srinivasa Ragavan R, Sodhi-Berry N, Carson J, Zemedikun D, et al. Differing Definitions of First-Ever Stroke Influence Incidence Estimates More than Trends: A Study Using Linked Administrative Data. Neuroepidemiology. 2023;57(6):423-32. Epub 20230926. doi: 10.1159/000534242. PubMed PMID: 37751719.
14. Gisev N, Pearson SA, Dobbins T, Buizen L, Murphy T, Wilson A, et al. Cohort profile: POPPY II - a population-based cohort examining the patterns and outcomes of prescription opioid use in New South Wales, Australia. BMJ Open. 2023;13(5):e068310. Epub 20230517. doi: 10.1136/bmjopen-2022-068310. PubMed PMID: 37197812; PubMed Central PMCID: PMCPMC10193079.
15. Hopkins RE, Bharat C, Buizen L, Close J, Ivers R, Draper B, et al. Age-Related Risk of Serious Fall Events and Opioid Analgesic Use. JAMA Intern Med. 2024;184(4):394-401. doi: 10.1001/jamainternmed.2023.8154. PubMed PMID: 38373005; PubMed Central PMCID: PMCPMC10877504.
