# Supplementary material for: Short-term risk of falls among initiators of controlled-release tapentadol versus oxycodone in New South Wales between 2014 and 2020: A population-based retrospective cohort study
Source: PLoS Med. 2026 Aug 11;23(8):e1004950. doi: 10.1371/journal.pmed.1004950 (PMC13460682; doi:10.1371/journal.pmed.1004950)
Supplement: Supplementary file 6 [file pmed.1004950.s006.pdf]

S5 Table. Baseline characteristics of tapentadol (SR) and oxycodone (CR) initiators aged 65 years and older, 1 September 2014 - 3 December 2020, before and after propensity score matching

Characteristic	Unmatched			Matched		
	Tapentadol SR N = 46,241	Oxycodone CR N = 199,754	SD	Tapentadol SR N = 41,546	Oxycodone CR N = 41,546	SD
Year of initiation*						
2014	1,284 (2.8%)	12,795 (6.4%)	.17	1,212 (2.9%)	1,212 (2.9%)	.00
2015	4,426 (9.6%)	38,660 (19.4%)	.28	4,160 (10.0%)	4,160 (10.0%)	.00
2016	5,163 (11.2%)	39,484 (19.8%)	.24	4,894 (11.8%)	4,894 (11.8%)	.00
2017	7,357 (15.9%)	37,569 (18.8%)	.08	6,921 (16.7%)	6,921 (16.7%)	.00
2018	8,622 (18.7%)	30,539 (15.3%)	.09	7,999 (19.3%)	7,999 (19.3%)	.00
2019	10,819 (23.4%)	24,517 (12.3%)	.29	9,566 (23.0%)	9,566 (23.0%)	.00
2020	8,570 (18.5%)	16,190 (8.1%)	.31	6,794 (16.4%)	6,794 (16.4%)	.00
Sex						
Female	27,256 (58.9%)	108,926 (54.5%)	.09	24,289 (58.5%)	23,722 (57.1%)	.03
Male	18,985 (41.1%)	90,828 (45.5%)		17,257 (41.5%)	17,824 (42.9%)	
Age group (years)						
65-84	40,395 (87.4%)	163,146 (81.7%)	.16	36,794 (88.6%)	36,794 (88.6%)	.00
85 plus	5,846 (12.6%)	36,608 (18.3%)	.16	4,752 (11.4%)	4,752 (11.4%)	.00
Remoteness Area						
Major cities	29,699 (64.2%)	126,846 (63.5%)	.02	26,620 (64.1%)	26,626 (64.1%)	.00
Inner regional	13,084 (28.3%)	54,875 (27.5%)	.02	11,796 (28.4%)	11,609 (27.9%)	.01
Outer regional	3,072 (6.6%)	15,963 (8.0%)	.05	2,786 (6.7%)	2,924 (7.0%)	.01
Remote	160 (0.4%)	996 (0.5%)	.02	139 (0.3%)	165 (0.4%)	.01
Very remote	18 (0.0%)	99 (0.1%)	.01	16 (0.0%)	21 (0.1%)	.01
Missing	208 (0.5%)	975 (0.5%)	.01	189 (0.5%)	201 (0.5%)	.00
Decile of relative disadvantage						
1 (most disadvantage)	2,986 (6.5%)	14,235 (7.1%)	.03	2,695 (6.5%)	2,910 (7.0%)	.02
2	4,282 (9.3%)	18,828 (9.4%)	.01	3,847 (9.3%)	3,932 (9.5%)	.01
3	3,751 (8.1%)	17,082 (8.6%)	.02	3,428 (8.3%)	3,469 (8.4%)	.00

Characteristic	Unmatched			Matched		
	Tapentadol SR N = 46,241	Oxycodone CR N = 199,754	SD	Tapentadol SR N = 41,546	Oxycodone CR N = 41,546	SD
4	5,568 (12.0%)	25,638 (12.8%)	.02	5,070 (12.2%)	5,074 (12.2%)	.00
5	6,117 (13.2%)	23,761 (11.9%)	.04	5,286 (12.7%)	5,323 (12.8%)	.00
6	5,061 (10.9%)	21,853 (10.9%)	.00	4,585 (11.0%)	4,594 (11.1%)	.00
7	4,371 (9.5%)	18,879 (9.5%)	.00	3,945 (9.5%)	4,051 (9.8%)	.01
8	4,034 (8.7%)	17,967 (9.0%)	.01	3,635 (8.8%)	3,661 (8.8%)	.00
9	5,467 (11.8%)	21,974 (11.0%)	.03	4,911 (11.8%)	4,616 (11.1%)	.02
10 (least disadvantage)	4,386 (9.5%)	18,533 (9.3%)	.01	3,946 (9.5%)	3,703 (8.9%)	.02
Missing	218 (0.5%)	1,004 (0.5%)	.00	198 (0.5%)	213 (0.5%)	.01
Exposure to non-study opioids (prior 90 days)						
None	29,940 (64.8%)	117,517 (58.8%)	.12	27,056 (65.1%)	27,056 (65.1%)	.00
Yes	16,301 (35.3%)	82,237 (41.2%)		14,490 (34.9%)	14,490 (34.9%)	
Comorbidities						
Cancer	5,537 (12.0%)	39,155 (19.6%)	.21	4,596 (11.1%)	4,864 (11.7%)	.02
Substance use disorder	1,184 (2.6%)	5,977 (3.0%)	.03	1,032 (2.5%)	1,214 (2.9%)	.03
Prior overdose	47 (0.1%)	207 (0.1%)	.00	39 (0.1%)	34 (0.1%)	.00
Depression	14,341 (31.0%)	60,098 (30.1%)	.02	12,440 (29.9%)	13,102 (31.5%)	.03
Anxiety	5,071 (11.0%)	22,073 (11.1%)	.00	4,481 (10.8%)	4,509 (10.9%)	.00
Hypertension	15,662 (33.9%)	68,719 (34.4%)	.01	13,674 (32.9%)	14,402 (34.7%)	.04
Atrial fibrillation or flutter	1,747 (3.8%)	10,661 (5.3%)	.07	1,501 (3.6%)	1,487 (3.6%)	.00
Diabetes	9,843 (21.3%)	44,546 (22.3%)	.02	8,830 (21.3%)	8,968 (21.6%)	.01
Congestive heart failure	10,930 (23.6%)	52,451 (26.3%)	.06	9,635 (23.2%)	9,741 (23.5%)	.01
Thyroid disease	5,539 (12.0%)	22,403 (11.2%)	.02	4,881 (11.8%)	4,792 (11.5%)	.01
Postural hypotension	414 (0.9%)	2,352 (1.2%)	.03	344 (0.8%)	388 (0.9%)	.01
Osteoporosis	10,284 (22.2%)	51,043 (25.6%)	.08	8,917 (21.5%)	8,859 (21.3%)	.00
Stroke	16,312 (35.3%)	79,231 (39.7%)	.09	14,658 (35.3%)	13,774 (33.2%)	.04
Renal impairment	1,269 (2.7%)	8,614 (4.3%)	.09	1,071 (2.6%)	1,099 (2.7%)	.00
Hepatic impairment	379 (0.8%)	3,164 (1.6%)	.07	300 (0.7%)	253 (0.6%)	.01

Characteristic	Unmatched			Matched		
	Tapentadol SR N = 46,241	Oxycodone CR N = 199,754	SD	Tapentadol SR N = 41,546	Oxycodone CR N = 41,546	SD
Surgery (prior 30 days)	11,469 (24.8%)	58,653 (29.4%)	.10	10,149 (24.4%)	9,894 (23.8%)	.01
Falls-risk medicines (FRIDs; prior 30 days)						
Yes	30,703 (66.4%)	132,032 (66.1%)	.01	27,447 (66.1%)	27,288 (65.7%)	.01
Number of FRIDs:						
1	15,621 (50.9%)	65,702 (49.8%)	.02	14,158 (51.6%)	13,714 (50.3%)	.03
2	9,504 (31.0%)	41,090 (31.1%)	.00	8,429 (30.7%)	8,530 (31.3%)	.01
3	4,008 (13.1%)	17,833 (13.5%)	.01	3,512 (12.8%)	3,601 (13.2%)	.01
4	1,204 (3.9%)	5,635 (4.3%)	.02	1,037 (3.8%)	1,106 (4.1%)	.01
5+	366 (1.2%)	1,772 (1.3%)	.01	311 (1.1%)	337 (1.2%)	.01
Type of FRID						
Medicines associated with high risk of falls						
Antipsychotics (excluding lithium)	539 (1.8%)	3,949 (3.0%)	.08	478 (1.7%)	741 (2.7%)	.07
Anxiolytics	1,342 (4.4%)	6,688 (5.1%)	.03	1,185 (4.3%)	1,334 (4.9%)	.03
Hypnotics and sedatives	1,715 (5.6%)	8,993 (6.8%)	.05	1,473 (5.4%)	1,561 (5.7%)	.02
Antidepressants	8,395 (27.3%)	36,337 (27.5%)	.00	7,248 (26.4%)	7,949 (29.1%)	.06
Medicines that cause orthostatism/hypotension						
Vasodilators used in cardiac disease (e.g. nitrates)	1,305 (4.3%)	7,157 (5.4%)	.05	1,145 (4.2%)	1,156 (4.2%)	.00
Antihypertensives	1,546 (5.0%)	5,856 (4.4%)	.03	1,366 (5.0%)	1,268 (4.7%)	.02
Diuretics	2,773 (9.0%)	14,647 (11.1%)	.07	2,396 (8.7%)	2,492 (9.1%)	.01
Beta blockers	5,909 (19.3%)	26,537 (20.1%)	.02	5,269 (19.2%)	5,177 (19.0%)	.01
Calcium channel blockers	6,616 (21.6%)	28,879 (21.9%)	.01	5,895 (21.5%)	5,869 (21.5%)	.00
Renin-angiotensin acting agents	21,205 (69.1%)	85,846 (65.0%)	.09	19,062 (69.5%)	18,473 (67.7%)	.04
Alpha adrenoreceptor blockers	1,058 (3.5%)	4,409 (3.3%)	.01	943 (3.4%)	924 (3.4%)	.00
Dopaminergic agents	969 (3.2%)	3,845 (2.9%)	.01	854 (3.1%)	799 (2.9%)	.01
Other medicines associated with falls (prior 30 days)						
Gabapentinoids	5,923 (12.8%)	19,045 (9.5%)	.10	4,406 (10.6%)	4,203 (10.1%)	.02
Anticholinergic medicines	14 (0.0%)	75 (0.0%)	.00	12 (0.0%)	10 (0.0%)	.00

Characteristic	Unmatched			Matched		
	**Tapentadol SR N = 46,241**	**Oxycodone CR N = 199,754**	**SD**	**Tapentadol SR N = 41,546**	**Oxycodone CR N = 41,546**	**SD**
Statins	19,430 (42.0%)	78,596 (39.4%)	.05	17,393 (41.9%)	17,064 (41.1%)	.02
Hospital frailty risk score						
Low: <5	38,109 (82.4%)	150,135 (75.2%)	.18	34,536 (83.1%)	34,251 (82.4%)	.02
Medium: 5-15	5,792 (12.5%)	33,224 (16.6%)	.12	5,101 (12.3%)	5,140 (12.4%)	.00
High: >15	2,340 (5.1%)	16,395 (8.2%)	.13	1,909 (4.6%)	2,155 (5.2%)	.03
History of falls						
Falls in the past 2 years to 60 days	2,792 (6.0%)	16,826 (8.4%)	.09	2,397 (5.8%)	2,657 (6.4%)	.03

Note: cohort matched on propensity score, year and month of initiation, age, and exposure to non-study opioids in the prior 90 days

* study period: 01 September 2014 – 03 December 2020

SR = sustained release; CR = controlled release; SD = absolute standardised difference
