# Supplementary material for: Short-term risk of falls among initiators of controlled-release tapentadol versus oxycodone in New South Wales between 2014 and 2020: A population-based retrospective cohort study
Source: PLoS Med. 2026 Aug 11;23(8):e1004950. doi: 10.1371/journal.pmed.1004950 (PMC13460682; doi:10.1371/journal.pmed.1004950)
Supplement: Supplementary file 7 [file pmed.1004950.s007.pdf]

S6 Table. Baseline characteristics of tapentadol (SR) and oxycodone (CR) initiators aged 80 years and older, 1 September 2014 - 3 December 2020, before and after propensity score matching

Characteristic	Unmatched			Matched		
	Tapentadol SR N = 12,563	Oxycodone CR N = 67,439	SD	Tapentadol SR N = 10,783	Oxycodone CR N = 10,783	SD
Year of initiation*						
2014	393 (3.1%)	4,225 (6.3%)	.15	360 (3.3%)	360 (3.3%)	.00
2015	1,362 (10.8%)	12,959 (19.2%)	.24	1,247 (11.6%)	1,247 (11.6%)	.00
2016	1,518 (12.1%)	13,106 (19.4%)	.20	1,390 (12.9%)	1,390 (12.9%)	.00
2017	2,040 (16.2%)	12,383 (18.4%)	.06	1,842 (17.1%)	1,842 (17.1%)	.00
2018	2,260 (18.0%)	10,316 (15.3%)	.07	2,000 (18.6%)	2,000 (18.6%)	.00
2019	2,663 (21.2%)	8,471 (12.6%)	.23	2,224 (20.6%)	2,224 (20.6%)	.00
2020	2,327 (18.5%)	5,979 (8.9%)	.28	1,720 (16.0%)	1,720 (16.0%)	.00
Sex						
Female	7,905 (62.92%)	40,828 (60.5%)	.05	6,759 (62.7%)	6,786 (62.9%)	.01
Male	4,658 (37.08%)	26,611 (39.5%)		4,024 (37.3%)	3,997 (37.1%)	
Age group (years)						
65-84	6,717 (53.5%)	30,831 (45.7%)	.16	6,031 (55.9%)	6,031 (55.9%)	.00
85 plus	5,846 (46.5%)	36,608 (54.3%)	.16	4,752 (44.1%)	4,752 (44.1%)	
Remoteness Area						
Major cities	8,396 (66.8%)	43,736 (64.9%)	.04	7,170 (66.5%)	7,064 (65.5%)	.02
Inner regional	3,354 (26.7%)	18,049 (26.8%)	.00	2,906 (27.0%)	2,923 (27.1%)	.00
Outer regional	741 (5.9%)	5,032 (7.5%)	.06	645 (6.0%)	713 (6.6%)	.03
Remote / Very Remote†	40 (0.3%)	328 (0.5%)	.03	34 (0.3%)	42 (0.4%)	.01
Missing	32 (0.3%)	294 (0.4%)	.03	28 (0.3%)	41 (0.4%)	.02
Decile of relative disadvantage						
1 (most disadvantage)	783 (6.2%)	4,524 (6.7%)	.02	676 (6.3%)	742 (6.9%)	.02
2	1,168 (9.3%)	6,282 (9.3%)	.00	1,000 (9.3%)	1,039 (9.6%)	.01
3	922 (7.3%)	5,458 (8.1%)	.03	817 (7.6%)	845 (7.8%)	.01
4	1,381 (11.0%)	8,250 (12.2%)	.04	1,199 (11.1%)	1,257 (11.7%)	.02

Characteristic	Unmatched			Matched		
	Tapentadol SR N = 12,563	Oxycodone CR N = 67,439	SD	Tapentadol SR N = 10,783	Oxycodone CR N = 10,783	SD
5	1,761 (14.0%)	8,533 (12.7%)	.04	1,471 (13.6%)	1,415 (13.1%)	.02
6	1,376 (11.0%)	7,355 (10.9%)	.00	1,203 (11.2%)	1,152 (10.7%)	.02
7	1,158 (9.2%)	6,158 (9.1%)	.00	998 (9.3%)	1,013 (9.4%)	.00
8	1,143 (9.1%)	6,237 (9.3%)	.01	987 (9.2%)	1,003 (9.3%)	.01
9	1,512 (12.0%)	7,660 (11.4%)	.02	1,285 (11.9%)	1,266 (11.7%)	.01
10 (least disadvantage)	1,324 (10.5%)	6,680 (9.9%)	.02	1,116 (10.4%)	1,008 (9.4%)	.03
Missing	35 (0.3%)	302 (0.5%)	.03	31 (0.3%)	43 (0.4%)	.02
Exposure to non-study opioids (prior 90 days)						
None	7,802 (62.1%)	38,428 (57.0%)	.10	6,812 (63.2%)	6,812 (63.2%)	.00
Yes	4,761 (37.9%)	29,011 (43.0%)		3,971 (36.8%)	3,971 (36.8%)	
Comorbidities						
Cancer	1,621 (12.9%)	12,707 (18.8%)	.16	1,233 (11.4%)	1,256 (11.7%)	.01
Substance use disorder	144 (1.2%)	1,020 (1.5%)	.03	116 (1.1%)	167 (1.6%)	.04
Prior overdose	19 (0.2%)	76 (0.1%)	.01	16 (0.2%)	15 (0.1%)	.00
Depression	3,928 (31.3%)	21,341 (31.6%)	.01	3,274 (30.4%)	3,572 (33.1%)	.06
Anxiety	1,334 (10.6%)	7,295 (10.8%)	.01	1,138 (10.6%)	1,150 (10.7%)	.00
Hypertension	4,763 (37.9%)	25,732 (38.2%)	.01	3,981 (36.9%)	4,194 (38.9%)	.04
Atrial fibrillation or flutter	746 (5.9%)	5,182 (7.7%)	.07	610 (5.7%)	597 (5.5%)	.01
Diabetes	2,470 (19.7%)	13,769 (20.4%)	.02	2,137 (19.8%)	2,209 (20.5%)	.02
Congestive heart failure	4,286 (34.1%)	24,693 (36.6%)	.05	3,616 (33.5%)	3,674 (34.1%)	.01
Thyroid disease	1,715 (13.7%)	8,748 (13.0%)	.02	1,461 (13.6%)	1,474 (13.7%)	.00
Postural hypotension	218 (1.7%)	1,334 (2.0%)	.02	180 (1.7%)	177 (1.6%)	.00
Osteoporosis	3,999 (31.8%)	22,230 (33.0%)	.02	3,338 (31.0%)	3,305 (30.7%)	.01
Stroke	5,817 (46.3%)	32,893 (48.8%)	.05	4,991 (46.3%)	4,699 (43.6%)	.05
Renal impairment	593 (4.7%)	4,620 (6.9%)	.09	475 (4.4%)	491 (4.6%)	.01
Hepatic impairment	112 (0.9%)	1,114 (1.7%)	.07	90 (0.8%)	69 (0.6%)	.02
Surgery (prior 30 days)	2,694 (21.4%)	16,790 (24.9%)	.08	2,249 (20.9%)	2,171 (20.1%)	.02

Characteristic	Unmatched			Matched		
	Tapentadol SR N = 12,563	Oxycodone CR N = 67,439	SD	Tapentadol SR N = 10,783	Oxycodone CR N = 10,783	SD
Falls-risk medicines (FRIDs; prior 30 days)						
Yes	9,216 (73.4%)	48,761 (72.3%)	.02	7,889 (73.2%)	7,863 (72.9%)	.01
Number of FRIDs:						
1	4,228 (45.9%)	21,897 (44.9%)	.02	3,681 (46.7%)	3,406 (43.3%)	.07
2	2,978 (32.3%)	15,778 (32.4%)	.00	2,508 (31.8%)	2,668 (33.9%)	.05
3	1,426 (15.5%)	7,584 (15.6%)	.00	1,207 (15.3%)	1,240 (15.8%)	.01
4	455 (4.9%)	2,645 (5.4%)	.02	383 (4.9%)	412 (5.2%)	.02
5+	129 (1.4%)	857 (1.8%)	.03	110 (1.4%)	137 (1.7%)	.03
Type of FRID						
Medicines associated with high risk of falls						
Antipsychotics (excluding lithium)	180 (2.0%)	1,910 (3.9%)	.12	154 (2.0%)	266 (3.4%)	.09
Anxiolytics	382 (4.1%)	2,293 (4.7%)	.03	325 (4.1%)	342 (4.4%)	.01
Hypnotics and sedatives	659 (7.2%)	4,328 (8.9%)	.06	542 (6.9%)	614 (7.8%)	.04
Antidepressants	2,230 (24.2%)	12,963 (26.6%)	.05	1,848 (23.4%)	2,156 (27.4%)	.09
Medicines that cause orthostatism/hypotension						
Vasodilators used in cardiac disease (e.g. nitrates)	659 (7.2%)	4,087 (8.4%)	.05	553 (7.0%)	556 (7.1%)	.00
Antihypertensives	424 (4.6%)	1,990 (4.1%)	.03	358 (4.5%)	323 (4.1%)	.02
Diuretics	1,232 (13.4%)	7,852 (16.1%)	.08	1,016 (12.9%)	1,092 (13.9%)	.03
Beta blockers	2,110 (22.9%)	11,210 (23.0%)	.00	1,804 (22.9%)	1,805 (23.0%)	.00
Calcium channel blockers	2,329 (25.3%)	11,938 (24.5%)	.02	1,995 (25.3%)	2,041 (26.0%)	.02
Renin-angiotensin acting agents	6,010 (65.2%)	29,035 (59.6%)	.12	5,200 (65.9%)	4,998 (63.6%)	.05
Alpha adrenoreceptor blockers	405 (4.4%)	1,990 (4.1%)	.02	341 (4.3%)	352 (4.5%)	.01
Dopaminergic agents	332 (3.6%)	1,660 (3.4%)	.01	281 (3.6%)	274 (3.5%)	.00
Other medicines associated with falls (prior 30 days)						
Gabapentinoids	1,625 (12.9%)	6,358 (9.4%)	.11	1,119 (10.4%)	1,010 (9.4%)	.03
Anticholinergic medicines	6 (0.1%)	13 (0.0%)	.02	n.p.	n.p.	n.p.
Statins	5,526 (44.0%)	26,226 (38.9%)	.10	4,763 (44.2%)	4,559 (42.3%)	.04
Hospital frailty risk score						

Characteristic	Unmatched			Matched		
	Tapentadol SR N = 12,563	Oxycodone CR N = 67,439	SD	Tapentadol SR N = 10,783	Oxycodone CR N = 10,783	SD
Low: <5	8,989 (71.6%)	42,406 (62.9%)	.19	7,834 (72.7%)	7,689 (71.3%)	.03
Medium: 5-15	2,296 (18.3%)	14,786 (21.9%)	.09	1,958 (18.2%)	1,899 (17.6%)	.01
High: >15	1,278 (10.2%)	10,247 (15.2%)	.15	991 (9.2%)	1,195 (11.1%)	.06
History of falls						
Falls in the past 2 years to 60 days	1,468 (11.7%)	10,520 (15.6%)	.11	1,206 (11.2%)	1,389 (12.9%)	.05

Note: cohort matched on propensity score, year and month of initiation, age, and exposure to non-study opioids in the prior 90 days

* study period: 01 September 2014 – 03 December 2020

† categories combined to avoid residual disclosure of small cells

SR = sustained release; CR = controlled release; SD = absolute standardised difference; n.p. = value not presented to avoid residual disclosure of small cells
