# Supplementary material for: Short-term risk of falls among initiators of controlled-release tapentadol versus oxycodone in New South Wales between 2014 and 2020: A population-based retrospective cohort study
Source: PLoS Med. 2026 Aug 11;23(8):e1004950. doi: 10.1371/journal.pmed.1004950 (PMC13460682; doi:10.1371/journal.pmed.1004950)
Supplement: Supplementary file 8 [file pmed.1004950.s008.pdf]

S7 Table. Unadjusted relative risks of falls among people initiating tapentadol (SR) compared to oxycodone (CR), overall and by recent exposure to non-study opioids, for the total study population, people aged 65+, and people aged 80+

Outcome	Exposure	Overall			No recent exposure			Exposure in prior 90 days		
		No. exposed	No. events	RR (95% CI)	No. exposed	No. events	RR (95% CI)	No. exposed	No. events	RR (95% CI)
Overall population										
7 days post-initiation	Oxycodone CR	419,732	1,396	0.55 (0.47-0.64)	250,030	748	0.61 (0.50-0.73)	169,702	648	0.49 (0.38-0.62)
	Tapentadol SR	103,924	190		65,715	119		38,209	71	
14 days post-initiation	Oxycodone CR	419,732	2,108	0.58 (0.52-0.66)	250,030	1,147	0.60 (0.52-0.71)	169,702	961	0.56 (0.47-0.68)
	Tapentadol SR	103,924	304		65,715	182		38,209	122	
28 days post-initiation	Oxycodone CR	419,732	3,007	0.62 (0.56-0.68)	250,030	1,618	0.63 (0.56-0.72)	169,702	1,389	0.61 (0.52-0.71)
	Tapentadol SR	103,924	460		65,715	270		38,209	190	
People aged 65 years and older										
7 days post-initiation	Oxycodone CR	199,754	1,128	0.58 (0.49-0.69)	117,517	609	0.61 (0.49-0.76)	82,237	519	0.55 (0.42-0.73)
	Tapentadol SR	46,241	152		29,940	95		16,301	57	
14 days post-initiation	Oxycodone CR	199,754	1,723	0.60 (0.53-0.69)	117,517	942	0.60 (0.51-0.72)	82,237	781	0.61 (0.50-0.76)
	Tapentadol SR	46,241	240		29,940	145		16,301	95	
28 days post-initiation	Oxycodone CR	199,754	2,480	0.63 (0.57-0.71)	117,517	1,354	0.63 (0.55-0.73)	82,237	1,126	0.66 (0.56-0.78)
	Tapentadol SR	46,241	364		29,940	217		16,301	147	
People aged 80 years and older										
7 days post-initiation	Oxycodone CR	67,439	701	0.71 (0.57-0.88)	38,428	403	0.71 (0.54-0.93)	29,011	298	0.72 (0.50-1.01)
	Tapentadol SR	12,563	93		7,802	58		4,761	35	
14 days post-initiation	Oxycodone CR	67,439	1,091	0.73 (0.62-0.87)	38,428	631	0.73 (0.58-0.90)	29,011	460	0.74 (0.56-0.98)
	Tapentadol SR	12,563	149		7,802	93		4,761	56	
28 days post-initiation	Oxycodone CR	67,439	1,606	0.75 (0.65-0.86)	38,428	922	0.74 (0.62-0.89)	29,011	684	0.76 (0.61-0.95)
	Tapentadol SR	12,563	224		7,802	139		4,761	85	

SR = sustained release; CR = controlled release; RR = relative risk; CI = confidence interval
