# Supplementary material for: Short-term risk of falls among initiators of controlled-release tapentadol versus oxycodone in New South Wales between 2014 and 2020: A population-based retrospective cohort study
Source: PLoS Med. 2026 Aug 11;23(8):e1004950. doi: 10.1371/journal.pmed.1004950 (PMC13460682; doi:10.1371/journal.pmed.1004950)
Supplement: Supplementary file 9 [file pmed.1004950.s009.pdf]

S8 Table. Baseline characteristics of tapentadol (SR) and oxycodone (CR) initiators with only study opioids dispensed on initiation date, 1 September 2014 - 3 December 2020, before and after propensity score matching

Characteristic	Unmatched			Matched		
	Tapentadol SR N = 87,542	Oxycodone CR N = 281,697	SD	Tapentadol SR N = 86,804	Oxycodone CR N = 86,804	SD
Year of initiation*						
2014	2,556 (2.9%)	17,305 (6.1%)	.16	2,554 (2.9%)	2,554 (2.9%)	.00
2015	8,602 (9.8%)	53,326 (18.9%)	.26	8,589 (9.9%)	8,589 (9.9%)	.00
2016	9,826 (11.2%)	55,837 (19.8%)	.24	9,813 (11.3%)	9,813 (11.3%)	.00
2017	14,470 (16.5%)	54,180 (19.2%)	.07	14,454 (16.7%)	14,454 (16.7%)	.00
2018	16,177 (18.5%)	44,268 (15.7%)	.07	16,138 (18.6%)	16,138 (18.6%)	.00
2019	20,057 (22.9%)	34,603 (12.3%)	.28	19,932 (23.0%)	19,932 (23.0%)	.00
2020	15,854 (18.1%)	22,178 (7.9%)	.31	15,324 (17.7%)	15,324 (17.7%)	.00
Sex						
Female	51,214 (58.5%)	155,652 (55.3%)	.07	50,702 (58.4%)	50,770 (58.5%)	.00
Male	36,328 (41.5%)	126,045 (44.7%)		36,102 (41.6%)	36,034 (41.5%)	
Age group (years)						
18-44	17,022 (19.4%)	53,811 (19.1%)	.01	16,954 (19.5%)	17,234 (19.9%)	.01
45-64	30,435 (34.8%)	85,885 (30.5%)	.09	30,092 (34.7%)	29,937 (34.5%)	.00
65-84	34,711 (39.7%)	111,868 (39.7%)	.00	34,414 (39.7%)	34,123 (39.3%)	.01
85 plus	5,374 (6.1%)	30,133 (10.7%)	.16	5,344 (6.2%)	5,510 (6.4%)	.01
Remoteness Area						
Major cities	57,890 (66.1%)	184,394 (65.5%)	.01	57,422 (66.2%)	57,580 (66.3%)	.00
Inner regional	23,192 (26.5%)	72,110 (25.6%)	.02	22,950 (26.4%)	22,819 (26.3%)	.00
Outer regional	5,690 (6.5%)	21,958 (7.8%)	.05	5,665 (6.5%)	5,623 (6.5%)	.00
Remote	354 (0.4%)	1,752 (0.6%)	.03	353 (0.4%)	373 (0.4%)	.00
Very remote	50 (0.1%)	195 (0.1%)	.00	49 (0.1%)	54 (0.1%)	.00
Missing	366 (0.4%)	1,288 (0.5%)	.01	365 (0.4%)	355 (0.4%)	.00
Decile of relative disadvantage						
1 (most disadvantage)	6,527 (7.5%)	22,747 (8.1%)	.02	6,495 (7.5%)	6,255 (7.2%)	.01

Characteristic	Unmatched			Matched		
	Tapentadol SR N = 87,542	Oxycodone CR N = 281,697	SD	Tapentadol SR N = 86,804	Oxycodone CR N = 86,804	SD
2	8,073 (9.2%)	26,290 (9.3%)	.00	8,006 (9.2%)	8,067 (9.3%)	.00
3	7,101 (8.1%)	23,665 (8.4%)	.01	7,059 (8.1%)	6,917 (8.0%)	.01
4	10,366 (11.8%)	35,127 (12.5%)	.02	10,294 (11.9%)	10,373 (12.0%)	.00
5	11,503 (13.1%)	31,621 (11.2%)	.06	11,312 (13.0%)	11,268 (13.0%)	.00
6	9,929 (11.3%)	31,952 (11.3%)	.00	9,850 (11.4%)	9,828 (11.3%)	.00
7	8,833 (10.1%)	28,277 (10.0%)	.00	8,752 (10.1%)	8,907 (10.3%)	.01
8	7,639 (8.7%)	25,039 (8.9%)	.01	7,593 (8.8%)	7,698 (8.9%)	.00
9	9,651 (11.0%)	30,703 (10.9%)	.00	9,580 (11.0%)	9,623 (11.1%)	.00
10 (least disadvantage)	7,533 (8.6%)	24,944 (8.9%)	.01	7,478 (8.6%)	7,500 (8.6%)	.00
Missing	387 (0.4%)	1,332 (0.5%)	.00	385 (0.4%)	368 (0.4%)	.00
Exposure to non-study opioids (prior 90 days)						
None	56,947 (65.1%)	171,359 (60.8%)	.09	56,293 (64.9%)	56,293 (64.9%)	.00
Yes	30,595 (35.0%)	110,338 (39.2%)		30,511 (35.2%)	30,511 (35.2%)	
Comorbidities						
Cancer	7,541 (8.6%)	39,665 (14.1%)	.17	7,506 (8.7%)	7,554 (8.7%)	.00
Substance use disorder	3,868 (4.4%)	12,910 (4.6%)	.01	3,845 (4.4%)	3,860 (4.5%)	.00
Prior overdose	209 (0.2%)	671 (0.2%)	.00	206 (0.2%)	202 (0.2%)	.00
Depression	30,473 (34.8%)	88,373 (31.4%)	.07	30,118 (34.7%)	30,028 (34.6%)	.00
Anxiety	11,731 (13.4%)	36,247 (12.9%)	.02	11,623 (13.4%)	11,367 (13.1%)	.01
Hypertension	18,322 (20.9%)	61,777 (21.9%)	.02	18,073 (20.8%)	19,193 (22.1%)	.03
Atrial fibrillation or flutter	1,843 (2.1%)	9,039 (3.2%)	.07	1,831 (2.1%)	1,847 (2.1%)	.00
Diabetes	13,991 (16.0%)	48,474 (17.2%)	.03	13,879 (16.0%)	14,068 (16.2%)	.01
Congestive heart failure	12,419 (14.2%)	47,942 (17.0%)	.08	12,323 (14.2%)	12,421 (14.3%)	.00
Thyroid disease	7,783 (8.9%)	24,376 (8.7%)	.01	7,695 (8.9%)	7,648 (8.8%)	.00
Postural hypotension	458 (0.5%)	2,011 (0.7%)	.02	452 (0.5%)	478 (0.6%)	.00
Osteoporosis	11,189 (12.8%)	46,096 (16.4%)	.10	11,109 (12.8%)	11,211 (12.9%)	.00
Stroke	18,511 (21.2%)	69,778 (24.8%)	.09	18,382 (21.2%)	17,650 (20.3%)	.02
Renal impairment	1,444 (1.7%)	7,714 (2.7%)	.07	1,427 (1.6%)	1,359 (1.6%)	.01

Characteristic	Unmatched			Matched		
	Tapentadol SR N = 87,542	Oxycodone CR N = 281,697	SD	Tapentadol SR N = 86,804	Oxycodone CR N = 86,804	SD
Hepatic impairment	816 (0.9%)	4,767 (1.7%)	.07	811 (0.9%)	648 (0.8%)	.02
Surgery (prior 30 days)	16,511 (18.9%)	62,729 (22.3%)	.08	16,354 (18.8%)	16,494 (19.0%)	.00
Falls-risk medicines (FRIDs; prior 30 days)						
Yes	45,121 (51.5%)	145,547 (51.7%)	.00	44,681 (51.5%)	44,445 (51.2%)	.01
Number of FRIDs:						
1	25,863 (57.3%)	79,853 (54.9%)	.05	25,616 (57.3%)	24,746 (55.7%)	.03
2	12,712 (28.2%)	42,056 (28.9%)	.02	12,584 (28.2%)	12,816 (28.8%)	.01
3	4,817 (10.7%)	16,830 (11.6%)	.03	4,762 (10.7%)	4,913 (11.1%)	.01
4	1,329 (3.0%)	5,221 (3.6%)	.04	1,321 (3.0%)	1,519 (3.4%)	.03
5+	400 (0.9%)	1,587 (1.1%)	.02	398 (0.9%)	451 (1.0%)	.01
Type of FRID						
Medicines associated with high risk of falls						
Antipsychotics (excluding lithium)	1,649 (3.7%)	6,561 (4.5%)	.04	1,635 (3.7%)	1,937 (4.4%)	.04
Anxiolytics	3,521 (7.8%)	11,917 (8.2%)	.01	3,496 (7.8%)	3,703 (8.3%)	.02
Hypnotics and sedatives	2,377 (5.3%)	9,370 (6.4%)	.05	2,362 (5.3%)	2,401 (5.4%)	.01
Antidepressants	17,230 (38.2%)	51,403 (35.3%)	.06	17,021 (38.1%)	17,752 (39.9%)	.04
Medicines that cause orthostatism/hypotension						
Vasodilators used in cardiac disease (e.g. nitrates)	1,357 (3.0%)	6,241 (4.3%)	.07	1,350 (3.0%)	1,460 (3.3%)	.02
Antihypertensives	1,835 (4.1%)	5,516 (3.8%)	.01	1,810 (4.1%)	1,740 (3.9%)	.01
Diuretics	3,007 (6.7%)	13,072 (9.0%)	.09	2,990 (6.7%)	3,262 (7.3%)	.03
Beta blockers	6,873 (15.2%)	24,736 (17.0%)	.05	6,829 (15.3%)	6,896 (15.5%)	.01
Calcium channel blockers	7,148 (15.8%)	25,234 (17.3%)	.04	7,073 (15.8%)	7,169 (16.1%)	.01
Renin-angiotensin acting agents	25,984 (57.6%)	82,637 (56.8%)	.02	25,721 (57.6%)	25,226 (56.8%)	.02
Alpha adrenoreceptor blockers	1,033 (2.3%)	3,424 (2.4%)	.00	1,025 (2.3%)	935 (2.1%)	.01
Dopaminergic agents	1,121 (2.5%)	3,491 (2.4%)	.01	1,113 (2.5%)	1,065 (2.4%)	.01
Other medicines associated with falls (prior 30 days)						
Gabapentinoids	10,344 (11.8%)	23,314 (8.3%)	.12	10,060 (11.6%)	9,923 (11.4%)	.00

Characteristic	Unmatched			Matched		
	Tapentadol SR N = 87,542	Oxycodone CR N = 281,697	SD	Tapentadol SR N = 86,804	Oxycodone CR N = 86,804	SD
Anticholinergic medicines	26 (0.0%)	133 (0.1%)	.01	26 (0.0%)	17 (0.0%)	.01
Statins	22,948 (26.2%)	73,153 (26.0%)	.01	22,689 (26.1%)	22,701 (26.2%)	.00
Hospital frailty risk score						
Low: <5	77,397 (88.4%)	233,949 (83.1%)	.15	76,715 (88.4%)	76,708 (88.4%)	.00
Medium: 5-15	7,584 (8.7%)	33,076 (11.7%)	.10	7,553 (8.7%)	7,486 (8.6%)	.00
High: >15	2,561 (2.9%)	14,672 (5.2%)	.12	2,536 (2.9%)	2,610 (3.0%)	.01
History of falls						
Falls in the past 2 years to 60 days	3,374 (3.9%)	15,838 (5.6%)	.08	3,347 (3.9%)	3,475 (4.0%)	.01

Note: cohort matched on propensity score, year and month of initiation, and recent exposure to opioids

* study period: 01 September 2014 – 03 December 2020

SR = sustained release; CR = controlled release; SD = absolute standardised difference
