# Supplementary material for: Short-term risk of falls among initiators of controlled-release tapentadol versus oxycodone in New South Wales between 2014 and 2020: A population-based retrospective cohort study
Source: PLoS Med. 2026 Aug 11;23(8):e1004950. doi: 10.1371/journal.pmed.1004950 (PMC13460682; doi:10.1371/journal.pmed.1004950)
Supplement: Supplementary file 10 [file pmed.1004950.s010.pdf]

S9 Table. Relative risks of falls and cataract surgeries among tapentadol (SR) and oxycodone (CR) initiators with only study opioids dispensed on initiation date

Outcome	Exposure	Unmatched			Matched		
		No. exposed	No. events	RR (95% CI)	No. exposed	No. events	Matched RR[1] (95% CI)
Falls resulting in ED presentation, hospitalisation, or death							
7 days post-initiation	Oxycodone CR	281,697	957	0.56 (0.47-0.66)	86,804	263	0.62 (0.51-0.75)
	Tapentadol SR	87,542	166		86,804	163	
14 days post-initiation	Oxycodone CR	281,697	1,485	0.56 (0.49-0.64)	86,804	414	0.62 (0.53-0.72)
	Tapentadol SR	87,542	260		86,804	257	
28 days post-initiation	Oxycodone CR	281,697	2,162	0.59 (0.53-0.65)	86,804	585	0.67 (0.59-0.76)
	Tapentadol SR	87,542	395		86,804	390	
Cataract surgeries (negative control outcome)							
7 days post-initiation	Oxycodone CR	281,697	96	0.97 (0.64-1.47)	86,804	22	1.32 (0.76-2.29)
	Tapentadol SR	87,542	29		86,804	29	
14 days post-initiation	Oxycodone CR	281,697	201	1.12 (0.85-1.47)	86,804	63	1.11 (0.79-1.56)
	Tapentadol SR	87,542	70		86,804	70	
28 days post-initiation	Oxycodone CR	281,697	411	1.10 (0.90-1.33)	86,804	128	1.09 (0.86-1.39)
	Tapentadol SR	87,542	140		86,804	140	

[1] Approximated by odds ratio

SR = sustained release; CR = controlled release; RR = relative risk; CI = confidence interval
