# Supplementary material for: Short-term risk of falls among initiators of controlled-release tapentadol versus oxycodone in New South Wales between 2014 and 2020: A population-based retrospective cohort study
Source: PLoS Med. 2026 Aug 11;23(8):e1004950. doi: 10.1371/journal.pmed.1004950 (PMC13460682; doi:10.1371/journal.pmed.1004950)
Supplement: Supplementary file 11 [file pmed.1004950.s011.pdf]

S10 Table. Baseline characteristics of tapentadol (SR) and oxycodone (CR) initiators with only study opioids dispensed on initiation date and no exposure to non-study opioids in the previous 90 days, before and after propensity score matching

Characteristic	Unmatched			Matched		
	Tapentadol SR N = 56,947	Oxycodone CR N = 171,359	SD	Tapentadol SR N = 56,293	Oxycodone CR N = 56,293	SD
Year of initiation*						
2014	1,289 (2.3%)	9,636 (5.6%)	.17	1,288 (2.3%)	1,288 (2.3%)	.00
2015	4,695 (8.2%)	30,861 (18.0%)	.29	4,690 (8.3%)	4,690 (8.3%)	.00
2016	5,729 (10.1%)	34,130 (19.9%)	.28	5,718 (10.2%)	5,718 (10.2%)	.00
2017	9,446 (16.6%)	34,389 (20.1%)	.09	9,438 (16.8%)	9,438 (16.8%)	.00
2018	10,952 (19.2%)	28,019 (16.4%)	.08	10,922 (19.4%)	10,922 (19.4%)	.00
2019	14,060 (24.7%)	21,652 (12.6%)	.31	13,959 (24.8%)	13,959 (24.8%)	.00
2020	10,776 (18.9%)	12,672 (7.4%)	.35	10,278 (18.3%)	10,278 (18.3%)	.00
Sex						
Female	33,443 (58.7%)	95,072 (55.5%)	.07	32,995 (58.6%)	33,244 (59.1%)	.01
Male	23,504 (41.3%)	76,287 (44.5%)		23,298 (41.4%)	23,049 (40.9%)	
Age group (years)						
18-44	10,780 (18.9%)	33,623 (19.6%)	.02	10,717 (19.0%)	11,256 (20.0%)	.02
45-64	19,549 (34.3%)	51,926 (30.3%)	.09	19,241 (34.2%)	18,947 (33.7%)	.01
65-84	23,226 (40.8%)	68,187 (39.8%)	.02	22,959 (40.8%)	22,400 (39.8%)	.02
85 plus	3,392 (6.0%)	17,623 (10.3%)	.16	3,376 (6.0%)	3,690 (6.6%)	.02
Remoteness Area						
Major cities	38,552 (67.7%)	114,502 (66.8%)	.02	38,137 (67.8%)	38,101 (67.7%)	.00
Inner regional	14,357 (25.2%)	41,855 (24.4%)	.02	14,143 (25.1%)	14,070 (25.0%)	.00
Outer regional	3,543 (6.2%)	13,047 (7.6%)	.05	3,521 (6.3%)	3,630 (6.5%)	.01
Remote	213 (0.4%)	1,033 (0.6%)	.03	212 (0.4%)	229 (0.4%)	.00
Very remote	31 (0.1%)	97 (0.1%)	.00	30 (0.1%)	25 (0.0%)	.00
Missing	251 (0.4%)	825 (0.5%)	.01	250 (0.4%)	238 (0.4%)	.00
Decile of relative disadvantage						
1 (most disadvantage)	4,008 (7.0%)	12,936 (7.6%)	.02	3,979 (7.1%)	3,831 (6.8%)	.01

Characteristic	Unmatched			Matched		
	Tapentadol SR N = 56,947	Oxycodone CR N = 171,359	SD	Tapentadol SR N = 56,293	Oxycodone CR N = 56,293	SD
2	4,883 (8.6%)	14,841 (8.7%)	.00	4,823 (8.6%)	4,862 (8.6%)	.00
3	4,352 (7.6%)	13,746 (8.0%)	.01	4,315 (7.7%)	4,270 (7.6%)	.00
4	6,360 (11.2%)	20,596 (12.0%)	.03	6,295 (11.2%)	6,521 (11.6%)	.01
5	7,430 (13.1%)	18,632 (10.9%)	.07	7,268 (12.9%)	7,108 (12.6%)	.01
6	6,461 (11.4%)	19,091 (11.1%)	.01	6,394 (11.4%)	6,331 (11.3%)	.00
7	5,842 (10.3%)	17,534 (10.2%)	.00	5,771 (10.3%)	5,838 (10.4%)	.00
8	5,080 (8.9%)	15,714 (9.2%)	.01	5,036 (9.0%)	5,137 (9.1%)	.01
9	6,785 (11.9%)	20,357 (11.9%)	.00	6,719 (11.9%)	6,743 (12.0%)	.00
10 (least disadvantage)	5,482 (9.6%)	17,059 (10.0%)	.01	5,431 (9.7%)	5,407 (9.6%)	.00
Missing	264 (0.5%)	853 (0.5%)	.00	262 (0.5%)	245 (0.4%)	.00
Comorbidities						
Cancer	4,939 (8.7%)	21,643 (12.6%)	.13	4,917 (8.7%)	4,592 (8.2%)	.02
Substance use disorder	1,912 (3.4%)	6,018 (3.5%)	.01	1,895 (3.4%)	1,935 (3.4%)	.00
Prior overdose	85 (0.2%)	253 (0.2%)	.00	83 (0.2%)	79 (0.1%)	.00
Depression	16,902 (29.7%)	45,941 (26.8%)	.06	16,608 (29.5%)	16,918 (30.1%)	.01
Anxiety	5,716 (10.0%)	15,980 (9.3%)	.02	5,632 (10.0%)	5,408 (9.6%)	.01
Hypertension	11,666 (20.5%)	35,701 (20.8%)	.01	11,466 (20.4%)	11,990 (21.3%)	.02
Atrial fibrillation or flutter	1,206 (2.1%)	5,339 (3.1%)	.06	1,199 (2.1%)	1,234 (2.2%)	.00
Diabetes	8,575 (15.1%)	27,914 (16.3%)	.03	8,474 (15.1%)	8,704 (15.5%)	.01
Congestive heart failure	7,538 (13.2%)	27,269 (15.9%)	.08	7,458 (13.3%)	7,792 (13.8%)	.02
Thyroid disease	4,874 (8.6%)	14,624 (8.5%)	.00	4,799 (8.5%)	4,969 (8.8%)	.01
Postural hypotension	290 (0.5%)	1,117 (0.7%)	.02	286 (0.5%)	301 (0.5%)	.00
Osteoporosis	7,067 (12.4%)	25,850 (15.1%)	.08	7,006 (12.5%)	6,941 (12.3%)	.00
Stroke	11,852 (20.8%)	40,530 (23.7%)	.07	11,744 (20.9%)	11,115 (19.7%)	.03
Renal impairment	832 (1.5%)	4,198 (2.5%)	.07	823 (1.5%)	803 (1.4%)	.00
Hepatic impairment	413 (0.7%)	2,214 (1.3%)	.06	411 (0.7%)	318 (0.6%)	.02
Surgery (prior 30 days)	11,295 (19.8%)	36,580 (21.4%)	.04	11,149 (19.8%)	10,378 (18.4%)	.03

Characteristic	Unmatched			Matched		
	Tapentadol SR<br>N = 56,947	Oxycodone CR<br>N = 171,359	SD	Tapentadol SR<br>N = 56,293	Oxycodone CR<br>N = 56,293	SD
Falls-risk medicines (FRIDs; prior 30 days)						
Yes	26,981 (47.4%)	81,561 (47.6%)	.00	26,604 (47.3%)	26,788 (47.6%)	.01
Number of FRIDs:						
1	16,129 (59.8%)	46,624 (57.2%)	.05	15,911 (59.8%)	15,459 (57.7%)	.04
2	7,410 (27.5%)	23,035 (28.2%)	.02	7,304 (27.5%)	7,575 (28.3%)	.02
3	2,594 (9.6%)	8,710 (10.7%)	.04	2,547 (9.6%)	2,761 (10.3%)	.02
4	666 (2.5%)	2,503 (3.1%)	.04	660 (2.5%)	782 (2.9%)	.03
5+	182 (0.7%)	689 (0.8%)	.02	182 (0.7%)	211 (0.8%)	.01
Type of FRID						
Medicines associated with high risk of falls						
Antipsychotics (excluding lithium)	729 (2.7%)	2,956 (3.6%)	.05	718 (2.7%)	962 (3.6%)	.05
Anxiolytics	1,334 (4.9%)	4,010 (4.9%)	.00	1,314 (4.9%)	1,374 (5.1%)	.01
Hypnotics and sedatives	1,084 (4.0%)	3,745 (4.6%)	.03	1,072 (4.0%)	1,037 (3.9%)	.01
Antidepressants	9,118 (33.8%)	25,832 (31.7%)	.05	8,945 (33.6%)	9,681 (36.1%)	.05
Medicines that cause orthostatism/hypotension						
Vasodilators used in cardiac disease (e.g. nitrates)	745 (2.8%)	3,312 (4.1%)	.07	740 (2.8%)	854 (3.2%)	.02
Antihypertensives	1,102 (4.1%)	3,143 (3.9%)	.01	1,086 (4.1%)	1,044 (3.9%)	.01
Diuretics	1,701 (6.3%)	6,979 (8.6%)	.09	1,688 (6.3%)	1,917 (7.2%)	.03
Beta blockers	4,178 (15.5%)	14,311 (17.6%)	.06	4,141 (15.6%)	4,310 (16.1%)	.01
Calcium channel blockers	4,495 (16.7%)	14,704 (18.0%)	.04	4,429 (16.7%)	4,558 (17.0%)	.01
Renin-angiotensin acting agents	16,621 (61.6%)	49,576 (60.8%)	.02	16,393 (61.6%)	16,165 (60.3%)	.03
Alpha adrenoreceptor blockers	631 (2.3%)	2,018 (2.5%)	.01	623 (2.3%)	588 (2.2%)	.01
Dopaminergic agents	598 (2.2%)	1,818 (2.2%)	.00	592 (2.2%)	626 (2.3%)	.01
Other medicines associated with falls (prior 30 days)						
Gabapentinoids	5,082 (8.9%)	9,672 (5.6%)	.13	4,861 (8.6%)	4,575 (8.1%)	.02
Anticholinergic medicines	17 (0.0%)	75 (0.0%)	.01	17 (0.0%)	11 (0.0%)	.01
Statins	14,638 (25.7%)	43,867 (25.6%)	.00	14,419 (25.6%)	14,505 (25.8%)	.00

Characteristic	Unmatched			Matched		
	Tapentadol SR N = 56,947	Oxycodone CR N = 171,359	SD	Tapentadol SR N = 56,293	Oxycodone CR N = 56,293	SD
Hospital frailty risk score						
Low: <5	51,001 (89.6%)	145,753 (85.1%)	.14	50,387 (89.5%)	50,374 (89.5%)	.00
Medium: 5-15	4,527 (8.0%)	18,085 (10.6%)	.09	4,499 (8.0%)	4,403 (7.8%)	.01
High: >15	1,419 (2.5%)	7,521 (4.4%)	.10	1,407 (2.5%)	1,516 (2.7%)	.01
History of falls						
Falls in the past 2 years to 60 days	1,938 (3.4%)	8,258 (4.8%)	.07	1,920 (3.4%)	2,045 (3.6%)	.01

Note: cohort matched on propensity score, year and month of initiation

* study period: 01 September 2014 – 03 December 2020

SR = sustained release; CR = controlled release; SD = absolute standardised difference
