# Supplementary material for: Short-term risk of falls among initiators of controlled-release tapentadol versus oxycodone in New South Wales between 2014 and 2020: A population-based retrospective cohort study
Source: PLoS Med. 2026 Aug 11;23(8):e1004950. doi: 10.1371/journal.pmed.1004950 (PMC13460682; doi:10.1371/journal.pmed.1004950)
Supplement: Supplementary file 12 [file pmed.1004950.s012.pdf]

S11 Table. Relative risks of falls and cataract surgeries among tapentadol (SR) and oxycodone (CR) initiators with only study opioids dispensed on initiation date and no exposure to non-study opioids in the prior 90 days

Outcome	Exposure	Unmatched			Matched		
		No. exposed	No. events	RR (95% CI)	No. exposed	No. events	Matched RR[1] (95% CI)
Falls resulting in ED presentation, hospitalisation, or death							
7 days post-initiation	Oxycodone CR	171,359	547	0.59 (0.48-0.73)	56,293	165	0.64 (0.50-0.81)
	Tapentadol SR	56,947	108		56,293	105	
14 days post-initiation	Oxycodone CR	171,359	846	0.56 (0.47-0.67)	56,293	247	0.63 (0.51-0.77)
	Tapentadol SR	56,947	158		56,293	155	
28 days post-initiation	Oxycodone CR	171,359	1,213	0.60 (0.52-0.68)	56,293	347	0.68 (0.57-0.80)
	Tapentadol SR	56,947	240		56,293	235	
Cataract surgeries (negative control outcome)							
7 days post-initiation	Oxycodone CR	171,359	57	0.90 (0.52-1.54)	56,293	14	1.21 (0.60-2.46)
	Tapentadol SR	56,947	17		56,293	17	
14 days post-initiation	Oxycodone CR	171,359	122	0.89 (0.61-1.29)	56,293	40	0.90 (0.57-1.41)
	Tapentadol SR	56,947	36		56,293	36	
28 days post-initiation	Oxycodone CR	171,359	246	1.02 (0.79-1.30)	56,293	79	1.05 (0.77-1.43)
	Tapentadol SR	56,947	83		56,293	83	

[1] Approximated by odds ratio

SR = sustained release; CR = controlled release; RR = relative risk; CI = confidence interval
