# Supplementary material for: Short-term risk of falls among initiators of controlled-release tapentadol versus oxycodone in New South Wales between 2014 and 2020: A population-based retrospective cohort study
Source: PLoS Med. 2026 Aug 11;23(8):e1004950. doi: 10.1371/journal.pmed.1004950 (PMC13460682; doi:10.1371/journal.pmed.1004950)
Supplement: Supplementary file 13 [file pmed.1004950.s013.pdf]

S12 Table. Baseline characteristics of tapentadol (SR) and oxycodone (CR) initiators between 1 September 2014 - 3 December 2020 who concurrently initiated oxycodone (IR), before and after propensity score matching

Characteristic	Unmatched			Matched		
	Tapentadol SR N = 8,470	Oxycodone CR N = 88,239	SD	Tapentadol SR N = 8,436	Oxycodone CR N = 8,436	SD
Year of initiation*						
2014	36 (0.4%)	5,298 (6.0%)	.32	35 (0.4%)	35 (0.4%)	.00
2015	207 (2.4%)	15,897 (18.0%)	.53	206 (2.4%)	206 (2.4%)	.00
2016	525 (6.2%)	17,316 (19.6%)	.41	523 (6.2%)	523 (6.2%)	.00
2017	1,393 (16.5%)	17,839 (20.2%)	.10	1,390 (16.5%)	1,390 (16.5%)	.00
2018	2,156 (25.5%)	14,490 (16.4%)	.22	2,150 (25.5%)	2,150 (25.5%)	.00
2019	2,745 (32.4%)	11,638 (13.2%)	.47	2,739 (32.5%)	2,739 (32.5%)	.00
2020	1,408 (16.6%)	5,761 (6.5%)	.32	1,393 (16.5%)	1,393 (16.5%)	.00
Sex						
Female	4,090 (48.3%)	43,011 (48.7%)	.01	4,074 (48.3%)	4,048 (48.0%)	.01
Male	4,380 (51.7%)	45,228 (51.3%)		4,362 (51.7%)	4,388 (52.0%)	
Age group (years)						
18-44	2,095 (24.7%)	20,224 (22.9%)	.04	2,091 (24.8%)	2,177 (25.8%)	.02
45-64	3,080 (36.4%)	30,954 (35.1%)	.03	3,071 (36.4%)	3,020 (35.8%)	.01
65-84	3,135 (37.0%)	33,468 (37.9%)	.02	3,126 (37.1%)	3,090 (36.6%)	.01
85 plus	160 (1.9%)	3,593 (4.1%)	.13	148 (1.8%)	149 (1.8%)	.00
Remoteness Area						
Major cities	5,591 (66.0%)	58,115 (65.9%)	.00	5,571 (66.0%)	5,651 (67.0%)	.02
Inner regional	2,286 (27.0%)	23,084 (26.2%)	.02	2,275 (27.0%)	2,232 (26.5%)	.01
Outer regional	503 (5.9%)	6,239 (7.1%)	.05	501 (5.9%)	485 (5.8%)	.01
Remote	40-45	345 (0.4%)	.01	39-44	30 (0.4%)	.02
Very remote	n.p.	41 (0.1%)	n.p.	n.p.	6 (0.1%)	n.p.
Missing	45 (0.5%)	415 (0.5%)	.01	45 (0.5%)	32 (0.4%)	.02
Decile of relative disadvantage						
1 (most disadvantage)	420 (5.0%)	5,187 (5.9%)	.04	418 (5.0%)	424 (5.0%)	.00

Characteristic	Unmatched			Matched		
	Tapentadol SR N = 8,470	Oxycodone CR N = 88,239	SD	Tapentadol SR N = 8,436	Oxycodone CR N = 8,436	SD
2	591 (7.0%)	7,302 (8.3%)	.05	591 (7.0%)	582 (6.9%)	.00
3	542 (6.4%)	6,838 (7.8%)	.05	540 (6.4%)	559 (6.6%)	.01
4	891 (10.5%)	10,431 (11.8%)	.04	888 (10.5%)	923 (10.9%)	.01
5	898 (10.6%)	9,361 (10.6%)	.00	895 (10.6%)	812 (9.6%)	.03
6	926 (10.9%)	10,282 (11.7%)	.02	923 (10.9%)	945 (11.2%)	.01
7	879 (10.4%)	8,807 (10.0%)	.01	874 (10.4%)	850 (10.1%)	.01
8	902 (10.7%)	9,071 (10.3%)	.01	900 (10.7%)	940 (11.1%)	.02
9	1,351 (16.0%)	10,877 (12.3%)	.10	1,340 (15.9%)	1,287 (15.3%)	.02
10 (least disadvantage)	1,021 (12.1%)	9,657 (10.9%)	.03	1,018 (12.1%)	1,079 (12.8%)	.02
Missing	49 (0.6%)	426 (0.5%)	.01	49 (0.6%)	35 (0.4%)	.02
Exposure to non-study opioids (prior 90 days)						
None	7,085 (83.7%)	70,507 (79.9%)	.10	7,071 (83.8%)	7,071 (83.8%)	.00
Yes	1,385 (16.4%)	17,732 (20.1%)		1,365 (16.2%)	1,365 (16.2%)	
Comorbidities						
Cancer	678 (8.0%)	11,954 (13.6%)	.18	666 (7.9%)	675 (8.0%)	.00
Substance use disorder	302 (3.6%)	3,481 (3.9%)	.02	301 (3.6%)	301 (3.6%)	.00
Prior overdose	n.p.	146 (0.2%)	n.p.	n.p.	n.p.	n.p.
Depression	1,904 (22.5%)	22,296 (25.3%)	.07	1,894 (22.5%)	1,933 (22.9%)	.01
Anxiety	726 (8.6%)	8,179 (9.3%)	.02	722 (8.6%)	695 (8.2%)	.01
Hypertension	1,863 (22.0%)	19,503 (22.1%)	.00	1,842 (21.8%)	1,953 (23.2%)	.03
Atrial fibrillation or flutter	151 (1.8%)	2,158 (2.5%)	.05	148 (1.8%)	159 (1.9%)	.01
Diabetes	1,008 (11.9%)	12,447 (14.1%)	.07	1,002 (11.9%)	1,022 (12.1%)	.01
Congestive heart failure	750 (8.9%)	10,209 (11.6%)	.09	739 (8.8%)	771 (9.1%)	.01
Thyroid disease	541 (6.4%)	6,240 (7.1%)	.03	539 (6.4%)	554 (6.6%)	.01
Postural hypotension	31 (0.4%)	422 (0.5%)	.02	30 (0.4%)	30 (0.4%)	.00
Osteoporosis	801 (9.5%)	11,034 (12.5%)	.10	791 (9.4%)	819 (9.7%)	.01
Stroke	1,986 (23.5%)	23,708 (26.9%)	.08	1,970 (23.4%)	1,813 (21.5%)	.04
Renal impairment	58 (0.7%)	1,424 (1.6%)	.09	54 (0.6%)	52 (0.6%)	.00

Characteristic	Unmatched			Matched		
	Tapentadol SR N = 8,470	Oxycodone CR N = 88,239	SD	Tapentadol SR N = 8,436	Oxycodone CR N = 8,436	SD
Hepatic impairment	60 (0.7%)	1,096 (1.2%)	.05	58 (0.7%)	45 (0.5%)	.02
Surgery (prior 30 days)	2,653 (31.3%)	30,871 (35.0%)	.08	2,635 (31.2%)	2,482 (29.4%)	.04
Falls-risk medicines (FRIDs; prior 30 days)						
Yes	3,276 (38.7%)	37,881 (42.9%)	.09	3,257 (38.6%)	3,275 (38.8%)	.00
Number of FRIDs:						
1	2,117 (64.6%)	22,838 (60.3%)	.09	2,109 (64.8%)	2,024 (61.8%)	.06
2	840 (25.6%)	10,371 (27.4%)	.04	835 (25.6%)	894 (27.3%)	.04
3	242 (7.4%)	3,524 (9.3%)	.07	236 (7.3%)	269 (8.2%)	.04
4	60 (1.8%)	902 (2.4%)	.04	60 (1.8%)	72 (2.2%)	.03
5+	17 (0.5%)	246 (0.7%)	.02	17 (0.5%)	16 (0.5%)	.00
Type of FRID						
Medicines associated with high risk of falls						
Antipsychotics (excluding lithium)	68 (2.1%)	1,073 (2.8%)	.05	68 (2.1%)	105 (3.2%)	.07
Anxiolytics	161 (4.9%)	2,137 (5.6%)	.03	161 (4.9%)	166 (5.1%)	.01
Hypnotics and sedatives	105 (3.2%)	1,762 (4.7%)	.07	104 (3.2%)	121 (3.7%)	.03
Antidepressants	1,042 (31.8%)	12,672 (33.5%)	.04	1,036 (31.8%)	1,052 (32.1%)	.01
Medicines that cause orthostatism/hypotension						
Vasodilators used in cardiac disease (e.g. nitrates)	46 (1.4%)	1,074 (2.8%)	.10	45 (1.4%)	73 (2.2%)	.06
Antihypertensives	110 (3.4%)	1,418 (3.7%)	.02	108 (3.3%)	136 (4.2%)	.04
Diuretics	160 (4.9%)	2,416 (6.4%)	.06	156 (4.8%)	156 (4.8%)	.00
Beta blockers	431 (13.2%)	5,655 (14.9%)	.05	427 (13.1%)	465 (14.2%)	.03
Calcium channel blockers	514 (15.7%)	6,396 (16.9%)	.03	510 (15.7%)	519 (15.9%)	.01
Renin-angiotensin acting agents	2,090 (63.8%)	23,001 (60.7%)	.06	2,079 (63.8%)	2,042 (62.4%)	.03
Alpha adrenoreceptor blockers	67 (2.1%)	812 (2.1%)	.01	65 (2.0%)	96 (2.9%)	.06
Dopaminergic agents	57 (1.7%)	620 (1.6%)	.01	56 (1.7%)	58 (1.8%)	.00
Other medicines associated with falls (prior 30 days)						
Gabapentinoids	626 (7.4%)	5,571 (6.3%)	.04	615 (7.3%)	564 (6.7%)	.02

Characteristic	Unmatched			Matched		
	Tapentadol SR N = 8,470	Oxycodone CR N = 88,239	SD	Tapentadol SR N = 8,436	Oxycodone CR N = 8,436	SD
Anticholinergic medicines	n.p.	23 (0.0%)	n.p.	n.p.	n.p.	n.p.
Statins	1,811 (21.4%)	19,632 (22.3%)	.02	1,797 (21.3%)	1,770 (21.0%)	.01
Hospital frailty risk score						
Low: <5	7,739 (91.4%)	76,838 (87.1%)	.14	7,718 (91.5%)	7,732 (91.7%)	.01
Medium: 5-15	583 (6.9%)	8,852 (10.0%)	.11	573 (6.8%)	547 (6.5%)	.01
High: >15	148 (1.8%)	2,549 (2.9%)	.08	145 (1.7%)	157 (1.9%)	.01
History of falls						
Falls in the past 2 years to 60 days	192 (2.3%)	2,872 (3.3%)	.06	191 (2.3%)	198 (2.4%)	.01

Note: cohort matched on propensity score, year and month of initiation, and exposure to non-study opioids in the prior 90 days

* study period: 01 September 2014 – 03 December 2020

SR = sustained release; CR = controlled release; IR = immediate release; SD = absolute standardised difference; n.p. = value not presented to avoid residual disclosure of small cells
