# Supplementary material for: Short-term risk of falls among initiators of controlled-release tapentadol versus oxycodone in New South Wales between 2014 and 2020: A population-based retrospective cohort study
Source: PLoS Med. 2026 Aug 11;23(8):e1004950. doi: 10.1371/journal.pmed.1004950 (PMC13460682; doi:10.1371/journal.pmed.1004950)
Supplement: Supplementary file 14 [file pmed.1004950.s014.pdf]

S13 Table. Relative risks of falls and cataract surgeries among tapentadol (SR) and oxycodone (CR) initiators who concurrently initiated oxycodone (IR)

Outcome	Exposure	Unmatched			Matched		
		No. exposed	No. events	RR (95% CI)	No. exposed	No. events	Matched RR[1] (95% CI)
Falls resulting in ED presentation, hospitalisation, or death							
7 days post-initiation	Oxycodone CR	88,239	257	0.57 (0.33-0.97)	8,436	24	0.58 (0.30-1.13)
	Tapentadol SR	8,470	14		8,436	14	
14 days post-initiation	Oxycodone CR	88,239	367	0.68 (0.45-1.03)	8,436	30	0.80 (0.47-1.37)
	Tapentadol SR	8,470	24		8,436	24	
28 days post-initiation	Oxycodone CR	88,239	498	0.65 (0.45-0.93)	8,436	40	0.78 (0.49-1.24)
	Tapentadol SR	8,470	31		8,436	31	
Cataract surgeries (negative control outcome)							
7 days post-initiation	Oxycodone CR	88,239	9	2.32 (0.50-10.71)	8,436	n.p.	1.00 (0.14-7.10)
	Tapentadol SR	8,470	n.p.		8,436	n.p.	
14 days post-initiation	Oxycodone CR	88,239	24	1.30 (0.39-4.32)	8,436	n.p.	1.00 (0.20-4.96)
	Tapentadol SR	8,470	n.p.		8,436	n.p.	
28 days post-initiation	Oxycodone CR	88,239	66	0.95 (0.41-2.18)	8,436	n.p.	1.20 (0.37-3.93)
	Tapentadol SR	8,470	6		8,436	6	

[1] Approximated by odds ratio

SR = sustained release; CR = controlled release; IR = immediate release; RR = relative risk; CI = confidence interval; n.p. = value not presented to avoid residual disclosure of small cells
