# Supplementary material for: Short-term risk of falls among initiators of controlled-release tapentadol versus oxycodone in New South Wales between 2014 and 2020: A population-based retrospective cohort study
Source: PLoS Med. 2026 Aug 11;23(8):e1004950. doi: 10.1371/journal.pmed.1004950 (PMC13460682; doi:10.1371/journal.pmed.1004950)
Supplement: Supplementary file 15 [file pmed.1004950.s015.pdf]

S14 Table. Average follow-up time (days), by exposure

Exposure	Unadjusted cohort					Matched cohort				
	No. exposed	Mean	Std Dev	Min	Max	No. exposed	Mean	Std Dev	Min	Max
Oxycodone CR	419,732	24.7	7.7	1	28	103,758	25.1	7.2	1	28
Tapentadol SR	103,924	25.3	7.1	1	28	103,758	25.3	7.1	1	28

Note: 99.5% censored
