# Supplementary material for: Short-term risk of falls among initiators of controlled-release tapentadol versus oxycodone in New South Wales between 2014 and 2020: A population-based retrospective cohort study
Source: PLoS Med. 2026 Aug 11;23(8):e1004950. doi: 10.1371/journal.pmed.1004950 (PMC13460682; doi:10.1371/journal.pmed.1004950)
Supplement: Supplementary file 16 [file pmed.1004950.s016.pdf]

S15 Table. Average follow-up time (days), by exposure, among initiators with no recent opioid exposure

Exposure	Unadjusted cohort					Matched cohort				
	No. exposed	Mean	Std Dev	Min	Max	No. exposed	Mean	Std Dev	Min	Max
Oxycodone CR	250,030	24.8	7.6	1	28	65,592	25.1	7.3	1	28
Tapentadol SR	65,715	25.0	7.3	1	28	65,592	25.0	7.3	1	28

Note: 99.5% censored
