# Supplementary material for: Short-term risk of falls among initiators of controlled-release tapentadol versus oxycodone in New South Wales between 2014 and 2020: A population-based retrospective cohort study
Source: PLoS Med. 2026 Aug 11;23(8):e1004950. doi: 10.1371/journal.pmed.1004950 (PMC13460682; doi:10.1371/journal.pmed.1004950)
Supplement: Supplementary file 17 [file pmed.1004950.s017.pdf]

S16 Table. Average follow-up time (days), by exposure, among initiators with recent exposure to opioids

Exposure	Unadjusted cohort					Matched cohort				
	No. exposed	Mean	Std Dev	Min	Max	No. exposed	Mean	Std Dev	Min	Max
Oxycodone CR	169,702	24.5	7.8	1	28	38,166	25.0	7.2	1	28
Tapentadol SR	38,209	25.6	6.6	1	28	38,166	25.6	6.6	1	28

Note: 99.4% censored
