# Supplementary material for: Short-term risk of falls among initiators of controlled-release tapentadol versus oxycodone in New South Wales between 2014 and 2020: A population-based retrospective cohort study
Source: PLoS Med. 2026 Aug 11;23(8):e1004950. doi: 10.1371/journal.pmed.1004950 (PMC13460682; doi:10.1371/journal.pmed.1004950)
Supplement: Supplementary file 18 [file pmed.1004950.s018.pdf]

S17 Table. E-values for relative risks of falls

Outcome	Exposure	No. exposed	No. events	Matched[1] RR	E-value	
					Estimate	CI[2]
Overall population						
7 days post-initiation	Oxycodone CR	103,758	330	0.57	2.88	2.27
	Tapentadol SR	103,758	190			
14 days post-initiation	Oxycodone CR	103,758	487	0.62	2.61	2.15
	Tapentadol SR	103,758	302			
28 days post-initiation	Oxycodone CR	103,758	652	0.70	2.22	1.85
	Tapentadol SR	103,758	457			
People aged 65 years and older						
7 days post-initiation	Oxycodone CR	199,754	1,128	0.57	2.92	2.18
	Tapentadol SR	46,241	152			
14 days post-initiation	Oxycodone CR	199,754	1,723	0.60	2.71	2.13
	Tapentadol SR	46,241	240			
28 days post-initiation	Oxycodone CR	199,754	2,480	0.67	2.36	1.91
	Tapentadol SR	46,241	364			
People aged 80 years and older						
7 days post-initiation	Oxycodone CR	67,439	701	0.62	2.61	1.71
	Tapentadol SR	12,563	93			
14 days post-initiation	Oxycodone CR	67,439	1,091	0.64	2.51	1.78
	Tapentadol SR	12,563	149			
28 days post-initiation	Oxycodone CR	67,439	1,606	0.68	2.32	1.73
	Tapentadol SR	12,563	224			

[1] Approximated by odds ratio

[2] upper limit of the confidence interval presented as RR < 1

SR = sustained release; CR = controlled release; RR = relative risk; CI = confidence interval
