# Supplementary material for: Short-term risk of falls among initiators of controlled-release tapentadol versus oxycodone in New South Wales between 2014 and 2020: A population-based retrospective cohort study
Source: PLoS Med. 2026 Aug 11;23(8):e1004950. doi: 10.1371/journal.pmed.1004950 (PMC13460682; doi:10.1371/journal.pmed.1004950)
Supplement: Supplementary file 19 [file pmed.1004950.s019.pdf]

S18 Table. Relative risks of cataract surgeries (negative control outcome) for the total study population, people aged 65+, and people aged 80+

Outcome	Exposure	Unmatched			Matched			
		No. exposed	No. events	RR (95% CI)	No. exposed	No. events	Matched RR[1] (95% CI)	Adjusted[2] RR[1] (95% CI)
Overall population								
7 days post-initiation	Oxycodone CR	419,732	112	1.12 (0.75-1.66)	103,758	22	1.41 (0.82-2.43)	1.26 (0.69-2.31)
	Tapentadol SR	103,924	31		103,758	31		
14 days post-initiation	Oxycodone CR	419,732	242	1.25 (0.97-1.62)	103,758	56	1.32 (0.93-1.87)	1.16 (0.81-1.68)
	Tapentadol SR	103,924	75		103,758	74		
28 days post-initiation	Oxycodone CR	419,732	521	1.19 (1.00-1.43)	103,758	117	1.31 (1.03-1.66)	1.21 (0.95-1.56)
	Tapentadol SR	103,924	154		103,758	153		
People aged 65 years and older								
7 days post-initiation	Oxycodone CR	199,754	93	1.11 (0.71-1.75)	41,546	18	1.28 (0.69-2.37)	1.06 (0.55-2.05)
	Tapentadol SR	46,241	24		41,546	23		
14 days post-initiation	Oxycodone CR	199,754	211	1.31 (0.99-1.73)	41,546	52	1.10 (0.75-1.60)	0.98 (0.66-1.46)
	Tapentadol SR	46,241	64		41,546	57		
28 days post-initiation	Oxycodone CR	199,754	450	1.27 (1.04-1.54)	41,546	101	1.19 (0.91-1.55)	1.13 (0.86-1.49)
	Tapentadol SR	46,241	132		41,546	120		
People aged 80 years and older								
7 days post-initiation	Oxycodone CR	67,439	28	1.34 (0.59-3.07)	10,783	n.p.	1.75 (0.51-5.98)	2.00 (0.50-8.00)
	Tapentadol SR	12,563	7		10,783	7		
14 days post-initiation	Oxycodone CR	67,439	67	1.44 (0.86-2.43)	10,783	14	1.07 (0.52-2.22)	1.07 (0.51-2.25)
	Tapentadol SR	12,563	18		10,783	15		
28 days post-initiation	Oxycodone CR	67,439	142	1.47 (1.04-2.10)	10,783	26	1.23 (0.73-2.07)	1.21 (0.72-2.04)
	Tapentadol SR	12,563	39		10,783	32		

[1] Approximated by odds ratio

[2] Conditional logistic regression models adjusted for simultaneous initiation of study and non-study opioids

SR = sustained release; CR = controlled release; RR = relative risk; CI = confidence interval; n.p. = value not presented to avoid residual disclosure of small cells
