# Supplementary material for: Short-term risk of falls among initiators of controlled-release tapentadol versus oxycodone in New South Wales between 2014 and 2020: A population-based retrospective cohort study
Source: PLoS Med. 2026 Aug 11;23(8):e1004950. doi: 10.1371/journal.pmed.1004950 (PMC13460682; doi:10.1371/journal.pmed.1004950)
Supplement: Supplementary file 20 [file pmed.1004950.s020.pdf]

S19 Table. Event rates and relative risks of falls, by source of presentation (ED visit, hospital admission, death), for the total study population

Outcome	Exposure	No. exposed	No. events	Matched RR[1] (95% CI)	Adjusted[2] RR[1] (95% CI)
7 days post-initiation					
ED presentation	Oxycodone CR	103,758	78	0.82 (0.59,1.14)	0.81 (0.58,1.13)
	Tapentadol SR	103,758	64		
Hospital admission	Oxycodone CR	103,758	290	0.55 (0.45,0.66)	0.55 (0.45,0.67)
	Tapentadol SR	103,758	159		
Death	Oxycodone CR	103,758	n.p.	n.p.	n.p.
	Tapentadol SR	103,758	n.p.		
14 days post-initiation					
ED presentation	Oxycodone CR	103,758	121	0.79 (0.60,1.03)	0.78 (0.59,1.02)
	Tapentadol SR	103,758	95		
Hospital admission	Oxycodone CR	103,758	422	0.60 (0.51,0.70)	0.59 (0.51,0.70)
	Tapentadol SR	103,758	254		
Death	Oxycodone CR	103,758	n.p.	n.p.	n.p.
	Tapentadol SR	103,758	n.p.		
14 days post-initiation					
ED presentation	Oxycodone CR	103,758	185	0.84 (0.68,1.04)	0.79 (0.64,0.99)
	Tapentadol SR	103,758	155		
Hospital admission	Oxycodone CR	103,758	548	0.69 (0.60,0.78)	0.67 (0.59,0.77)
	Tapentadol SR	103,758	377		
Death	Oxycodone CR	103,758	n.p.	n.p.	n.p.
	Tapentadol SR	103,758	n.p.		

Note: sources are not mutually exclusive; a fall event may be captured in more than one source (e.g., ED and hospital records). The sum of events across all sources may therefore exceed the total number of falls.

[1] Approximated by odds ratio

[2] Conditional logistic regression models adjusted for simultaneous initiation of study and non-study opioids

ED = emergency department; CR = controlled release; SR = sustained release; RR = relative risk; CI = confidence interval; n.p. = value not presented to avoid residual disclosure of small cells
