# Supplementary material for: Short-term risk of falls among initiators of controlled-release tapentadol versus oxycodone in New South Wales between 2014 and 2020: A population-based retrospective cohort study
Source: PLoS Med. 2026 Aug 11;23(8):e1004950. doi: 10.1371/journal.pmed.1004950 (PMC13460682; doi:10.1371/journal.pmed.1004950)
Supplement: Supplementary file 21 [file pmed.1004950.s021.pdf]

S1 Figure. Study design

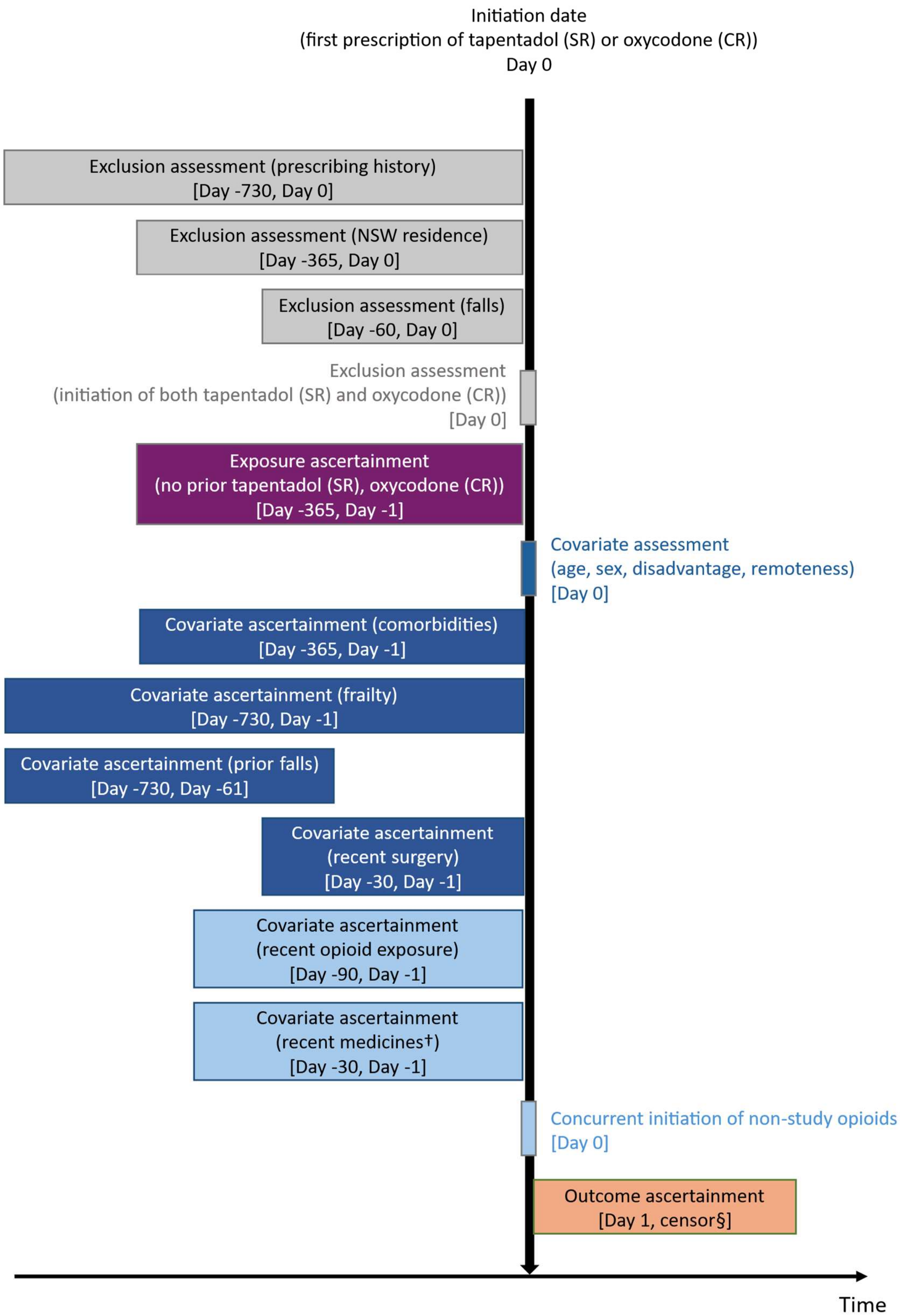

† Falls-risk medicines, gabapentinoids, anticholinergic medicines, statins

§ First of: outcome of interest, end of follow-up (7, 14, or 28 days), hospital admission (unrelated to falls), death, or study end date (31 December 2020)
