# Supplementary material for: Short-term risk of falls among initiators of controlled-release tapentadol versus oxycodone in New South Wales between 2014 and 2020: A population-based retrospective cohort study
Source: PLoS Med. 2026 Aug 11;23(8):e1004950. doi: 10.1371/journal.pmed.1004950 (PMC13460682; doi:10.1371/journal.pmed.1004950)

**S2 Figure. Propensity score distributions for tapentadol (SR) and oxycodone (CR) initiators**

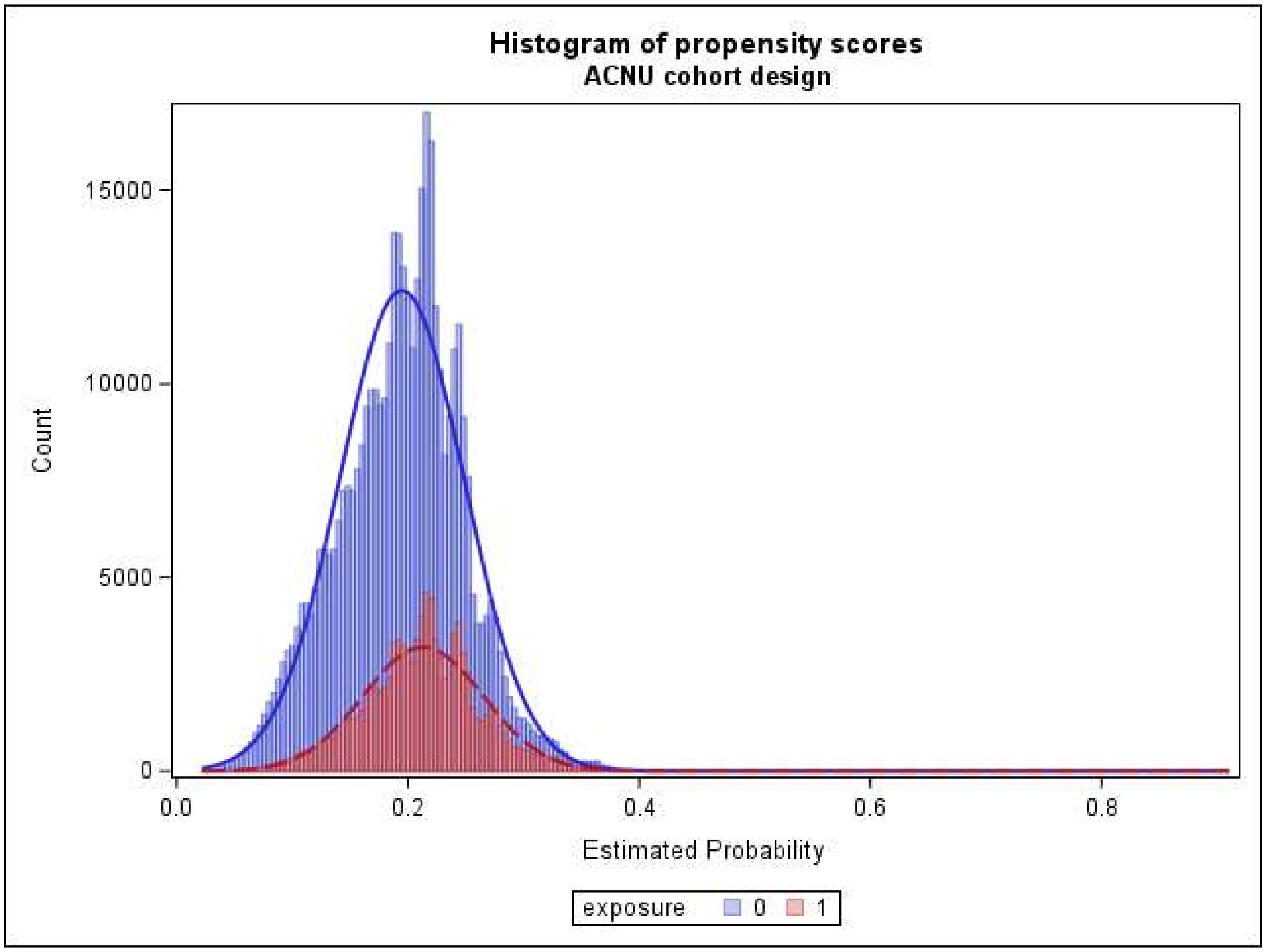

Supplement: Supplementary file 22 [file pmed.1004950.s022.pdf]
