# Supplementary material for: Short-term risk of falls among initiators of controlled-release tapentadol versus oxycodone in New South Wales between 2014 and 2020: A population-based retrospective cohort study
Source: PLoS Med. 2026 Aug 11;23(8):e1004950. doi: 10.1371/journal.pmed.1004950 (PMC13460682; doi:10.1371/journal.pmed.1004950)
Supplement: Supplementary file 23 [file pmed.1004950.s023.pdf]

S3 Figure. Cumulative incidence curves: falls following initiation of tapentadol (SR) or oxycodone (CR)

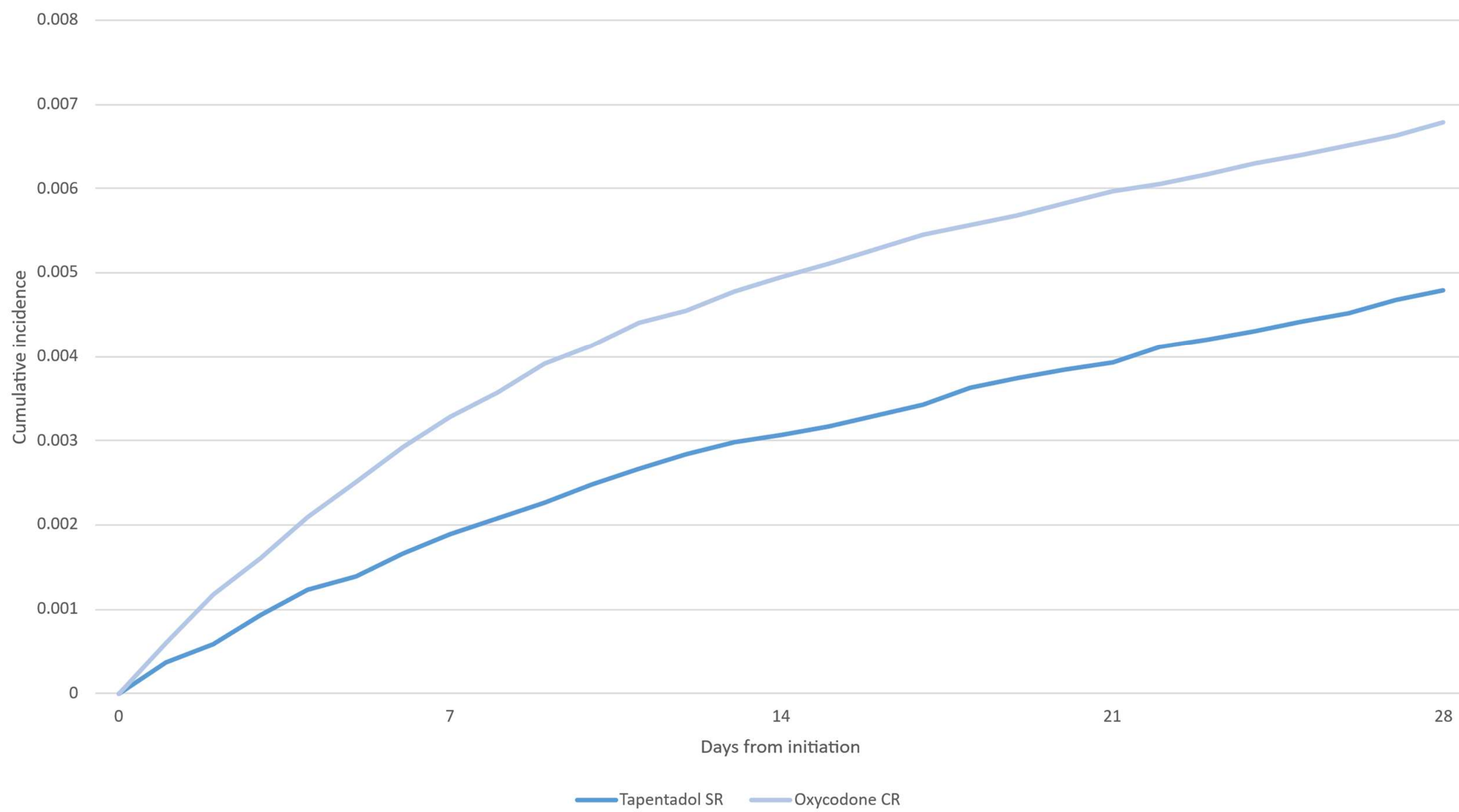

Number at risk

Tapentadol	103758	96900	92694	89965	87893
Oxycodone	103758	96603	92100	89264	87054
