# Supplementary material for: Short-term risk of falls among initiators of controlled-release tapentadol versus oxycodone in New South Wales between 2014 and 2020: A population-based retrospective cohort study
Source: PLoS Med. 2026 Aug 11;23(8):e1004950. doi: 10.1371/journal.pmed.1004950 (PMC13460682; doi:10.1371/journal.pmed.1004950)
Supplement: Supplementary file 24 [file pmed.1004950.s024.pdf]

S4 Figure. Cumulative incidence curves: falls following initiation of tapentadol (SR) or oxycodone (CR) among initiators with no recent opioid exposure

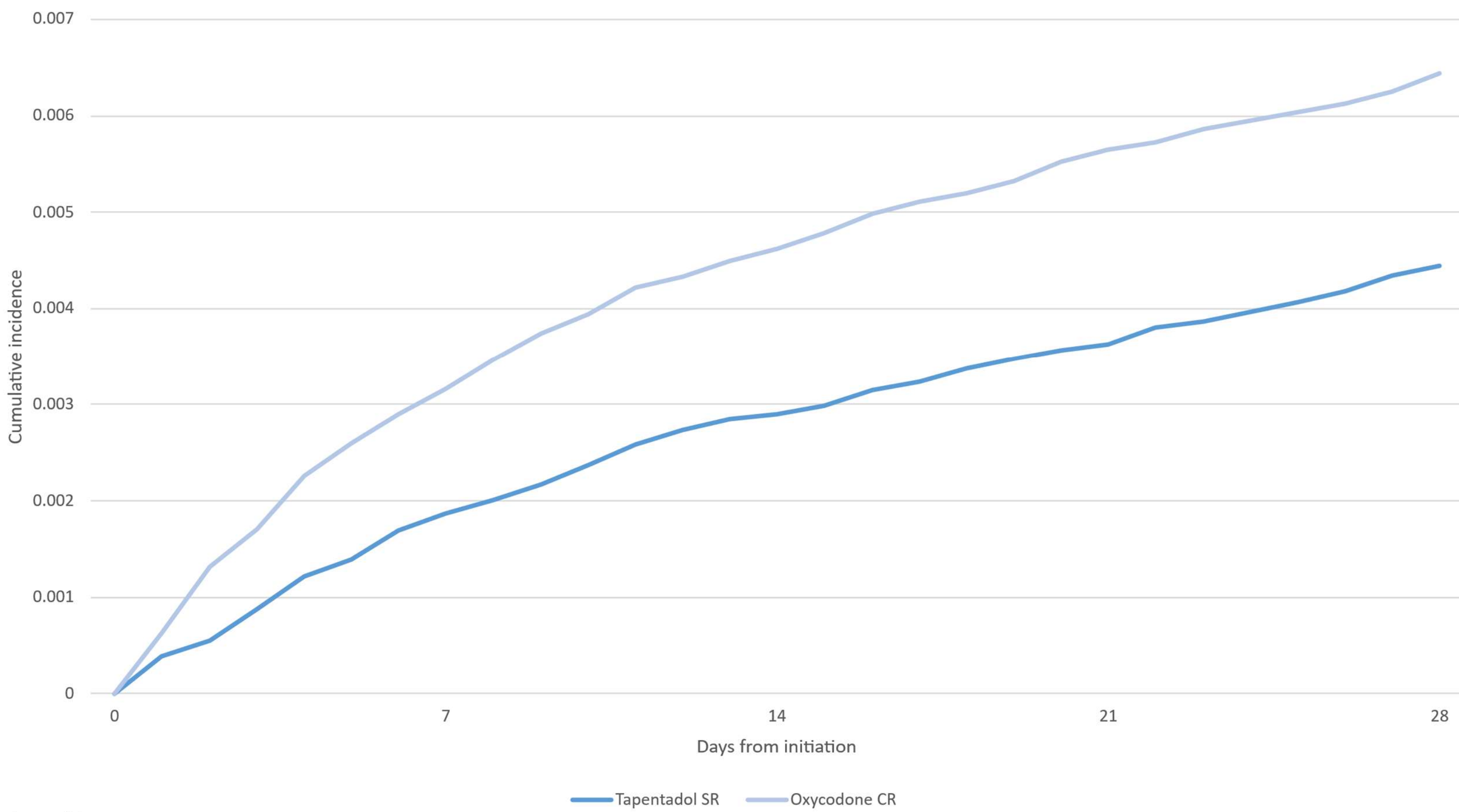

Number at risk

Tapentadol	65592	60767	57985	56262	55036
Oxycodone	65592	60966	58235	56584	55381
