# Supplementary material for: Short-term risk of falls among initiators of controlled-release tapentadol versus oxycodone in New South Wales between 2014 and 2020: A population-based retrospective cohort study
Source: PLoS Med. 2026 Aug 11;23(8):e1004950. doi: 10.1371/journal.pmed.1004950 (PMC13460682; doi:10.1371/journal.pmed.1004950)
Supplement: Supplementary file 25 [file pmed.1004950.s025.pdf]

S5 Figure. Cumulative incidence curves: falls following initiation of tapentadol (SR) or oxycodone (CR) among initiators with recent exposure to opioids

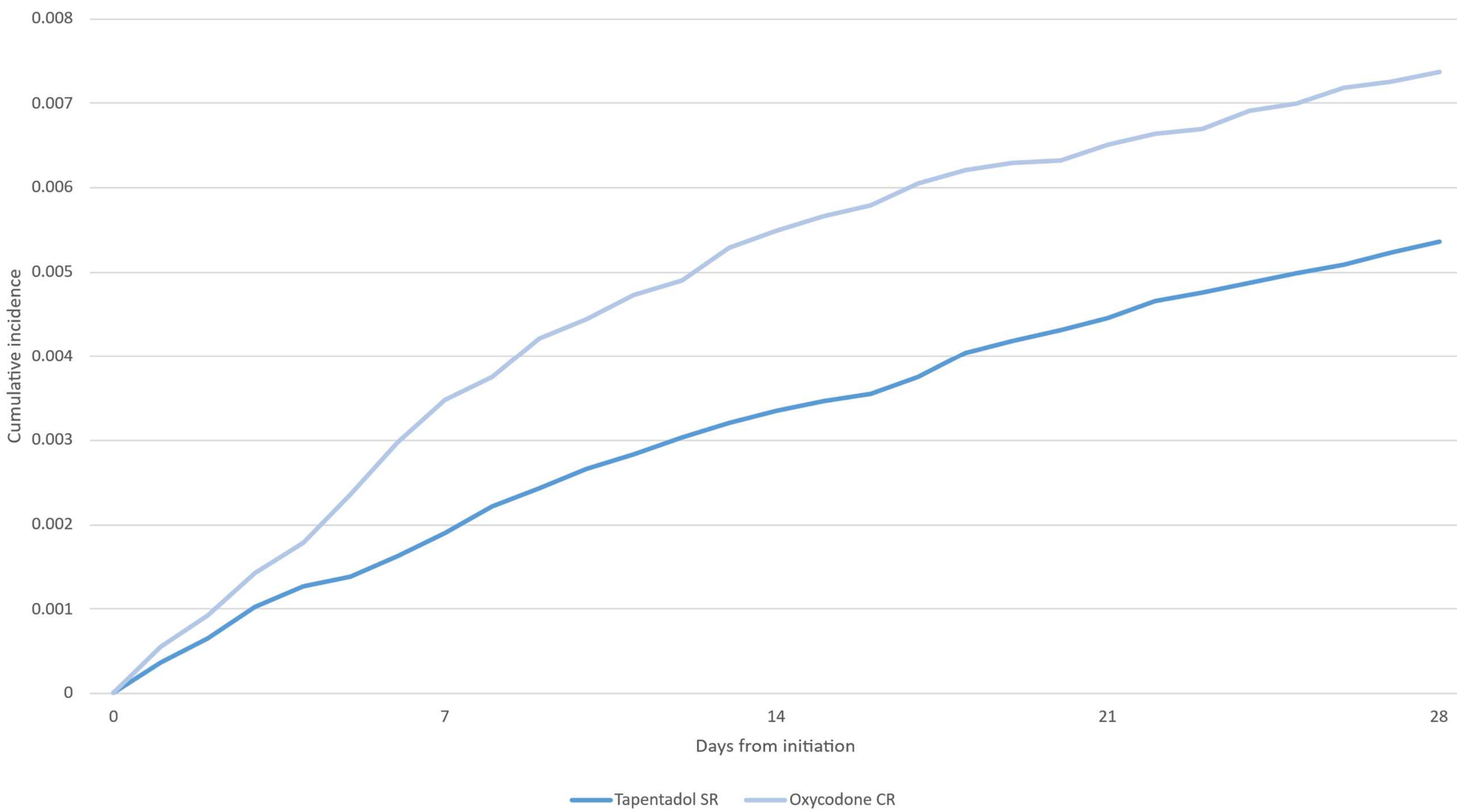

Number at risk

Tapentadol	38166	36133	34709	33703	32857
Oxycodone	38166	35637	33865	32680	31673
