# Supplementary material for: Short-term risk of falls among initiators of controlled-release tapentadol versus oxycodone in New South Wales between 2014 and 2020: A population-based retrospective cohort study
Source: PLoS Med. 2026 Aug 11;23(8):e1004950. doi: 10.1371/journal.pmed.1004950 (PMC13460682; doi:10.1371/journal.pmed.1004950)
Supplement: Supplementary file 26 [file pmed.1004950.s026.pdf]

MedIntel data platform

Statistical Analysis Plan

Comparing the risk of falls resulting in emergency department presentation, hospital admission, or death among initiators of tapentadol sustained-release (SR) vs initiators of oxycodone controlled-release (CR)

Prepared by Ximena Camacho, Ria Hopkins, Sallie Pearson, Andrea Schaffer, Jonathan Brett, David Henry, Kristian B. Filion, Steven Marsh, Natasa Gisev, Nicole Pratt

Contents

1 Background ... 3
2 Aims ... 3
3 Study Design ... 4
3.1 Data Sources ... 4
3.2 Study population ... 5
3.3 Exposure ... 6
3.3.1 Follow-up period ... 6
3.4 Outcomes ... 6
3.4.1 Cataract surgery (negative control outcome) ... 7
3.5 Covariates (potential confounders) ... 7
4 Statistical Analysis ... 9
4.1 Propensity score matching ... 9
4.2 Descriptive statistics ... 10
4.3 Analyses ... 10
4.4 Subgroup analyses ... 10
4.5 Sensitivity analyses ... 11
4.6 Potential sources of bias ... 11
5 Appendix ... 11
6 References ... 12

1 Background

- Tapentadol is being increasingly prescribed in Australia[1], and there is evidence that tapentadol is replacing oxycodone as the 'drug of choice' for post-surgical pain management[2]
- Use of publicly subsidised sustained release (SR) tapentadol is increasing in Australia, despite decreases in use of all other PBS-listed opioids[3]
- Most sustained-release tapentadol sold in Australia is publicly subsidised[1, 4]
- Opioids are associated with increased risk of falls compared to non-use[5, 6]; this risk is highest in the first 7 days following initiation[7, 8], and persists through first 28 days following initiation[7, 9]
- Real-world toxicity of tapentadol (SR) is not yet fully understood[10, 11]; nervous system disorders (dizziness, somnolence) were among the most common side effects reported in all tapentadol (SR) clinical trials (occurred in >= 1/10 people)[12].
- Nonetheless, tapentadol (SR) appears to have better tolerability[13] and lower incidence of serious adverse events when compared to most opioids[13, 14] including oxycodone[15]
- Furthermore, both pre-market and phase IV safety studies have identified a lower rate of dizziness (6.1% vs 10.9%) and somnolence (5.7% vs 7.4%) in tapentadol (SR) treatment arm vs oxycodone (CR), respectively[12, 16, 17].
- However, the populations studied in clinical trials were not reflective of those presenting in real-world clinical practice. In particular, trial participants were not permitted to use other non-study opioids; this does not reflect real-world clinical practice, where multiple opioids are often used in the management of pain[18].Furthermore, the trials were powered to assess efficacy in reducing pain and underpowered to assess safety outcomes (including falls and fall-related injuries).
- We therefore aim to assess the risk of falls resulting in emergency department (ED) presentations, hospital admissions, or death (hereafter referred to as "falls"), following initiation of tapentadol (SR) compared to a comparable drug, oxycodone (CR), in a real-world population that includes people who use other opioids alongside the opioids of interest.
- In light of the existing pre- and post-market clinical trial findings, we hypothesise that tapentadol (SR) is associated with lower incidence of falls compared to oxycodone (CR).

2 Aims

The aim of this study is to examine the short-term risk of falls resulting in ED presentation, hospital admission, or death, associated with initiating tapentadol (SR), compared to initiating a comparator drug (oxycodone controlled-release [CR]).

Specifically, we will assess:

1) Whether initiation of tapentadol (SR) is associated with falls compared to initiation of oxycodone (CR)

We will assess falls in all initiators of study medications. We will conduct subgroup analyses in people who are opioid-naïve, and by age (≥65, ≥80 years).

To assess the robustness of our findings, we will repeat our primary analyses using cataract surgery as a negative control outcome.

3 Study Design

We will use observational data and an active comparator new user cohort design to assess the risk of falls in adults among people who initiate tapentadol (SR) and oxycodone (CR).

3.1 Data Sources

This study will leverage the Medicines Intelligence Data Platform, held by the Medicines Intelligence Research Program at the School of Population Health, UNSW and described elsewhere[19]. The Medicines Intelligence Data Platform comprises longitudinal, linked population-level data for adults residing in NSW between 2005 and 2020 who were eligible for Medicare (i.e., registered in the Medicare Consumer Directory). The proposed analysis will use linked records from the following data sources:

Commonwealth data collections:

- Medicare Consumer Directory (MCD)
- Pharmaceutical Benefits Scheme (PBS)
- National Death Index (NDI)

NSW data collections:

- NSW Admitted Patient Data Collection (APDC)
- NSW Emergency Department Data Collection (EDDC)
- NSW Clinical Cancer Registry (CCR)

The APDC (hereafter referred to as ‘hospital data’) is a statutory data collection containing records of all hospital separations (including public hospitals, public psychiatric hospitals, public multi-purpose services, private hospitals, and private day procedure centres) in NSW. Data on diagnoses are coded according to the International Classification of Diseases, Version 10, Australian Modification (ICD-10-AM) and procedures coded according to the Australian Classification of Health Interventions (ACHI). Records are available for separation dates from 1 Jan 2002 to 31 Mar 2022.

The EDDC (referred to as `ED data’) is a data collection containing all visits to participating emergency departments in NSW. Data on diagnoses are coded according to the International Classification of Diseases, Version 9/10 or SNOMED-CT. Records are available for presentation dates from 1 Jan 2005 to 31 Mar 2022.

The PBS data contain claims for dispensed prescription medicines covered under the Pharmaceutical Benefits Scheme. All Medicare-eligible residents (i.e., Australian citizens, permanent residents, and foreigners with reciprocal health care agreements) are eligible to receive medicines subsidised by the PBS. The PBS covers dispensings

from community pharmacies and private hospitals; information on medicines provided in acute care, emergency departments and by private prescriptions is not available. PBS data are available for 1 Jan 2002 to 31 Mar 2022.

The CCR is a statutory data collection containing all notifications of primary malignant neoplasms from 1 Jan 1972 to 31 Dec 2019.

Date and fact of death data are available from the NDI for 1 Jan 2002 to 31 May 2022; cause of death information is available up to 31 December 2020.

3.2 Study population

The study population will comprise all adults (aged 18+ on the date of initiation) who initiate either tapentadol (SR) (exposure) or oxycodone (CR) (comparator) between 1 September 2014 and 3 December 2020. (Note: oxycodone CR was chosen as the comparator to align with pre-approval clinical trials of tapentadol; oxycodone CR was the comparator most often used in trials[13]. The comparator group will include both oxycodone (CR) and combination oxycodone (CR) + naloxone products.)

The index date will be defined as the first date of initiation with either tapentadol (SR) or oxycodone (CR) during the study period.

Initiation

As tapentadol was PBS-listed on 1 June 2014, we will consider the period between 1 June – 31 August 2014 as a washout to allow for the possibility of private dispensing prior to subsidy; any individuals with a dispensing for tapentadol (SR) during this period will be excluded.

Initiation of tapentadol (SR) will be defined as a dispensing during the study period with no prior dispensings of tapentadol (SR) or oxycodone (CR) in the previous 365 days.

Initiation of oxycodone (CR) will be defined as a dispensing during the study period with no prior dispensings of tapentadol (SR) or oxycodone (CR) in the previous 365 days.

For people meeting the initiation criteria more than once, we will only include the first instance.

Cohort exit

People will exit the cohort at the earliest of:

- Event (defined below)
- End of follow-up period:
    - 7 days after initiation
    - 14 days after initiation
    - 28 days after initiation
- Admission to hospital (for any cause)

- End of study (31 December 2020)
- Death

We will exclude the following people from our study:

- individuals with a death date prior to initiation (data quality issue)
- individuals with a death date on initiation date
- initiators who were not residents of NSW at the time of initiation.
- initiators who lived in NSW for less than 1 year prior to initiation, to ensure sufficient data for ascertainment of comorbidities (1-year lookback) and prior falls.
- individuals with no dispensings in the PBS data in the 2 years prior to initiation.
- individuals with dispensings of either tapentadol (SR) or oxycodone (CR) in the year prior to initiation (e.g., exclude anyone initiating tapentadol (SR) who has a dispensing for oxycodone (CR) in the prior year).
- individuals with dispensings for both tapentadol (SR) and oxycodone (CR) on the same date.
- Individuals with a fall resulting in hospitalisation or ED visit in the 60 days prior to and including initiation.

3.3 Exposure

We will define exposure using an intention-to-treat approach, where people are assumed to be exposed to the initiation drug (tapentadol (SR) or oxycodone (CR)) for the duration of follow-up[20].

We will define tapentadol (SR) exposure using PBS item codes 10096J, 10094G, 10100N, 10091D, 10092E (Appendix 1).

We will define oxycodone (CR) exposure using ATC codes N02AA05 and N02AA55 (see Appendix 2 for definitions).

3.3.1 Follow-up period

We will use a 7-day, 14-day, and 28-day follow-up period from the date of initiation (index date) to ascertain outcomes. The maximum 28-day period was chosen as it is the period of highest risk for falls[7-9].

3.4 Outcomes

Falls resulting in ED presentation, hospitalisation, or death ("falls")

We will define falls as any hospitalisation, emergency department presentation, or death related to a fall, occurring during the exposed follow-up period. We will identify falls within 7 days, 14 days, and 28 days of initiation.

Define falls as follows:

- APDC: ICD-10-AM codes W00-W19 in any diagnosis field (principal or other) (Appendix 3), where the admission date occurs within the outcome

ascertainment window (7 / 14 / 28 days) and fall is not classified as an in-hospital event (i.e., condition onset flag ^= 1 [condition with onset during the episode of care]
- EDDC: ICD-10-AM codes W00-W19 (Appendix 3) or ICD-9 codes E880-E886, E888 (Appendix 4) or SNOMED-CT-AU codes (Appendix 5) in any diagnosis field, where the presentation date occurs within the outcome ascertainment window (7 / 14 / 28 days)
- NDI: ICD-10 codes W00-W19 in any cause of death field (underlying or other cause of death) where the date of death occurs within the outcome ascertainment window (Appendix 3)

3.4.1 Cataract surgery (negative control outcome)

We will define cataract surgery as any hospitalisation related to cataract surgery occurring during the exposed follow-up period. We will identify cataract surgeries within 7 days, 14 days, and 28 days following initiation. Define cataract surgeries as follows:

- APDC: any ACHI procedure code or ACHI procedure block in any position (principal or other) corresponding to cataract surgery (Appendix 7), where the admission date occurs within the outcome ascertainment window

3.5 Covariates (potential confounders)

We will ascertain the following:

Demographic characteristics

- Sex
- Age (continuous, modelled as a spline)
- Age (grouped as 18-44, 45-64, 65-84, 85+)
- Remoteness
    - Determined using the Australian Statistical Geographic Standard Remoteness Structure
- Relative disadvantage
    - Determined using the Socio-Economic Indexes for Areas (SEIFA) Index of Relative Socio-economic Disadvantage

Other opioids dispensed (initiated) at initiation

Defined as any dispensing for any other opioid (other than tapentadol [SR] and oxycodone [CR]) on the tapentadol/oxycodone initiation date, where there were no dispensings for that non-study opioid in the prior year. Define non-study opioids using ATC codes and PBS items (Appendix 8). Define as binary (1 = any non-study opioid, 0 = none) and by individual opioid type.

Recent opioid exposure

Defined as any dispensing for any non-study opioid (i.e. non-tapentadol (SR), non-oxycodone (CR)) in the 90 days prior to initiation (not including initiation date). Define non-study opioids using ATC codes and PBS items (Appendix 8).

Define as binary (1 = yes, recent exposure; 0 = no recent exposure).

Comorbidities

Define the following comorbidities in the 365 days prior to initiation, not including initiation date. See Appendix 9 for definitions.

- Cancer (excluding non-melanoma skin cancers)
- History of substance abuse
- Prior overdose
- Depression
- Anxiety
- Hypertension
- Atrial fibrillation or flutter
- Diabetes
- Congestive heart failure
- Thyroid disease (over or under active)
- Postural hypotension
- Osteoporosis
- Stroke
- Renal impairment
- Hepatic impairment

Recent surgery

Defined as any inpatient hospital procedure with an ACHI surgical procedure code (defined according to ACHI code blocks identified in Gong et al. [23]) in the 30 days prior to study drug initiation[24]. Define as binary (1 = recent surgery, 0 = no recent surgery).

Recent exposure to gabapentinoids

Defined as any dispensing for any gabapentinoid (ATC N03AX12, N03AX16, N02BG) in the 30 days prior to study drug initiation.

Recent exposure to anticholinergic medicines

Defined as any dispensing for an anticholinergic medicine (ATC N04A)[25] in the 30 days prior to study drug initiation.

Recent exposure to statins

Defined as any dispensing for any statin (ATC C10) in the 30 days prior to study drug initiation[26-28].

Exposure to falls-risk medicines (FRIDs)

Defined as any dispensing of any medicine with a known risk of falls in the 30 days prior to initiation (Appendix 10). We will use an established list of Falls-Risk-Increasing Drugs (FRIDs) that has been used extensively in the literature and includes medicines known to increase the risk of falls and to cause orthostatism and hypotension[5, 29]. Exposure will be defined as a binary measure (1 = recent exposure to FRIDs, 0 = no recent exposure to FRIDs).

Hospital frailty risk score

We will define baseline frailty using the Hospital Frailty Risk Score defined by Gilbert et al[30]. The Hospital Frailty Risk Score is a validated method of deriving a frailty score from routinely-collected inpatient data, using ICD-10 diagnosis codes with individual weightings (Appendix 11). We will group the frailty score into three levels (low: <5, medium: 5-15, and high: >15), where the score is calculated using diagnosis codes from all inpatient admissions in the APDC from the previous 24 months[31].

Prior falls

We will look for evidence of falls resulting in ED presentation or hospitalisation (defined as above) in the 2 years to 61 days (inclusive) prior to index date.

4 Statistical Analysis

The reporting of this study will be done in accordance with the Reporting of studies Conducted using Observational Routinely collected health Data for Pharmaco-Epidemiology (RECORD-PE) statement[32] and CODE-EHR best practice framework[33]. All analyses will use a type I error rate of 0.05 as the threshold for statistical significance and will be performed using SAS (v9.4; SAS Institute, Cary NC).

4.1 Propensity score matching

We will use propensity scores to adjust for baseline confounding and ensure comparability (exchangeability) of the exposure and comparator groups. The estimand of interest is the average treatment effect in the treated (ATT). We will define a propensity score model using logistic regression, where exposure group (tapentadol SR / oxycodone CR) is the dependent variable and all of the covariates defined in Section 3.5 above (except for dispensing of non-study opioids at initiation date) are independent predictors.

In order to carefully account for underlying trends in opioid dispensing (i.e., increasing dispensing of tapentadol over time and changes to opioid prescribing policies [including upscheduling of codeine]) and the impact of prior opioid exposure, we will

match each tapentadol (SR) initiator to an oxycodone (CR) initiator (1:1) using greedy matching based on propensity score, month/year of initiation, and recent opioid exposure as follows:

- Calendar year and month of initiation
- Recent opioid exposure / no recent exposure
- Propensity score (caliper: 0.2 * standard deviation of the logit of the propensity score)

We will assess covariate balance for the matched tapentadol and oxycodone groups, with standardized differences >0.1 considered unbalanced[34].

4.2 Descriptive statistics

Descriptive statistics will be used to summarise baseline characteristics of the unmatched tapentadol (SR) and oxycodone (CR) initiator cohorts. We will describe categorical variables using number (%); and continuous variables will be summarised using median (interquartile range [IQR]). Standardized differences will be used to assess the balance between the exposed and comparator groups.

Descriptive statistics will be used to summarise baseline characteristics of the matched tapentadol (SR) and oxycodone (CR) initiator cohorts as above, and standardized differences will be used to assess the balance between the matched exposed and comparator populations.

4.3 Analyses

For each outcome (including the negative control outcome), we will separately conduct the following:

We will use logistic regression approaches to calculate the relative risk of the outcome under the exposure and comparator treatment. (Note that the logistic models will estimate the odds ratio [OR]; we will use the OR to approximate the relative risk). We will calculate the relative risk of outcomes at 7 days, 14 days, and 28 days, and calculate robust standard errors to account for the matched data. We will calculate unadjusted and adjusted estimates; adjusted models will include dispensing of non-study opioids at initiation, as well as any other covariates that remain unbalanced after matching.

4.4 Subgroup analyses

We will explore risks of all outcomes among the following subgroups:

- People with no recent opioid exposure / recent opioid exposure
- People aged ≥65 years at initiation
- People aged ≥80 years at initiation

4.5 Sensitivity analyses

Additional sensitivity analyses include:

- Exclude all people with any other opioids (other than tapentadol (SR) / oxycodone (CR)) dispensed on the initiation date
- Restrict the study population to people who initiated an immediate-release opioid alongside study opioids (tapentadol (SR) / oxycodone (CR))
- Calculate E-value (assess minimum strength required for an unmeasured confounder to explain away observed association)

4.6 Potential sources of bias

- The PBS data does not capture prescriptions that are privately funded. Importantly, immediate-release (IR) tapentadol is not subsidised by the PBS, and therefore not captured in the data. Initiators who appear to have no recent exposure to opioids may have purchased tapentadol privately and may be therefore misclassified. There may also be concurrent use or switching between PBS-subsidised and privately purchased opioids.
- The PBS data does not capture opioids administered in inpatient settings or on discharge from public hospitals in NSW. We will therefore underestimate true prescription opioid use. This may lead to misclassification of (no) recent opioid use.
- We can only observe opioid dispensings but cannot confirm whether the medicines were ingested, taken as prescribed, or actual consumed dose. We also do not have information on prescribed daily dose.
- We will likely underestimate covariates that rely solely on hospital admissions (e.g., history of substance abuse).
- Changes in health care delivery and health-seeking behaviours during the COVID-19 pandemic in 2020-2021 may have impacted observations. This is unlikely to cause bias but may result in smaller numbers of events during periods of heightened restrictions and limited activity levels.

5 Appendix

<see attached file>

6 References

1. Peacock, A., et al., *Opioid use and harms associated with a sustained-release tapentadol formulation: A post-marketing surveillance study.* Drug Alcohol Depend, 2020. **206**: p. 107697.
2. Mirabella, J., et al., *Prescribing trend of tapentadol in a Sydney local health district.* Br J Clin Pharmacol, 2022. **88**(9): p. 3929-3935.
3. Department of Health and Aged Care, *Impact of regulatory reforms on utilisation of opioids: Drug utilisation sub-committee (DUSC)*. 2023: Canberra.
4. Camacho, X., et al., *Quality use of publicly subsidised tapentadol in Australia: a population-based analysis.* Intern Med J, 2024.
5. Seppala, L.J., et al., *Fall-Risk-Increasing Drugs: A Systematic Review and Meta-analysis: III. Others.* J Am Med Dir Assoc, 2018. **19**(4): p. 372.e1-372.e8.
6. Yoshikawa, A., et al., *Opioid Use and the Risk of Falls, Fall Injuries and Fractures among Older Adults: A Systematic Review and Meta-Analysis.* J Gerontol A Biol Sci Med Sci, 2020. **75**(10): p. 1989-1995.
7. Söderberg, K.C., L. Laflamme, and J. Möller, *Newly initiated opioid treatment and the risk of fall-related injuries. A nationwide, register-based, case-crossover study in Sweden.* CNS Drugs, 2013. **27**(2): p. 155-61.
8. Modén, B., et al., *Psychotropic drugs and falling accidents among the elderly: a nested case control study in the whole population of Scania, Sweden.* J Epidemiol Community Health, 2010. **64**(5): p. 440-6.
9. Hopkins, R.E., et al., *Age-Related Risk of Serious Fall Events and Opioid Analgesic Use.* JAMA Intern Med, 2024. **184**(4): p. 394-401.
10. Channell, J.S. and S. Schug, *Toxicity of tapentadol: a systematic review.* Pain Manag, 2018. **8**(5): p. 327-339.
11. Faria, J., et al., *Comparative pharmacology and toxicology of tramadol and tapentadol.* Eur J Pain, 2018. **22**(5): p. 827-844.
12. *Australian Product Information - Palexia SR (Tapentadol (as hydrochloride)) sustained release tablets*, in *in TGA Product and Consumer Medicine Information*. 2024.
13. Baron, R., et al., *Tapentadol Prolonged Release for Chronic Pain: A Review of Clinical Trials and 5 Years of Routine Clinical Practice Data.* Pain Pract, 2017. **17**(5): p. 678-700.
14. Murphy, D.L., et al., *Comparative Rates of Mortality and Serious Adverse Effects Among Commonly Prescribed Opioid Analgesics.* Drug Saf, 2018. **41**(8): p. 787-795.
15. Liu, S., et al., *Tapentadol Versus Oxycodone for Opioid-Related Adverse Drug Events and Clinical Outcomes After Inpatient Surgery.* J Pain, 2024. **25**(2): p. 466-475.
16. Deeks, E.D., *Tapentadol Prolonged Release: A Review in Pain Management.* Drugs, 2018. **78**(17): p. 1805-1816.
17. Etropolski, M., et al., *Safety and Tolerability of Tapentadol Extended Release in Moderate to Severe Chronic Osteoarthritis or Low Back Pain Management: Pooled Analysis of Randomized Controlled Trials.* Advances in Therapy, 2014. **31**(6): p. 604-620.
18. Deyo, R.A., et al., *Opioids for back pain patients: primary care prescribing patterns and use of services.* J Am Board Fam Med, 2011. **24**(6): p. 717-27.
19. Zoega, H., et al., *The Medicines Intelligence Data Platform: A population-based data resource from New South Wales, Australia.* medRxiv, 2024: p. 2024.04.29.24306520.
20. Ou, L.B., et al., *Tramadol versus codeine and the short-term risk of cardiovascular events in patients with non-cancer pain: A population-based cohort study.* Br J Clin Pharmacol, 2022. **88**(4): p. 1824-1834.
21. Nielsen, S., et al., *A synthesis of oral morphine equivalents (OME) for opioid utilisation studies.* Pharmacoepidemiol Drug Saf, 2016. **25**(6): p. 733-7.

22. Manchikanti, L., et al., *Responsible, Safe, and Effective Prescription of Opioids for Chronic Non-Cancer Pain: American Society of Interventional Pain Physicians (ASIPP) Guidelines.* Pain Physician, 2017. **20**(2s): p. S3-s92.
23. Gong, J., et al., *Persistent opioid use and opioid-related harm after hospital admissions for surgery and trauma in New Zealand: a population-based cohort study.* BMJ Open, 2021. **11**(1): p. e044493.
24. Gillies, M.B., et al., *Long-term prescribed opioid use after hospitalization or emergency department presentation among opioid naïve adults (2014-2020)-A population-based descriptive cohort study.* Br J Clin Pharmacol, 2024.
25. Hilmer, S.N., et al., *A drug burden index to define the functional burden of medications in older people.* Arch Intern Med, 2007. **167**(8): p. 781-7.
26. Langeard, A., et al., *Association between Statin Use and Balance in Older Adults.* Int J Environ Res Public Health, 2020. **17**(13).
27. Osman, A., et al., *Fall-Risk-Increasing Drugs and Gait Performance in Community-Dwelling Older Adults: Exploratory Results from the Gait and Brain Study.* Drugs Aging, 2023. **40**(8): p. 721-730.
28. Ge, J., et al., *Amelioration of gait and balance disorders by rosuvastatin is associated with changes in cerebrovascular reactivity in older patients with hypertensive treatment.* Hypertens Res, 2024.
29. de Vries, M., et al., *Fall-Risk-Increasing Drugs: A Systematic Review and Meta-Analysis: I. Cardiovascular Drugs.* J Am Med Dir Assoc, 2018. **19**(4): p. 371.e1-371.e9.
30. Gilbert, T., et al., *Development and validation of a Hospital Frailty Risk Score focusing on older people in acute care settings using electronic hospital records: an observational study.* Lancet, 2018. **391**(10132): p. 1775-1782.
31. Street, A., et al., *The use of linked routine data to optimise calculation of the Hospital Frailty Risk Score on the basis of previous hospital admissions: a retrospective observational cohort study.* Lancet Healthy Longev, 2021. **2**(3): p. e154-e162.
32. Langan, S.M., et al., *The reporting of studies conducted using observational routinely collected health data statement for pharmacoepidemiology (RECORD-PE).* Bmj, 2018. **363**: p. k3532.
33. Kotecha, D., et al., *CODE-EHR best-practice framework for the use of structured electronic health-care records in clinical research.* Lancet Digit Health, 2022. **4**(10): p. e757-e764.
34. Normand, S.T., et al., *Validating recommendations for coronary angiography following acute myocardial infarction in the elderly: a matched analysis using propensity scores.* J Clin Epidemiol, 2001. **54**(4): p. 387-98.
