# Supplementary material for: Short-term risk of falls among initiators of controlled-release tapentadol versus oxycodone in New South Wales between 2014 and 2020: A population-based retrospective cohort study
Source: PLoS Med. 2026 Aug 11;23(8):e1004950. doi: 10.1371/journal.pmed.1004950 (PMC13460682; doi:10.1371/journal.pmed.1004950)
Supplement: Supplementary file 27 [file pmed.1004950.s029.pdf]

30 April 2026
Dr Andreia Cunha
Senior Editor, PLOS Medicine

Dear Dr Cunha,

Re: Author response to reviewer comments on manuscript #PMEDICINE-D-26-00438R1 "Short-term risk of falls among initiators of controlled-release tapentadol versus oxycodone: A population-based cohort study"

Thank you for the opportunity to respond to reviewer comments. We are grateful for their insightful feedback. We have carefully considered their comments and revised the manuscript accordingly.

Please find our responses to each of the reviewers' comments outlined in the table below. Responses to editorial and technical comments are outlined in subsequent tables. Our revised manuscript is enclosed. Please note that the page and line numbers in our responses relate to those in the clean version of the manuscript.

Kind regards,

Ximena Camacho on behalf of the authors

Reviewer comments	Author response	Page / line number
**Reviewer #1**		
Main feedback<br>The primary outcome is a composite of falls associated with ED visit, hospitalisation, or death. The problem with composite outcomes is that they implicitly attach equal importance to each constituent outcome. This is rarely the case. Here, falls associated with death are clearly more significant than falls associated with hospital visits. As presented, it is not possible to gauge the effect in each of the sub-outcomes. Win ratios are a way of combining multiple outcomes in a hierarchy to infer overall clinical superiority whilst respecting that outcomes are ordered by importance. I feel a win ratio analysis would have been superior to a composite outcome analysis. I am not proposing that the authors should re-analyse all their data. I do think at the very least, however, that the authors should report the event rates and relative risk for each of the three constituent components in the composite separately in the main analysis population. I.e. what is the relative risk of falls associated with death; hospitalisation; and ED presentation? It is certainly not required that each of these comparisons would be significant (although I suspect some might be). I would expect these three effects to point in the same direction, I.e. tapentadol	We agree that, in many contexts, composite outcomes can obscure differences in the clinical importance of individual components. This is particularly the case for composite outcomes that include disparate endpoints with disparate impacts on the person. Our rationale for using a composite outcome was that all three components – ED presentation, hospitalisation, and death – represent consequences of the same precipitating event, namely a fall. While the severity of these outcomes clearly differs, we considered progression to more serious outcomes to be driven largely by circumstances surrounding the fall itself (for example, mechanism, height, or surface landed upon) rather than by fundamentally different causal processes. On this basis, we feel that the composite outcome appropriately captured overall fall-related harm. For this reason, we believe the primary composite outcome remains appropriate.<br><br>However, we have added event rates and relative risks for each component of our composite measure to the supplementary files (S19 Table).<br><br>To clarify this for the reader, we have added the following text to the Methods:<br>"*Finally, as our primary outcome is a composite, we calculated event rates and relative risks of falls separately for each constituent component (ED presentation, hospital admission, and death).*"<br><br>We added the following text to the Results:<br>"*Results of our sensitivity analyses were consistent with those of our primary analysis (S9 Table-S13 Table; S19 Table).*"	Lines 326-328, page 13<br><br>Lines 415-416, page 27<br><br>Lines 430-431, page 27<br><br><u>Supplementary files</u><br>S19 table

Reviewer comments	Author response	Page / line number
providing benefit. This could be an analysis for the supplement.	"*Nearly everyone experiencing a fall presented to hospital or emergency department settings; very few falls resulted in death (S19 Table).*"	
Table 1 is very informative, very thorough, and shows how well matching has performed. Would it be fair of me to suggest that inverse probability weighting would tend to be preferred over propensity score matching these days? IPW would have had the benefit of using all the patients, rather than the matchable subset. However, I suspect that since the sample size of the presented analysis is so large, and the matching so comprehensive, that I'd expect the inferences and conclusions to be the same.	Broadly speaking, propensity score matching and weighting target slightly different estimands. Matching generally permits estimation of the average treatment effect on the treated (ATT); weighting permits estimation of the average treatment effect (ATE) (although can also estimate the ATT). Because our study question was focused on the effect of tapentadol (SR) in people who were treated, our estimand of interest was the ATT.<br><br>The reviewer is correct that IPW would have allowed us to use all the patients in the analysis rather than the matchable individuals. However, when designing the study, we deliberately chose to employ a matched approach (in which we matched on calendar month/year of initiation as well as propensity score), as it facilitated estimation of the ATT and allowed us to properly account for changes in underling trends in opioid use as well as changes in opioid prescribing policies that came into effect over our study period.<br><br>This was outlined in the Methods (lines 281-285, pages 11-12), but we have also clarified this in the Discussion as follows:<br>"*We used exact and propensity score matching rather than other approaches such as inverse probability weighting or stratification because it better accounted for changes over time in opioid use and prescribing policies during the study period*."<br><br>We agree with the reviewer that an IPW approach would likely have resulted in very similar findings, given the high match rate and low rate of subject loss.	Lines 474-476, page 29

Reviewer comments	Author response	Page / line number
Minor feedback<br>In the Results section, you say there was a difference in non-study opioid use. Please add a few words to say in which direction the differences were. I.e. oxycodone CR patients were more likely to also receive immediate release oxycodone but less likely to receive codeine and tramadol.	We have added the following text to the Results section: "*People initiating oxycodone (CR) were more likely to be dispensed immediate-release oxycodone and less likely to receive codeine and tramadol compared to people initiating tapentadol (SR) (Table 2)*."	Lines 355-357, page 19
I looked for typos but could not find any!	We appreciate the thorough review!	-
I know this research is not about addiction but the main reason I have heard of oxycodone is the opioids addiction crisis with OxyContin in USA. Is tapentadol similarly implicated? I expected a sentence or two in the Discussion, particularly in light of the high NNT presented. In such a situation, with an otherwise benign and cheap treatment, you might treat despite the high NNT. But of course that is not the case here. The effect you want and the effect you don't want are two sides of the same coin.	There is evidence that tapentadol has a lower addictive potential than oxycodone because of lower affinity for mu-opioid receptors. However, we are uncomfortable going beyond our data and cautious of including text that might be seen as promotional or giving prescribing advice in a paper that is otherwise narrowly focused. We agree that the NNT values are generally high; however, in the case of people aged ≥80 years who are opioid-naïve, the NNT are lower and fall into the range that influences prescribing decisions (28 days post-initiation: NNT 97, 95% CI 67-189). We are therefore more comfortable commenting on this specific group and have added text to the Discussion as follows:<br>"*Indeed, this may be most pertinent for opioid-naïve people aged 80 years and older, where less serious fall events may affect confidence in walking, subsequently reducing mobility and quality of life and increasing social costs; tapentadol (SR) may be preferable over oxycodone (CR) in this group*."<br>"*In a broader context, however, falls are only one of many aspects that require consideration when choosing to prescribe opioids*."	Lines 511-514, page 31<br>Lines 516-517, page 31
**Reviewer #2**		

Reviewer comments	Author response	Page / line number
This is a very interesting paper with thorough methodology investigating a highly relevant research question, from both a clinical and public health perspective. The authors have made a tremendous attempt to showcase the robustness of their analyses and results. I have some minor suggestions for improving the manuscript.	Thank you, we appreciate the positive feedback.	-
Abstract<br>The abstract would benefit from additional rationale for the study.	We have clarified the 'Background' section of the Abstract as follows:<br>"*Clinical trials comparing sustained-release (SR) tapentadol with controlled-release (CR) oxycodone have suggested that tapentadol (SR) may be associated with fewer nervous system side effects. However, it remains important to evaluate the relative safety of these agents in real-world clinical settings. Given the potential severity and economic burden of falls, together with their established association with opioid use, we compared the short-term risk of falls following initiation of tapentadol (SR) versus oxycodone (CR) in routine clinical practice.*"	Lines 61-66, page 3
Introduction<br>The order of the text in the introduction could be improved by expanding a bit on the tolerability. Like is it varying for chronic or new users and whether serious adverse effects are larger/more common in the initiation phase, and what is the pharmacokinetic rationale for any differences between the medications.	Tolerability was not an outcome of interest, so we did not address it in detail in the Introduction. Nonetheless, we have added the following text to the Introduction:<br>"*This may be due to pharmacological differences (e.g., comparatively lower opioid potency or NRI effect with tapentadol (SR)) [reference 4].*"<br><br>Additionally, both study opioids are controlled release formulations with terminal half-lives of 4 – 6 hours, so pharmacokinetics are not likely to explain the observed differences in fall rates.	Lines 143-144, page 6
The introduction would also benefit from clarifying a more detailed description of the	Tapentadol (SR) and oxycodone (CR) are both subsidised by the Australian Pharmaceutical Benefits Scheme (Australia's national	Lines 210-213, page 9

Reviewer comments	Author response	Page / line number
indication for the two medications compared and particular if there are the same indication and contraindications.	public medicines subsidy program). Both medicines have identical listed indications for use in patients with chronic severe pain requiring daily, continuous use of long-term opioids. We have added the following text to the Methods (with supporting references) to clarify this:<br>“*Both tapentadol (SR) and oxycodone (CR) are subsidised by the Pharmaceutical Benefits Scheme (PBS; Australia’s public medicines subsidy program) and have identical listed indications for use in patients with chronic severe pain requiring daily, continuous, long-term opioid treatment [new references 21-23].*”	
The introduction is silent about the perspective of type of falls, that is later raised as an interesting perspective in the results.	We agree that the Introduction would benefit from additional discussion on the significance of falls. The following text has been added:<br>“*In light of clinical trial evidence demonstrating that tapentadol (SR) and oxycodone (CR) achieve comparable analgesic effects and improvements in quality of life [references 4,9], it is relevant to compare the safety of these products in real-world settings.*”<br>“*Falls can have devastating consequences, including mortality and life-long impacts on independence and mobility, particularly among older people [reference 14].*”	Lines 152-154, page 6<br>Lines 157-158, page 6
Methods<br>The data is from 2020, has there been any changes in the treatment guidelines since then?	The data reviewed here cover the period 1 September 2014 to 3 December 2020. Changes to prescribing restrictions for PBS subsidised medicines were introduced in June 2020, including requirements for Department of Health authorisation for repeats, second clinical opinions to confirm the need for extended treatment, and reduced pack sizes. These reforms therefore potentially affect the final six months of the study period. However, we have previously reported[1], using time-series analyses, that these changes were associated with only a modest reduction of approximately 2–3% in the volume of controlled release opioids dispensed in Australia	-

Reviewer comments	Author response	Page / line number
	(measured in oral morphine equivalents). We therefore consider it unlikely that these reforms materially biased the estimates of relative falls risk reported in this study.	
Patient and public involvement<br>Given the research question, is there a reason for not including stakeholders from e.g. the clinicians or health care planers?	We agree that patient and public involvement is important. One of our authors (SM) provides a strong consumer perspective with lived experience of the topic. Two authors (JB and DH) are experienced clinicians with direct experience of prescribing opioids. Three authors (NP, DH, SAP) have extensive experience in the fields of medicines regulation and subsidy and two are involved directly in the government committees that oversee these activities. We will also present the outcomes of this research prior to publication with representatives of the Pharmaceutical Benefits Advisory Committee (an independent body appointed by the Australian Government to recommend new medicines for subsidy on the Pharmaceutical Benefits Scheme) and the Therapeutic Goods Administration (Australia's medicines regulator).	-
Was repeated events of falls considered?	In this study, we were interested in assessing only the first fall event that occurred in the four weeks following initiation of study opioids. Whilst we agree with the reviewer that repeated falls events are important to investigate, such an analysis would require a different study design and analytical approach, as well as a much longer follow-up duration.<br>We have added text to the Discussion acknowledging this as a potential limitation of the present study and highlighting the need for research with longer follow-up as follows:<br>"*Seventh, we only assessed the first fall in the four weeks following opioid initiation; future studies with longer follow-up duration should investigate repeated fall events.*"	Lines 498-500, page 30

Reviewer comments	Author response	Page / line number
There exclusion criteria regarding "the included individuals should have no dispensation history for the last two years" seems to not align with the criteria that the included individuals only had to live in NWS for the year prior to initiation. If not living in NWS for the whole 2 year period, how can you ascertain the dispensation history?	We apologise for the lack of clarity here. The Pharmaceutical Benefits Scheme is a national program, and people retain eligibility for supply of subsidised medicines when moving between Australian states and territories.<br>The two-year lookback window for dispensing history was used to ensure sufficient data availability for ascertaining opioid initiation. As outlined in the Methods, we required a 365-day period free from study opioid dispensing for people to qualify for cohort entry. We therefore needed to look further back to ensure that people were represented in the data during the 365-day lookback period.<br>The New South Wales (NSW) data was used to determine comorbidities and study endpoints. We used a one-year lookback window to establish stable geographic residency and eligibility to receive state-based health services (i.e., hospitals and emergency services).<br>We have added a clarification to the Methods to this effect:<br>"*The Pharmaceutical Benefits Scheme is a national program and people retain eligibility when moving between Australian states and territories; hence, the lookback period for dispensing history exceeds the requirement for residence in NSW.*"	Lines 229-232, page 9
A graphical presentation of the different time periods would aid readers to follow the different periods of interest for exposure and outcomes.	A graphical depiction of the study design was presented in Supplementary Figure 1. However, we appreciate that this may not have been clear to the reader. We have therefore added the following sentence to the Methods section: "*A graphical presentation of the study design is presented in S1 Figure.*"	Line 229 page 9
Are there any previous studies on the internal validity of the outcome, falls, based on the Medintel database?	We did not conduct any direct validation of the coded diagnostic outcomes as this would require retrieval of primary records (a major undertaking). There are also no previous studies validating this outcome in Australia. However, based on input from our clinical co-authors, we believe that the identification of a fall leading to an ED	-

Reviewer comments	Author response	Page / line number
	visit, hospital admission, or death is not prone to a high degree of misclassification. Indeed, a recent systematic review assessing the validity and reliability of ICD-10 external cause of injury codes (including falls) found these codes to have 91.6% specificity, 61.6% sensitivity, 74.9% positive predictive value, and 80.2% negative predictive value[2].<br>The endpoint definitions we have used in this study are those used previously, including a recent report we published on opioid-related falls (Hopkins RE et al. JAMA Intern Med 2024;184:394-401)[3]. This is reference 14 in the current paper.	
It is not clear to me what is meant by "we included fall-related diagnosis codes in any position", what is position referring to? And is there a code for falls occurring in the hospital setting, how was that ascertained?	We apologise for the lack of clarity. In the NSW hospital data, diagnoses are coded according to the International Classification of Diseases, 10th Revision (Australian Modification). For each episode of care, a principal diagnosis and up to 50 additional (also termed secondary) diagnoses may be recorded. However, in practice, the average number of entered codes is around three. For our study, an ICD-10-AM code representing a fall recorded in any of the diagnosis fields (i.e., either principal or additional/secondary) was classified as an outcome. The technical definition of falls (including ascertainment of out-of-hospital falls) is outlined in S4 Table.<br><br>For each coded diagnosis recorded during the hospital stay, data is also collected on the onset of the condition relative to the start of the episode of care. Any events that occur during the course of a hospital stay are classified as having a condition onset during the episode of care. To ensure that we only captured out-of-hospital falls, we therefore excluded any fall events where the fall had a condition onset flag indicating an in-hospital event.<br><br>We have amended the text in the Methods as follows:	Lines 244-248, page 10

Reviewer comments	Author response	Page / line number
	"We ascertained falls from the hospital data using International Classification of Diseases (ICD), 10th edition, Australian Modification (ICD-10-AM)~~; we included fall-related diagnosis codes in any position, where the fall did not occur in hospital~~. A diagnosis code representing a fall recorded in any data field (i.e., either principal or additional diagnosis) was classified as an outcome. We excluded falls that had a flag indicating they occurred in a hospital setting."	
Please provide references for the covariates.	Detailed technical definitions of all covariates (including references) have been provided in S4 Table. However, on review we noted that references for many of the comorbidities were only presented in the Supplement; we have therefore added those references to the main text (new references 27-31). We have also aligned references between the supplementary materials and main text.	Line 258 Page 11
What was the rationale for the different lengths of reference for the non-study opioids (90 days), FRIDs (30 days) and no other dispensations in the year (365 days)?	These lookback intervals were based on pragmatic clinical considerations.<br>For falls-risk medicines, we were interested in whether people were likely to be exposed around the time of a fall, so a recent prescription (within previous 30 days) was the relevant measure of exposure.<br>For recent non-study opioids, we used a longer lookback to increase the sensitivity of exposure detection, particularly if a previous supply was used intermittently and would not be captured by a shorter reference period (e.g., 30-day lookback).<br>In database studies such as ours, new use is defined by a dispensing for the medicine(s) of interest following an arbitrary period with no prescriptions (akin to a wash-out period in clinical trials). The length of the period is one that is clinically meaningful for the medicine in question. A 365-day lookback is commonly used in pharmacoepidemiological studies, particularly those assessing opioids.	-

Reviewer comments	Author response	Page / line number
Was there any information on the type of injuries sustained due to the fall?	We ascertained falls using data from hospital, emergency department (ED), and death records. Capture of injuries resulting from falls varies across each source:<br>• For falls captured in hospital data, information on injuries (particularly those requiring treatment) would have been captured using corresponding ICD-10-AM codes on the same record as the fall.<br>• The death data captures the underlying cause and up to 19 other causes of death. For falls resulting in death, it is possible that injuries may have also been recorded on the death record.<br>• The emergency department data only captures a single coded reason for visit (coded using one of three classifications: ICD Ninth Revision, Clinical Modification; ICD-10-AM; and Systematized Medical Nomenclature for Medicine-Clinical Terminology [SNOMED-CT]). These records will therefore mainly capture the occurrence of a fall event and not necessarily any resulting injuries. Although there are some SNOMED codes that capture both a fall and injury together, not all falls will be coded according to this classification.<br><br>Given the inconsistent capture of fall-related injuries across data sources, and in light of our focus on the occurrence of falls more generally, we did not explore the types of injuries sustained from falls occurring during follow-up. Although this would be an interesting question to examine in future work, it would entail identification of a broad range of specific injuries (e.g., various limb fractures, rib fractures, skill fractures, etc.) and is beyond the scope of the current study.	-
And for those with a previous surgery (in the last 30 days), any information on type of	We agree that the nature of recent surgery and the associated length of hospital stay are relevant as they may be associated with increased falls tendency. In addition, surgery often leads to initiation of short-	-

Reviewer comments	Author response	Page / line number
surgical procedure, reason for the surgery or length of stay in hospital?	term opioid treatment, which can sometimes be prolonged. Recent surgery is therefore a potential confounder, and was included in the propensity score. We found that recent surgery was more common in the unmatched oxycodone cohort (standardised difference [SD] 0.14) but the SD was zero after matching. It is therefore unlikely that the nature of the surgical procedure and associated length of stay differed between the two cohorts to a degree that would bias our effect size estimates.	
Results<br>Clearly written and easy to follow. I only have a suggestion, to place the information on the matching to the method section.	We described the matching techniques in the Methods section (lines 278-281, page 11) but felt we should also include some detail in the Results to aid interpretation.	-
For the residual confounding it would be interesting to read what type of residual confounding that worries the authors.	Our principal concern is that new users of oxycodone are ‘sicker’ than new users of tapentadol. This is because of some prescriber perceptions that oxycodone is a ‘stronger’ analgesic, with a greater tendency to abuse, and should be reserved for more severe forms of pain and serious illness. The tapentadol users may represent a ‘healthier cohort’ (comparatively). This is why we conducted a new user study, included a substantial list of potential confounders in the propensity scores, performed cohort restriction (according to prior and concurrent use of non-study opioids), and included sensitivity analyses and a negative control outcome.<br><br>We have added the following text to the ‘Strengths and limitations’ section of the Discussion to clarify this for the reader:<br>“*To mitigate the possibility that people prescribed tapentadol (SR) represent a comparatively ‘healthier’ cohort than people prescribed oxycodone (CR), we conducted a new user active comparator study, included a substantial list of potential confounders in the propensity score, performed cohort restriction (according to prior and concurrent*	Lines 469-474, page 29

Reviewer comments	Author response	Page / line number
	use of non-study opioids) and included a suite of sensitivity analyses and a negative control outcome."	
The negative control approach is a very good idea, however, the statistical power around the cataract surgeries are small in several instances. Why was cataract chosen, please provide some more short information about the rationale as cataract are normally performed under very strict pre-surgical conditions?	We acknowledge that there is uncertainty in the literature regarding the optimum choice of a negative control outcome or negative control exposure. The clinicians on our team judged that it was better to choose an outcome that they viewed as unrelated to the exposures of interest (recent initiation of tapentadol or oxycodone) than it was to choose a negative exposure control, as many medicines and procedures have been postulated to increase falls risks, particularly in older people. Having decided on using a negative control outcome, we chose cataract surgery, noting that both cataract and joint surgery are the most common elective procedures in older adults. Of the two, cataract surgery seemed the least likely to be associated with recent initiation of an opioid. We would expect to generate a null result if there was no residual confounding. We accept that the estimates of association are rather imprecise, but they are consistent with the results of our propensity score matched analyses, restricted cohort analyses, and sensitivity analyses in supporting our belief that the final results are not seriously confounded.	-
I am a bit uncertain of the "Patient and Public Involvement" section, to me it is quite narrow to confine in the feed-back from one end-user, that is also included in the project team, a colleague and author of the paper. There is however no indication on attempting to include stakeholders from the health care system planers or clinicians which, given the research question would be equally important. The section I do not think strengthen the paper and the statements are more for dissemination of	We addressed this above and repeat our comment here. One of our authors (SM) provides a strong consumer perspective with lived experience of the topic. Two authors (JB and DH) are experienced clinicians with direct experience of prescribing opioids. Three authors (NP, DH, SAP) have extensive experience in the fields of medicines regulation and subsidisation and two are involved directly in the government committees that oversee these activities.<br><br>Many major funding bodies and journals are actively encouraging the involvement of consumers in the conduct of research. We feel it is important to highlight the influence and impact of our consumer	-

Reviewer comments	Author response	Page / line number
findings from a project, not for this specific scientific manuscript.	collaborator on this study and have therefore outlined their contributions in the Methods section.	
**Reviewer # 3**		
This is an interesting paper that adds to the falls literature, particularly in relation to how opioid use influences an individual's risk of falling. The paper is well written, and the analysis undertaken is appropriate. I only some minor comments for consideration:	Thank you, we appreciate this positive feedback.	-
Page 3: Some additional background information about the link between falls and opioid use would be helpful to frame the study context.	As noted above in response to Reviewer #2, we have amended the Background section in the Abstract to better frame the study context: "*Clinical trials comparing sustained-release (SR) tapentadol with controlled-release (CR) oxycodone have suggested that tapentadol (SR) may be associated with fewer nervous system side effects. However, it remains important to evaluate the relative safety of these agents in real-world clinical settings. Given the potential severity and economic burden of falls, together with their established association with opioid use, we compared the short-term risk of falls following initiation of tapentadol (SR) versus oxycodone (CR) in routine clinical practice.*"	Lines 61-66, page 3
Page 3: Avoid using the term 'suffer' as it reinforces stigma and is deficit-framing. Consider using the term 'experienced' instead.	We have modified the text accordingly: "*Within 28 days of initiation, 652 (0.6%) oxycodone (CR) initiators and 457 (0.4%) tapentadol initiators experienced a fall.*"	Lines 80-81, page 3
Page 5: Consider making it clearer as part of the research aim / objective that the analysis included examining the risks of falls in individuals who were on non-study opioids.	We have amended the text in the Introduction as follows: "*…we aimed to quantify the short-term risk of falls resulting in emergency department (ED) presentations, hospital admissions, or death, following initiation of tapentadol (SR) compared to initiation of*	Lines 160-163, page 7

Reviewer comments	Author response	Page / line number
	oxycodone (CR) in real-world settings that included people who concurrently used multiple opioids."	
Page 5: Avoid using ageist language e.g. 'elderly'. The term 'older adults' or 'older people' are preferred.	We have modified the text accordingly: "*…this risk is highest in the first 7 days following initiation, in older people, and persists through the first 28 days of use.*"	Lines 155-157, page 6
Page 5: Given that several subgroup and sensitivity analyses were undertaken (which are appropriate), it may be helpful to state these as explicit research aims or objectives in the introduction.	Thank you. Our aim was not to estimate the risk of falls with non-study opioids. Instead, we were concerned that their concurrent use could confound or modify the estimated risk of falls with the study drugs. For this reason, we included use of non-study opioids in the last 90 days in the propensity score analyses (confounding), performed restricted cohort analysis (with and without recent use of non-study opioids), and examined potential effect modification by concurrent initiation of a non-study opioid.<br>These subgroup and sensitivity analyses were therefore performed to adjust for confounding and effect modification for the primary outcomes, rather than as explicit research aims / objectives.<br>In response, we have made this clearer by amending text in the Methods as follows:<br>"*To assess potential confounding from recent exposure to non-study opioids, we conducted stratified analyses examining the risk of falls among people with and without non-study opioid exposure in the 90 days before treatment initiation.*"<br>"*We conducted several sensitivity analyses to assess potential confounding or effect modification of concurrent use of non-study opioids on the risk of falls.*"	Lines 302-304, page 12<br>Lines 307-308, page 13
Page 9: The way in which the history of falls variable was determined as a confounder may require some explanation as the timeframe of 2 years to 60 days is slightly unusual. This may	Thank you for raising this important point.<br>We excluded individuals with falls occurring 0-60 days prior to study opioid initiation to minimise the risk of protopathic bias. In contrast, falls occurring 61 days to 2 years prior to initiation were treated as a	Lines 492-497, page 30<br>Lines 500-502, page 30

Reviewer comments	Author response	Page / line number
explain the relatively low proportion of individuals with a previous history of falls (Table 1) which differs markedly to the known prevalence of falls in the general community-dwelling middle-aged (18-25%) and older adult populations (30-35%).	confounder. (This is described in the Methods section [lines 227-229, page 9; lines 262-263, page 11]).<br>We did not have access to self-report or primary care records, which would have captured falls not resulting in ED attendance or hospital admission. This explains the low falls rate documented in our study. The degree of under-ascertainment should be unrelated to the treatment, so it should not impact the internal validity of our findings. However, it may affect their generalisability, and we have added this as a potential limitation of the study as follows:<br>"*Fifth, we were unable to completely ascertain previous history of falls; we did not have access to self-report or primary care records, which would have captured falls not resulting in ED attendance or hospital admission. This may affect the generalisability of our findings. However, the degree of under-ascertainment should be unrelated to the treatment, and thus should not affect the internal validity of the results.*"<br>We have also clarified the focus of our study on more severe falls:<br>"*Lastly, our study endpoint captures severe falls resulting in contact with the healthcare system and does not capture the full health and social impact of all falls that may have occurred following study opioid initiation.*"	
Page 12: Providing a little more detail about the study cohort may help readers judge whether the sample is representative of the broader population using opioids.	Australia has a universal health care system called Medicare that provides access to subsidised health care services for all Australian citizens, permanent residents, and citizens from countries that have reciprocal health care arrangements with Australia (New Zealand, the United Kingdom, Ireland, and some European countries). The Australian Pharmaceutical Benefits Scheme (PBS) is a national program that provides subsidised medication to people who are eligible for Medicare. The sample comprises all Medicare-eligible people resident in NSW during the study period (and is therefore representative of all people eligible for subsidised prescription medicines).	Lines 182-190, pages 7-8

Reviewer comments	Author response	Page / line number
	The study cohort is the subset of PBS-eligible adults (aged 18 years and over) who received an initial prescription for tapentadol (SR), matched to adults receiving an initial prescription for oxycodone (CR). Although doctors can write private prescriptions, this will only apply to a small group of PBS-eligible patients as they will be required to pay the full cost of the medicine.<br>This study faces two main threats to generalisability. The first has already been identified by the reviewer – we captured only severe falls resulting in contact with the healthcare system. The second is that we studied only the sustained-release form of tapentadol; the immediate-release formulation is only available by private prescription. We have discussed both issues in the Methods and Discussion.<br>In order to help readers evaluate the representativeness of the sample, we have expanded the text describing the PBS-eligible population as outlined below. We feel that the other representativeness issues are covered adequately in the existing text.<br>"*Australia has a universal health care system (Medicare) providing subsidised access to health services, including prescription medicines, and hospital care to all Australian citizens, and permanent residents, and citizens of countries with reciprocal arrangements (e.g., New Zealand, United Kingdom, Ireland, and some European countries). The Pharmaceutical Benefits Scheme (PBS) is a national program that provides access to subsidised prescription medicines for all Medicare-eligible people… Briefly, the platform comprises longitudinal, linked population-level data for all adult Medicare-eligible residents of New South Wales (NSW), Australia's most populous state (8.5 million), between 2005 and 2020.*"	
Page 16: While the authors acknowledge the study's limitations, it may also be important to note that some variables may be subject to	We agree, and have added the following text to the limitations section in the Discussion:	Lines 497-498, page 30

Reviewer comments	Author response	Page / line number
misclassification or incomplete capture (e.g., history of falls).	"*Sixth, some variables may be subject to misclassification or incomplete capture (e.g., history of falls).*"	
**Reviewer #4**		
This manuscript is a carefully written report of an interrogation of a large dataset that has examined the research question.	Thank you, we appreciate this positive feedback.	-
There are many strengths to the approach, including use of propensity score matching involving multiple variables that may plausibly have an impact on the outcome, a large sample size, and a relatively firm composite outcome for falls based on fall related emergency department presentations, hospitalisation or death due to falls. The limitations of the approach mainly centre on what data were available for inclusion in the analyses for both the outcome (falls that did not result in hospital presentation or death but may otherwise have had a substantial impact on the life of the individual were not recorded) and predictor variables available to be used in propensity score matching. An important issue not discussed is when the decision regarding the period of data extraction (Sept 2014 to Dec 2020) was selected relative to when the authors had access to the data. The ability to select time periods of data collection after having viewed the data can increase the risk of Type 1 errors (see Journal of Physiotherapy. 2023 Oct 1;69(4):278-82). Also, an explanation	Thank you for these comments. Some of the issues raised have been covered in the responses above.<br><br>We acknowledge that the study endpoint does not capture the full health and social impact of all falls and have added text to the limitations section of the Discussion to this effect: "*Lastly, our study endpoint captures severe falls resulting in contact with the healthcare system and does not capture the full health and social impact of all falls that may have occurred following study opioid initiation.*"<br><br>Regarding the dates covered by the data, we are limited by data access in Australia, which does not provide academic access to continuously updated linked health data. We leveraged data from a custom database, the Medicines Intelligence Data Platform, to conduct our study. This resource comprises all Medicare-eligible adults who resided in New South Wales at any time between 2005-2020. For each person in the cohort, records on health service utilisation (including dispensing of prescription medicines, hospitalisations, ED visits, and death records) were available up to 31 March 2022. At the time of platform establishment, this reflected the most recent data available to researchers in Australia[4]. As this is not a government-maintained database it will only be updated when permission from the various data custodians has been received.	Lines 500-502, page 30

Reviewer comments	Author response	Page / line number
of why data was not collected after Dec 2020 is also warranted given it is now 2026.	Importantly, the hypotheses being tested here were not based on prior scrutiny of the data and the analyses were run on the complete dataset available to us and not in a sub-sample. Our first sight of these results was when the analytic output was generated.	
The results of the analysis indicated a large NNT (in excess of 500) to prevent one fall due in part to the infrequency of the outcome being measured. The implications of this (should Tapentadol (SR) be prescribed in preference to Oxycodone?) would benefit from more explicit discussion and analysis, indeed an economic evaluation from the government (as social health care insurer in Australia) perspective based on the evidence presented in this study would really elevate this manuscript.	In response to Reviewer #1, we expressed concern about providing prescribing advice that goes beyond our data, which are limited to serious falls. For this endpoint, the average NNT values are high, and on their own might not lead to changes in prescribing. However, against a background of better tolerability (seen in the randomised trials comparing the study opioids), the high NNT might move the needle towards choosing tapentadol (SR) over oxycodone (CR).<br>We are more comfortable commenting on older people (≥80 years) who are opioid-naïve. In this group, the NNT values were closer to 100 (or less), in a range that typically influences treatment choices when the outcome is serious (as are falls in this age group). Furthermore, as we noted in our responses above, the focus on serious falls misses the potential impacts of lesser events that may occur in this older age group and which might cause loss of confidence in walking, reducing mobility with consequent quality of life and social costs.<br>We have therefore added the following text to the Discussion that reflects these potential benefits in older, frail patients:<br>"*Indeed, this may be most pertinent for opioid-naïve people aged 80 years and older, where less serious fall events may affect confidence in walking, subsequently reducing mobility and quality of life and increasing social costs; tapentadol (SR) may be preferable over oxycodone (CR) in this group.*"	Lines 511-514, page 31

General editorial requests

Editorial request	Author response	Page / line number
We ask every co-author listed on the manuscript to fill in a contributing author statement, making sure to declare all competing interests. If any of the co-authors have not filled in the statement, we will remind them to do so when the paper is revised. If all statements are not completed in a timely fashion this could hold up the re-review process. If new competing interests are declared later in the revision process, this may also hold up the submission. Should there be a problem getting one of your co-authors to fill in a statement we will be in contact. Please do not add or remove authors without first discussing this with the handling editor. You can see our competing interests policy here: http://journals.plos.org/plosmedicine/s/competing-interests.	Noted – thank you	
Please upload any figures associated with your paper as individual TIF or EPS files with 300dpi resolution at resubmission; please read our figure guidelines for more information on our requirements: http://journals.plos.org/plosmedicine/s/figures. While revising your submission, we strongly recommend that you use PLOS's NAAS tool (https://ngplosjournals.pagemajik.ai/artanalysis) to test your figure files. NAAS can convert your figure files to the TIFF file type and meet basic requirements (such as print size, resolution), or provide you with a report on issues that do not meet our requirements and that NAAS cannot fix.	NAAS-processed figures have been uploaded at resubmission.	-

Editorial request	Author response	Page / line number
After uploading your figures to PLOS's NAAS tool - https://ngplosjournals.pagemajik.ai/artanalysis, NAAS will process the files provided and display the results in the "Uploaded Files" section of the page as the processing is complete.<br>If the uploaded figures meet our requirements (or NAAS is able to fix the files to meet our requirements), the figure will be marked as "fixed" above. If NAAS is unable to fix the files, a red "failed" label will appear above.<br>When NAAS has confirmed that the figure files meet our requirements, please download the file via the download option, and include these NAAS processed figure files when submitting your revised manuscript.		
Please ensure that the paper adheres to the PLOS Data Availability Policy (see http://journals.plos.org/plosmedicine/s/data-availability), which requires that all data underlying the study's findings be provided in a repository or as Supporting Information. For data residing with a third party, authors are required to provide instructions with contact information (web or email address) for obtaining the data. Please note that a study author cannot be the contact person for the data. PLOS journals do not allow statements supported by "data not shown" or "unpublished results." For such statements, authors must provide supporting data or cite public sources that include it.	The data used to generate the results presented in this study resides with a third party; direct access is not permitted without the express permission of the approving data custodians and human research ethics committees. The Data Availability statement has been updated to include web and email addresses for inquiries related to data access.	Data Availability statement

**Editorial request**	**Author response**	**Page / line number**
Please remove the ethics statement from the Data Availability Statement. The ethics statement should be in the Methods.	The ethics statement has been moved to the Methods section.	Lines 329-332, page 14
We expect all researchers with submissions to PLOS in which author-generated code underpins the findings in the manuscript to make all author-generated code available without restrictions upon publication of the work. In cases where code is central to the manuscript, we may require the code to be made available as a condition of publication. Authors are responsible for ensuring that the code is reusable and well documented. Please make any custom code available, either as part of your data deposition or as a supplementary file. Please add a sentence to your data availability statement regarding any code used in the study, e.g. "The code used in the analysis is available from Github [URL] and archived in Zenodo [DOI link]" Please review our guidelines at https://journals.plos.org/plosmedicine/s/materials-software-and-code-sharing and ensure that your code is shared in a way that follows best practice and facilitates reproducibility and reuse. Because Github depositions can be readily changed or deleted, we encourage you to make a permanent DOI'd copy (e.g. in Zenodo) and provide the URL.	Code has been made available via GitHub. The Data Availability statement has been updated with the following text: "*The code used in the analysis is available from Github (https://github.com/Ximena-Camacho/Tapentadol_and_falls) and archived in Zenodo (https://doi.org/10.5281/zenodo.19626255).*"	Data Availability statement
Please ensure that all authors have completed the COI questionnaire. The disclaimers in the COI declaration and the funding statement should be set apart under the heading Disclaimer.	COI questionnaires have been completed	

Editorial request	Author response	Page / line number
Please provide a URL for each funder in the funding statement. Please ensure that the manuscript metadata matches statements in the manuscript.	URLs for each funder have been provided in the Funding statement.	Line 537, page 32<br>Line 540, page 32
We require that you include an email address for a contact for data requests. The contact may not be an author.	An email address for data requests has been provided in the Data Availability statement.	Line 568, page 33
Please use Vancouver style for references.	References have been formatted using Vancouver style.	Lines 578-778, pages 34-37
Please ensure that the study is reported according to the STROBE guideline and include the completed STROBE checklist as Supporting Information. When completing the checklist, please use section and paragraph numbers, rather than page numbers. Please add the following statement, or similar, to the Methods: "This study is reported as per STROBE guideline (S1 Checklist)."	See 'Observational Studies' below – this study has been reported according to the Reporting of studies Conducted using Observational Routinely collected health Data for Pharmaco-Epidemiology (RECORD-PE) statement, an extension of the STROBE guideline. The corresponding checklist is included as S1 Checklist.	Lines 169-171, page 7<br>S1 Checklist
Please include any additional checklists (e.g. GATHER, RECORD), as may be necessary.	As above, the RECORD-PE Checklist (which incorporates both STROBE and RECORD items) has been included.	S1 Checklist
FORMATTING - GENERAL		
Abstract: Please structure your abstract using the PLOS Medicine headings (Background, Methods and Findings, Conclusions). Please combine the Methods and Findings sections into one section.	Abstract has been formatted as instructed	Pages 3-4

Editorial request	Author response	Page / line number
In the last sentence of the Abstract Methods and Findings section, please describe the main limitation(s) of the study's methodology.	We have added the following text to the Methods and Findings section outlining the main study limitations:<br>"*The main limitations of this study were that we did not have data on immediate-release tapentadol, or on falls that did not result in emergency department presentations or hospitalisations but may have otherwise impacted independence and mobility.*"	Lines 91-93, page 4
At this stage, we ask that you include a short, non-technical Author Summary of your research to make findings accessible to a wide audience that includes both scientists and non-scientists. The Author Summary should immediately follow the Abstract in your revised manuscript. This text is subject to editorial change and should be distinct from the scientific abstract. Ideally each sub-heading should contain 2-3 single sentence, concise bullet points containing the most salient points from your study.	An Author Summary has been included in the manuscript file.	Page 5
* In the final bullet point of 'What Do These Findings Mean?', please include the main limitations of the study in non-technical language. Please see our author guidelines for more information: https://journals.plos.org/plosmedicine/s/revising-your-manuscript#loc-author-summary.	The main limitations have been discussed in the final bullet point of 'What Do These Findings Mean?'	Page 5, lines 130-133
Please express the main results with 95% CIs as well as p values. When reporting p values please report as p<0.001 and where higher as the exact p value p=0.002, for example. Throughout, suggest reporting	Results have been provided with 95% CIs. We have not included p-values as they do not add additional information to the more informative 95% CIs.	-

Editorial request	Author response	Page / line number
statistical information as follows to improve clarity for the reader "22% (95% CI [13%,28%]; p</=)". Please be sure to define all numerical values at first use.		
Please include page numbers and line numbers in the manuscript file. Use continuous line numbers (do not restart the numbering on each page).	Page and continuous line numbers are included in the manuscript file.	-
Please cite the reference numbers in square brackets. Citations should precede punctuation.	Reference numbers have been cited in square brackets and precede punctuation.	-
FIGURES AND TABLES		
Please provide titles and legends for all figures and tables (including those in Supporting Information files).	Titles and legends for each table and figure have been provided.	-
Please define all abbreviations used in each figure/table (including those in Supporting Information files).	All abbreviations in each figure and table have been defined.	-
Please consider avoiding the use of red and green in order to make your figure more accessible to those with color blindness.	No red or green in figures	-
SUPPLEMENTARY MATERIAL		
Please note that supplementary material will be posted as supplied by the authors. Therefore, please amend it according to the relevant comments outlined here.	-	-
Please cite your Supporting Information as outlined here: https://journals.plos.org/plosmedicine/s/supporting-information	Captions for all supporting information files have been included at the end of the manuscript document.	Lines 781-831, pages 38-39

**Editorial request**	**Author response**	**Page / line number**
REFERENCES		
PLOS uses the numbered citation (citation-sequence) method and first six authors, et al.	PLOS formatting has been applied to all references (in both main text and supplementary materials)	-
Please ensure that journal name abbreviations match those found in the National Center for Biotechnology Information (NCBI) databases (http://www.ncbi.nlm.nih.gov/nlmcatalog/journals), and are appropriately formatted and capitalised. Where website addresses are cited, please include the complete URL and specify the date of access (e.g. [accessed: 12/06/2023]).	Journal name abbreviations match NCBI databases.<br>Citations for websites include complete URLs and date of access.	-
Please also see https://journals.plos.org/plosmedicine/s/submission-guidelines#loc-references for further details on reference formatting.	References have been formatted as per the PLOS website.	-
OBSERVATIONAL STUDIES		
Abstract: Please include the study design, population and setting, number of participants, years during which the study took place (enrollment and follow up), length of follow up, and main outcome measures.	The study design, population and setting, number of participants, study years, length of follow up, and main outcome measures have been reported in the Abstract.	Lines 68-91, pages 3-4
Please ensure that the study is reported according to the STROBE (or appropriate STOBE extension) guideline (available from: https://www.equator-network.org/reporting-guidelines/strobe) and include the completed STROBE (or STROBE extension)	See below – this study has been reported according to the Reporting of studies Conducted using Observational Routinely collected health Data for Pharmaco-Epidemiology (RECORD-PE) statement. The	-

Editorial request	Author response	Page / line number
checklist as Supporting Information. Please add the following statement, or similar, to the Methods: "This study is reported as per the Strengthening the Reporting of Observational Studies in Epidemiology (STROBE) guideline (S1 Checklist)." When completing the checklist, please use section and paragraph numbers, rather than page numbers.	corresponding checklist is included as S1 Checklist.	-
[FOR POPULATION HEALTH/REGISTRY STUDIES] Please ensure that the study is reported according to the RECORD guideline (available from https://www.record-statement.org) and include the completed checklist as Supporting Information. Please add the following statement, or similar, to the Methods: "This study is reported as per the Reporting of Studies Conducted using Observational Routinely-Collected Data (RECORD) guideline (S1 Checklist)." When completing the checklist, please use section and paragraph numbers, rather than page numbers.	The following sentence is included in the Methods section: "*This study is reported in accordance with the Reporting of studies Conducted using Observational Routinely collected health Data for Pharmaco-Epidemiology (RECORD-PE) statement (S1 Checklist)*."	Lines 169-171, page 7
[FOR POPULATION HEALTH ESTIMATES] Please ensure that the study is reported according to the GATHER statement (available from https://www.equator-network.org/reporting-guidelines/gather-statement) and include the completed checklist as Supporting Information. Please add the following statement, or similar, to the Methods: "This study is reported as per the Guidelines for Accurate and Transparent Health Estimates Reporting (GATHER) statement (S1 Checklist)." When completing the checklist, please use section and paragraph numbers, rather than page numbers.	N/A	-

**Editorial request**	**Author response**	**Page / line number**
For all observational studies, in the manuscript text, please indicate: (1) the specific hypotheses you intended to test, (2) the analytical methods by which you planned to test them, (3) the analyses you actually performed, and (4) when reported analyses differ from those that were planned, transparent explanations for differences that affect the reliability of the study's results. If a reported analysis was performed based on an interesting but unanticipated pattern in the data, please be clear that the analysis was data driven.	(1) The specific hypothesis tested is outlined in the Introduction.<br>(2)-(3) Planned and executed analytical methods are outlined in the Methods section<br>(4) No post-hoc analyses were conducted	Lines 164-165, page 7<br>Lines 167-332, pages 7-14
Please state in the Methods section whether the study had a prospective protocol or analysis plan. If a prospective analysis plan (from your funding proposal, IRB or other ethics committee submission, study protocol, or other planning document written before analyzing the data) was used in designing the study, please include the relevant document(s) with your revised manuscript as a Supporting Information file to be published alongside your study and cite it in the Methods section. A legend for this file should be included at the end of your manuscript. If no such document exists, please make sure that the Methods section transparently describes when analyses were planned, and when/why any data-driven changes to analyses took place. Changes in the analysis, including those made in response to peer review comments, should be identified as such in the Methods section of the paper, with rationale.	No prospective protocol / analysis plan was published. However, all planned analyses are outlined in detail in the Methods section.	Lines 167-332, pages 7-14

Technical requests

**Technical request**	**Author response**	**Page / line number**
1) We ask that a manuscript source file is provided at Revision. Please upload your manuscript file as a .doc, .docx, .rtf or .tex.	Manuscript file has been uploaded in .docx format	-
2) Please provide separate figure files in .tif or .eps format.<br><br>For more information about figure files please see our guidelines:<br><br>https://journals.plos.org/plosmedicine/s/figures<br><br>https://journals.plos.org/plosmedicine/s/figures#loc-file-requirements	Figures have been provided in .tif format	-
3) We have noticed that you have uploaded Supporting Information files, but you have not included a list of legends. Please add a full list of legends for your Supporting Information files after the references list.	Captions for supporting information files have been added at the end of the manuscript document.	Lines 781-831, pages 38-39

REFERENCES

1. Koch FC, Olivier J, Brett J, Buckley NA, Gisev N, Pearson S, et al. The impact of tightened prescribing restrictions for PBS-subsidised opioid medicines and the introduction of half-pack sizes, Australia, 2020-21: an interrupted time series analysis. Med J Aust. 2024;220(6):315-22. Epub 20240324. doi: 10.5694/mja2.52257. PubMed PMID: 38522006.
2. Paleczny S, Osagie N, Sethi J, Cusimano D. Validity and reliability International Classification of Diseases-10 codes for all forms of injury: A systematic review. PLoS One. 2024;19(2):e0298411. Epub 20240229. doi: 10.1371/journal.pone.0298411. PubMed PMID: 38421992; PubMed Central PMCID: PMCPMC10903801.
3. Hopkins RE, Bharat C, Buizen L, Close J, Ivers R, Draper B, et al. Age-Related Risk of Serious Fall Events and Opioid Analgesic Use. JAMA Intern Med. 2024;184(4):394-401. doi: 10.1001/jamainternmed.2023.8154. PubMed PMID: 38373005; PubMed Central PMCID: PMCPMC10877504.
4. Zoega H, Falster MO, Gillies MB, Litchfield M, Camacho X, Bruno C, et al. The Medicines Intelligence Data Platform: A Population-Based Data Resource From New South Wales, Australia. Pharmacoepidemiol Drug Saf. 2024;33(8):e5887. doi: 10.1002/pds.5887. PubMed PMID: 39145404.
