# Supplementary material for: Short-term risk of falls among initiators of controlled-release tapentadol versus oxycodone in New South Wales between 2014 and 2020: A population-based retrospective cohort study
Source: PLoS Med. 2026 Aug 11;23(8):e1004950. doi: 10.1371/journal.pmed.1004950 (PMC13460682; doi:10.1371/journal.pmed.1004950)
Supplement: Supplementary file 28 [file pmed.1004950.s030.pdf]

14 June 2026
Dr Andreia Cunha
Senior Editor, PLOS Medicine

Dear Dr Cunha,

Re: Author response to reviewer and editorial comments on manuscript #PMEDICINE-D-26-00438R2 "Short-term risk of falls among initiators of controlled-release tapentadol versus oxycodone: A population-based cohort study"

Thank you for the opportunity to respond to reviewer and editorial comments. We are grateful for the insightful feedback. We have carefully considered the comments and revised the manuscript accordingly.

Please find responses to the editorial and technical requests and reviewer comments outlined in the tables below. Our revised manuscript is enclosed. Please note that the page numbers in our responses relate to those in the clean version of the manuscript.

Kind regards,
Ximena Camacho (on behalf of the authors)

**Requests from Editors**	**Author response**	**Page / line number**
GENERAL EDITORIAL REQUESTS		
Please confirm that your title complies with PLOS Medicine's style. Your title must be nondeclarative and not a question. It should begin with main concept if possible. "Effect of" should be used only if causality can be inferred, i.e., for an RCT. Please place the study design ("A randomized controlled trial," "A retrospective study," "A modelling study," etc.) in the subtitle (ie, after a colon).	The title conforms to the PLOS Medicine style. The study design ("population-based cohort study") has been stated after a colon.	Page 1, lines 1-2
Please confirm that your abstract complies with our requirements, including format (three sections: Background, Methods and Findings, and Conclusions) and providing all the information relevant to this study type https://journals.plos.org/plosmedicine/s/submission-guidelines#loc-abstract	The abstract conforms with the journal requirements.	-
In the abstract, please include the important dependent variables that are adjusted for in the analyses.	We have specified that a propensity score matched approach has been applied to adjust for baseline confounding.	Page 3, line 73
In the author summary, in the final bullet point of 'What Do These Findings Mean?', please include the main limitations of the study in non-technical language.	The main limitations of the study are outlined in the final bullet point of the 'What Do These Findings Mean?' heading of the Author Summary.	Page 5, lines 132-135
Please ensure that the Introduction ends with a clear description of the study question or hypothesis.	The Introduction ends with explicit mention of the study aims and clear hypothesis statement.	Page 7, lines 162-167
Please ensure that all abbreviations are defined at first use throughout the text.	Abbreviations have been defined at first use.	-
Please confirm that all numbers presented in the abstract are present and identical to numbers presented in the main manuscript text.	All numbers presented in the abstract are identical to those presented in the main text and tables.	-
GENERAL		
Please disclose any use of AI tools and technologies to the study or article content at the end of the Methods. Please specify the name(s) and version(s) of any tool(s)	No AI tools or technologies were used in the conduct of the study nor contributed to the contents of the manuscript.	Page 32, lines 558-559

Requests from Editors	Author response	Page / line number
used and a description of their use, including how the AI-assisted or generated content was evaluated by authors. Please also include a statement specifying who retains responsibility for the final manuscript. Please see our guidelines for more information: https://journals.plos.org/plosmedicine/s/ethical-publishing-practice#loc-artificial-intelligence-tools-and-technologies.	We have added the following text to the 'Contributor information' section of the postscript:<br>"*XC retains responsibility for the final manuscript.*"	
Please review your text for any use of statistical terms (such as trend or significant) and check these are supported data, and if not, please remove them.	We confirm that our use of the statistical term 'significant' is supported by the data.	Page 28, line 439
Please remove the 'conclusions' subheading from the discussion. Please also remove any other subheadings from the discussion.	We have removed all subheadings from the Discussion.	Pages 28-31
Statistical reporting: Please revise throughout the manuscript, including tables and figures.<br>- Please report statistical information as follows to improve clarity for the reader (95% CI [13,28]; p</=).<br>- Please separate upper and lower bounds with commas instead of hyphens as the latter can be confused with reporting of negative values.<br>- Please repeat statistical definitions (HR, CI etc.) for each set of parentheses.	We have reported statistical information with 95% CIs in the format provided but have not included p-values as they do not add additional information to the more informative 95% CIs.<br>Commas have been used to separate upper and lower bounds of the confidence intervals.<br>Statistical definitions have been repeated for each set of parentheses.	Page 4, lines 84-85<br>Page 21, lines 373-375<br>Page 23, lines 385-396<br>Page 27, line 417
FUNDING STATEMENT		
The funding statement should include: specific grant numbers, initials of authors who received each award, URLs to sponsors' websites. Also, please state whether any sponsors or funders (other than the named authors) played any role in study design, data collection and analysis, the decision to publish, or preparation of the manuscript. If they had no role in the research, include this sentence: "The funders had no role in study design,	We have added the following text to the Funding statement:<br>"*The funders had no role in study design, data collection and analysis, decision to publish, or preparation of the manuscript.*"<br>Individual grant numbers (and corresponding author initials) and funder URLs have been provided.	Page 32, lines 546-547

Requests from Editors	Author response	Page / line number
data collection and analysis, decision to publish, or preparation of the manuscript."		
It appears that one or more study authors are affiliated with one or more of the agencies that funded the study. Thus, the statement "The funders had no role in study design, data collection and analysis, decision to publish, or preparation of the manuscript" does not apply. Please revise the Financial Disclosure accordingly, as in "[Author name] is [author's role] at [funding agency]. The funders had no other role in study design….."	We apologise for the confusion. The affiliation "NHMRC Centre of Research Excellence in Medicines Intelligence" reflects grant funding from the NHMRC; the centre is not otherwise affiliated with the funding agency. We have therefore removed mention of the NHMRC from the affiliation on the manuscript title page and submission portal, such that the affiliation is now as follows: "Centre of Research Excellence in Medicines Intelligence, Sydney, New South Wales, Australia"	Pages 1-2, lines 6-47
COMPETING INTERESTS STATEMENT		
All authors must declare their relevant competing interests per the PLOS policy, which can be seen here: https://journals.plos.org/plosmedicine/s/competing-interests For authors with ties to industry, please indicate whether any of the interests has a financial stake in the results of the current study.	The following text has been added to the competing interests statement:<br>"*Neither entity has a financial stake in the results of the current study.*"	Page 32, lines 552-553
DATA AVAILABILITY		
The Data Availability Statement (DAS) requires revision. For the data that are freely or publicly available, in a public repository please include the DOI or accession number.	The data used to generate the results presented in this study resides with a third party; direct access is not permitted without the express permission of the approving data custodians and human research ethics committees. However, we note that the text in the Data Availability Statement may have been misleading. We have therefore amended the DAS as follows:<br>"*This research was conducted using data provided by the Australian Government Department of Health and Aged Care and the NSW Ministry of Health. Direct access to the data and analytical files to other individuals or authorities is not permitted without the express permission of the approving human research ethics committees and data custodians. Descriptions of the data underlying the results presented in the*	Page 33, lines 565-566

Requests from Editors	Author response	Page / line number
	study and information on processes for access are available via the Medicines Intelligence Data Platform website (https://www.unsw.edu.au/medicine-health/our-schools/population-health/research/centre-research-excellence-medicines-intelligence/medintel-data-platform) or European Medicines Agency Real Data Catalogue (https://catalogues.ema.europa.eu/node/1134/administrative-details). For inquiries please contact the Data Manager, Medicines Intelligence Research Program, Melisa Litchfield, at m.litchfield@unsw.edu.au. The code used in the analysis is available from Github (https://github.com/Ximena-Camacho/Tapentadol_and_falls) and archived in Zenodo (https://doi.org/10.5281/zenodo.19626255).”	
ETHICS AND CONSENT		
Please indicate the form of consent obtained (written/oral) or the reason that consent was not obtained (e.g. the data were analyzed anonymously). Please ensure that the research complies with the PLOS policy in full: https://journals.plos.org/plosmedicine/s/human-subjects-research#loc-patient-privacy-and-informed-consent-for-publication	The following text has been added to the Methods: “*This study was approved via a waiver of informed consent as the data were anonymised.*”	Page 14, lines 335-336
FIGURES		
Please ensure all figures and tables (including those in Supporting Information files) have titles and legends.	We confirm that all figures and tables have titles and legends.	-
Please define all acronyms used in each figure or table in its corresponding legend.	All acronyms have been defined in the corresponding legends for each table and figure.	-
When a p value is given, please specify the statistical test used to determine it in the legend.	No p-values are included in the figures or tables.	-
Please consider avoiding the use of red and green in order to make your figure more accessible	The main figure is black and white.<br>We have also removed the use of green in S1 Figure.	S1 Figure

Requests from Editors	Author response	Page / line number
Please provide the unadjusted comparisons as well as the adjusted comparisons in all relevant Tables	For ease of readability of the main tables, we have included unadjusted comparisons in the Supporting Information (S7 Table). We have added the following text to the Results to clarify this for the reader:<br>"*Unadjusted relative risks are presented in S7 Table.*"	Page 21, lines 376-377
Please specify the variables controlled for in all relevant Tables	Results of adjusted analyses are presented in Table 4 and Table 5; adjustment variables have been specified in the table footnotes.	Table 4 (page 24)<br>Table 5 (page 25)
OBSERVATIONAL, COHORT, CROSS-SECTIONAL, AND CASE CONTROL STUDIES		
Please ensure that the study is reported according to the RECORD guideline (available from https://www.record-statement.org) and include the completed checklist as Supporting Information. Please add the following statement, or similar, to the Methods: "This study is reported as per the Reporting of Studies Conducted using Observational Routinely-Collected Data (RECORD) guideline (S1 Checklist)." When completing the checklist, please use section and paragraph numbers, rather than page numbers	We have amended the text in the Methods to refer to the RECORD guideline as follows:<br>"*This study is reported in accordance with the Reporting of studies Conducted using Observational Routinely-collected health Data (RECORD) statement[reference 15] (S1 Checklist).*" and have updated the reference (#15 in the manuscript) accordingly.<br><br>We have updated S1 Checklist to present the completed RECORD checklist and updated the caption accordingly.	Page 7, lines 171-173<br>Page 38, lines 786-787<br><br>S1 Checklist
Did your study have a prospective protocol or analysis plan? Please state this (either way) early in the Methods section.	No prospective protocol / analysis plan was published. We have added the following text to the Methods to reflect this:<br>"*We did not publish a prospective analysis plan for this study.*"	Page 7, line 173
a) If a prospective analysis plan (from your funding proposal, IRB or other ethics committee submission, study protocol, or other planning document written before analyzing the data) was used in designing the study, please include the relevant prospectively written document with your revised manuscript as a Supporting Information file to be published alongside your study,	N/A	-

Requests from Editors	Author response	Page / line number
and cite it in the Methods section. A legend for this file should be included at the end of your manuscript.		
b) If no such document exists, please make sure that the Methods section transparently describes when analyses were planned, and when/why any data-driven changes to analyses took place.	The Methods section outlines in detail all planned analyses. There were no data-driven changes to the planned analyses.	
c) In either case, changes in the analysis-- including those made in response to peer review comments-- should be identified as such in the Methods section of the paper, with rationale."	We did not make any changes to the analysis (including during the peer-review process).	
Your study is observational and therefore causality cannot be inferred. Please remove language that implies causality and refer to associations instead.	We have amended the language throughout the manuscript to reflect the observational nature of the study.	Page 21, lines 372-373<br>Page 27, line 418<br>Page 27, line 429<br>Page 29, line 467
For all observational studies, in the manuscript text, please indicate: (1) the specific hypotheses you intended to test, (2) the analytical methods by which you planned to test them, (3) the analyses you actually performed, and (4) when reported analyses differ from those that were planned, transparent explanations for differences that affect the reliability of the study's results. If a reported analysis was performed based on an interesting but unanticipated pattern in the data, please be clear that the analysis was data-driven.	(1) The specific hypothesis tested is outlined in the Introduction.<br>(2)-(3) Planned and executed analytical methods are outlined in the Methods section.<br>(4) No post-hoc analyses were conducted.	Page 7, lines 166-167<br>Pages 7-13, lines 169-330

**Technical requests**	**Author response**	**Page / line number**
Journal Requirements		
1) Thank you for stating "project received ethical approval from the NSW Population and Health Services Research Ethics Committee (PHSREC; approval number 2020/ETH02273) and the Australian Institute of Health and Welfare Human Research Ethics Committee (AIHW HREC; approval number EO2021/1/1233)."<br><br>Please state the full name of NSW	We have amended the text in the ethics statement in the Methods as follows:<br>"*This project received ethical approval from the New South Wales Population and Health Services Research Ethics Committee (PHSREC; approval number 2020/ETH02273) and the Australian Institute of Health and Welfare Human Research Ethics Committee (AIHW HREC; approval number EO2021/1/1233).*"	Page 14, line 332

**Reviewer Comments**	**Author response**	**Page / line number**
**Reviewer #1**		
Thank you for Table S19 and additions to the text in reply to my comments. I think Table S19 is a useful addition. Thank you, also, for the comments to the other reviewers.<br>Congratulations on the good work.	Thank you for the positive feedback.	-
**Reviewer #2**		
Thank you for addressing my previous comments.<br>I suggest you use the term "injurious falls" throughout as that is what you have been studying, and not falls.	Thank you for this suggestion. We assessed falls that resulted in contact with emergency department (ED) or hospital services (or death), but we have not explicitly restricted our study population to falls that resulted in injury. Although presence of an injury is probably a valid assumption for hospital admissions (and deaths) following a fall, it may be less likely to be true for ED presentations. We agree with the reviewer that injurious falls are important to assess; however, this requires a different outcome definition that incorporates multiple codes indicating both the occurrence of a fall as well as presence of injury / trauma, and is beyond the scope of this study. We therefore feel	-

Reviewer Comments	Author response	Page / line number
	it would not be appropriate to use the suggested terminology and have left the manuscript text unchanged in this regard.	
**Reviewer #3**		
I have no further comments - the authors have responded to and addressed all the reviewer comments adequately.	Thank you for the positive feedback.	-
**Reviewer #4**		
Thank-you for the responses and clarifications to the comments provided. No further changes requested.	Thank you for the positive feedback.	-
