# Supplementary material for: Short-term risk of falls among initiators of controlled-release tapentadol versus oxycodone in New South Wales between 2014 and 2020: A population-based retrospective cohort study
Source: PLoS Med. 2026 Aug 11;23(8):e1004950. doi: 10.1371/journal.pmed.1004950 (PMC13460682; doi:10.1371/journal.pmed.1004950)
Supplement: Peer review history

# Editor report: Decision Letter 0

**DOI:** 10.1371/journal.pmed.1004950.r001

**Authors:** Andreia Cunha

6 Feb 2026

Dear Dr Camacho,

Thank you for submitting your manuscript entitled "Short-term risk of falls among initiators of controlled-release tapentadol versus oxycodone: A population-based cohort study" for consideration by PLOS Medicine.

Your manuscript has now been evaluated by the PLOS Medicine editorial staff, and I am writing to let you know that we would like to send your submission out for external peer review.

However, before we can send your manuscript to reviewers, we need you to complete your submission by providing the metadata that is required for full assessment. To this end, please login to Editorial Manager where you will find the paper in the 'Submissions Needing Revisions' folder on your homepage. Please click 'Revise Submission' from the Action Links and complete all additional questions in the submission questionnaire.

For clinical studies, please upload a copy of your trial study protocol as a supporting information file. The study protocol should be the version submitted for approval to the institutional review board or ethics committee, should include any amendments to the study protocol, as well as the date of their approval by the institutional review or ethics committee. Please also detail any deviations from the study protocol in the Methods section of your manuscript. The editors will consider the protocol and study conduct prior to a final decision for external review.

Please re-submit your manuscript within two working days, i.e. by Feb 10 2026 11:59PM.

Login to Editorial Manager here: https://www.editorialmanager.com/pmedicine

Once your full submission is complete, your paper will undergo a series of checks in preparation for peer review. Once your manuscript has passed all checks it will be sent out for review.

Feel free to email us at plosmedicine@plos.org if you have any queries relating to your submission.

Kind regards,

Andreia Cunha, PhD

Senior editor

PLOS Medicine

# Peer review: Decision Letter 1

**DOI:** 10.1371/journal.pmed.1004950.r002

**Authors:** Andreia Cunha

3 Apr 2026

Dear Dr Camacho,

I am writing on behalf of Dr. Cunha. Many thanks for submitting your manuscript "Short-term risk of falls among initiators of controlled-release tapentadol versus oxycodone: A population-based cohort study" (PMEDICINE-D-26-00438R1) to PLOS Medicine. The paper has been reviewed by subject experts and a statistician; their comments are included below and can also be accessed here: [LINK]

As you will see, the reviewers find the work interesting and have made numerous suggestions to improve the clarity of the analyses and results, as well as their implications. After discussing the paper with the editorial team and an academic editor with relevant expertise, I'm pleased to invite you to revise the paper in response to all the reviewers' comments. We plan to send the revised paper to some or all of the original reviewers, and we cannot provide any guarantees at this stage regarding publication.

In addition to these revisions, you may need to complete some formatting changes, which you will receive in a follow up email. A member of our team will be in touch with a set of requests shortly. If you do not receive a separate email within a few days, please assume that checks have been completed, and no additional changes are required.

When you upload your revision, please include a point-by-point response that addresses all of the reviewer and editorial points, indicating the changes made in the manuscript and either an excerpt of the revised text or the location (eg: page and line number) where each change can be found. Please also be sure to check the general editorial comments at the end of this letter and include these in your point-by-point response. When you resubmit your paper, please include a clean version of the paper as the main article file and a version with changes tracked as a marked-up manuscript. It may also be helpful to check the guidelines for revised papers at http://journals.plos.org/plosmedicine/s/revising-your-manuscript for any that apply to your paper.

We ask that you submit your revision by Apr 24 2026 11:59PM. However, if this deadline is not feasible, please contact me by email, and we can discuss a suitable alternative.

Don't hesitate to contact me directly with any questions (acunha@plos.org).

Best regards,

Alison

Alison Farrell, PhD

Senior editor

PLOS Medicine

afarrell@plos.org

-----------------------------------------------------------

Comments from the editors:

* Please review your manuscript and edit to ensure compliance with our inclusive language requirements https://journals.plos.org/plosmedicine/s/human-subjects-research#loc-categorization

Comments from reviewers:

Reviewer #1: Main feedback

The primary outcome is a composite of falls associated with ED visit, hospitalisation, or death. The problem with composite outcomes is that they implicitly attach equal importance to each constituent outcome. This is rarely the case. Here, falls associated with death are clearly more significant than falls associated with hospital visits. As presented, it is not possible to gauge the effect in each of the sub-outcomes. Win ratios are a way of combining multiple outcomes in a hierarchy to infer overall clinical superiority whilst respecting that outcomes are ordered by importance. I feel a win ratio analysis would have been superior to a composite outcome analysis. I am not proposing that the authors should re-analyse all their data. I do think at the very least, however, that the authors should report the event rates and relative risk for each of the three constituent components in the composite separately in the main analysis population. I.e. what is the relative risk of falls associated with death; hospitalisation; and ED presentation? It is certainly not required that each of these comparisons would be significant (although I suspect some might be). I would expect these three effects to point in the same direction, I.e. tapentadol providing benefit. This could be an analysis for the supplement.

Table 1 is very informative, very thorough, and shows how well matching has performed. Would it be fair of me to suggest that inverse probability weighting would tend to be preferred over propensity score matching these days? IPW would have had the benefit of using all the patients, rather than the matchable subset. However, I suspect that since the sample size of the presented analysis is so large, and the matching so comprehensive, that I'd expect the inferences and conclusions to be the same.

Minor feedback

In the Results section, you say there was a difference in non-study opioid use. Please add a few words to say in which direction the differences were. I.e. oxycodone CR patients were more likely to also receive immediate release oxycodone but less likely to receive codeine and tramadol.

I looked for typos but could not find any!

I know this research is not about addiction but the main reason I have heard of oxycodone is the opioids addiction crisis with OxyContin in USA. Is tapentadol similarly implicated? I expected a sentence or two in the Discussion, particularly in light of the high NNT presented. In such a situation, with an otherwise benign and cheap treatment, you might treat despite the high NNT. But of course that is not the case here. The effect you want and the effect you don't want are two sides of the same coin.

Reviewer #2: Regarding PMEDICINE-D-26-00438R1

This is a very interesting paper with thorough methodology investigating a highly relevant research question, from both a clinical and public health perspective. The authors have made a tremendous attempt to showcase the robustness of their analyses and results. I have some minor suggestions for improving the manuscript.

Abstract

The abstract would benefit from additional rationale for the study.

Introduction

The order of the text in the introduction could be improved by expanding a bit on the tolerability. Like is it varying for chronic or new users and whether serious adverse effects are larger/more common in the initiation phase, and what is the pharmacokinetic rationale for any differences between the medications.

The introduction would also benefit from clarifying a more detailed description of the indication for the two medications compared and particular if there are the same indication and contraindications.

The introduction is silent about the perspective of type of falls, that is later raised as an interesting perspective in the results.

Methods

The data is from 2020, has there been any changes in the treatment guidelines since then?

Patient and public involvement

Given the research question, is there a reason for not including stakeholders from e.g. the clinicians or health care planers?

Was repeated events of falls considered?

There exclusion criteria regarding "the included individuals should have no dispensation history for the last two years" seems to not align with the criteria that the included individuals only had to live in NWS for the year prior to initiation. If not living in NWS for the whole 2 year period, how can you ascertain the dispensation history?

A graphical presentation of the different time periods would aid readers to follow the different periods of interest for exposure and outcomes.

Are there any previous studies on the internal validity of the outcome, falls, based on the Medintel database?

It is not clear to me what is meant by "we included fall-related diagnosis codes in any position", what is position referring to? And is there a code for falls occurring in the hospital setting, how was that ascertained?

Please provide references for the covariates.

What was the rationale for the different lengths of reference for the non-study opioids (90 days), FRIDs (30 days) and no other dispensations in the year (365 days)?

Was there any information on the type of injuries sustained due to the fall? And for those with a previous surgery (in the last 30 days), any information on type of surgical procedure, reason for the surgery or length of stay in hospital?

Results

Clearly written and easy to follow. I only have a suggestion, to place the information on the matching to the method section.

For the residual confounding it would be interesting to read what type of residual confounding that worries the authors.

The negative control approach is a very good idea, however, the statistical power around the cataract surgeries are small in several instances. Why was cataract chosen, please provide some more short information about the rationale as cataract are normally performed under very strict pre-surgical conditions?

I am a bit uncertain of the "Patient and Public Involvement" section, to me it is quite narrow to confine in the feed-back from one end-user, that is also included in the project team, a colleague and author of the paper. There is however no indication on attempting to include stakeholders from the health care system planers or clinicians which, given the research question would be equally important. The section I do not think strengthen the paper and the statements are more for dissemination of findings from a project, not for this specific scientific manuscript.

Reviewer #3: This is an interesting paper that adds to the falls literature, particularly in relation to how opioid use influences an individual's risk of falling. The paper is well written, and the analysis undertaken is appropriate. I only some minor comments for consideration:

*Page 3: Some additional background information about the link between falls and opioid use would be helpful to frame the study context.

*Page 3: Avoid using the term 'suffer' as it reinforces stigma and is deficit-framing. Consider using the term 'experienced' instead.

*Page 5: Consider making it clearer as part of the research aim / objective that the analysis included examining the risks of falls in individuals who were on non-study opioids.

*Page 5: Avoid using ageist language e.g. 'elderly'. The term 'older adults' or 'older people' are preferred.

*Page 5: Given that several subgroup and sensitivity analyses were undertaken (which are appropriate), it may be helpful to state these as explicit research aims or objectives in the introduction.

*Page 9: The way in which the history of falls variable was determined as a confounder may require some explanation as the timeframe of 2 years to 60 days is slightly unusual. This may explain the relatively low proportion of individuals with a previous history of falls (Table 1) which differs markedly to the known prevalence of falls in the general community-dwelling middle-aged (18-25%) and older adult populations (30-35%).

*Page 12: Providing a little more detail about the study cohort may help readers judge whether the sample is representative of the broader population using opioids.

*Page 16: While the authors acknowledge the study's limitations, it may also be important to note that some variables may be subject to misclassification or incomplete capture (e.g., history of falls).

Reviewer #4: This manuscript is a carefully written report of an interrogation of a large dataset that has examined the research question.

There are many strengths to the approach, including use of propensity score matching involving multiple variables that may plausibly have an impact on the outcome, a large sample size, and a relatively firm composite outcome for falls based on fall related emergency department presentations, hospitalisation or death due to falls. The limitations of the approach mainly centre on what data were available for inclusion in the analyses for both the outcome (falls that did not result in hospital presentation or death but may otherwise have had a substantial impact on the life of the individual were not recorded) and predictor variables available to be used in propensity score matching. An important issue not discussed is when the decision regarding the period of data extraction (Sept 2014 to Dec 2020) was selected relative to when the authors had access to the data. The ability to select time periods of data collection after having viewed the data can increase the risk of Type 1 errors (see Journal of Physiotherapy. 2023 Oct 1;69(4):278-82). Also, an explanation of why data was not collected after Dec 2020 is also warranted given it is now 2026.

The results of the analysis indicated a large NNT (in excess of 500) to prevent one fall due in part to the infrequency of the outcome being measured. The implications of this (should Tapentadol (SR) be prescribed in preference to Oxycodone?) would benefit from more explicit discussion and analysis, indeed an economic evaluation from the government (as social health care insurer in Australia) perspective based on the evidence presented in this study would really elevate this manuscript.

Any attachments provided with reviews can be seen via the following link: [LINK]

--------------------------------------------------------- ---

General editorial requests:

(Note: not all will apply to your paper, but please check each item carefully)

*We ask every co-author listed on the manuscript to fill in a contributing author statement, making sure to declare all competing interests. If any of the co-authors have not filled in the statement, we will remind them to do so when the paper is revised. If all statements are not completed in a timely fashion this could hold up the re-review process. If new competing interests are declared later in the revision process, this may also hold up the submission. Should there be a problem getting one of your co-authors to fill in a statement we will be in contact. Please do not add or remove authors without first discussing this with the handling editor. You can see our competing interests policy here: http://journals.plos.org/plosmedicine/s/competing-interests.

*Please upload any figures associated with your paper as individual TIF or EPS files with 300dpi resolution at resubmission; please read our figure guidelines for more information on our requirements: http://journals.plos.org/plosmedicine/s/figures. While revising your submission, we strongly recommend that you use PLOS's NAAS tool (https://ngplosjournals.pagemajik.ai/artanalysis) to test your figure files. NAAS can convert your figure files to the TIFF file type and meet basic requirements (such as print size, resolution), or provide you with a report on issues that do not meet our requirements and that NAAS cannot fix.

After uploading your figures to PLOS's NAAS tool - https://ngplosjournals.pagemajik.ai/artanalysis, NAAS will process the files provided and display the results in the "Uploaded Files" section of the page as the processing is complete.

If the uploaded figures meet our requirements (or NAAS is able to fix the files to meet our requirements), the figure will be marked as "fixed" above. If NAAS is unable to fix the files, a red "failed" label will appear above.

When NAAS has confirmed that the figure files meet our requirements, please download the file via the download option, and include these NAAS processed figure files when submitting your revised manuscript.

*Please ensure that the paper adheres to the PLOS Data Availability Policy (see http://journals.plos.org/plosmedicine/s/data-availability), which requires that all data underlying the study's findings be provided in a repository or as Supporting Information. For data residing with a third party, authors are required to provide instructions with contact information (web or email address) for obtaining the data. Please note that a study author cannot be the contact person for the data. PLOS journals do not allow statements supported by "data not shown" or "unpublished results." For such statements, authors must provide supporting data or cite public sources that include it.

* Please remove the ethics statement from the Data Availability Statement. The ethics statement should be in the Methods.

*We expect all researchers with submissions to PLOS in which author-generated code underpins the findings in the manuscript to make all author-generated code available without restrictions upon publication of the work. In cases where code is central to the manuscript, we may require the code to be made available as a condition of publication. Authors are responsible for ensuring that the code is reusable and well documented. Please make any custom code available, either as part of your data deposition or as a supplementary file. Please add a sentence to your data availability statement regarding any code used in the study, e.g. "The code used in the analysis is available from Github [URL] and archived in Zenodo [DOI link]" Please review our guidelines at https://journals.plos.org/plosmedicine/s/materials-software-and-code-sharing and ensure that your code is shared in a way that follows best practice and facilitates reproducibility and reuse. Because Github depositions can be readily changed or deleted, we encourage you to make a permanent DOI'd copy (e.g. in Zenodo) and provide the URL.

*Please ensure that all authors have completed the COI questionnaire. The disclaimers in the COI declaration and the funding statement should be set apart under the heading Disclaimer.

* Please provide a URL for each funder in the funding statement. Please ensure that the manuscript metadata matches statements in the manuscript.

* We require that you include an email address for a contact for data requests. The contact may not be an author.

* Please use Vancouver style for references.

*Please ensure that the study is reported according to the STROBE guideline and include the completed STROBE checklist as Supporting Information. When completing the checklist, please use section and paragraph numbers, rather than page numbers. Please add the following statement, or similar, to the Methods: "This study is reported as per STROBE guideline (S1 Checklist)."

* Please include any additional checklists (e.g. GATHER, RECORD), as may be necessary.

FORMATTING - GENERAL

*Abstract: Please structure your abstract using the PLOS Medicine headings (Background, Methods and Findings, Conclusions). Please combine the Methods and Findings sections into one section.

* In the last sentence of the Abstract Methods and Findings section, please describe the main limitation(s) of the study's methodology.

*At this stage, we ask that you include a short, non-technical Author Summary of your research to make findings accessible to a wide audience that includes both scientists and non-scientists. The Author Summary should immediately follow the Abstract in your revised manuscript. This text is subject to editorial change and should be distinct from the scientific abstract. Ideally each sub-heading should contain 2-3 single sentence, concise bullet points containing the most salient points from your study.

* In the final bullet point of 'What Do These Findings Mean?', please include the main limitations of the study in non-technical language. Please see our author guidelines for more information: https://journals.plos.org/plosmedicine/s/revising-your-manuscript#loc-author-summary.

*Please express the main results with 95% CIs as well as p values. When reporting p values please report as p<0.001 and where higher as the exact p value p=0.002, for example. Throughout, suggest reporting statistical information as follows to improve clarity for the reader "22% (95% CI [13%,28%]; p</=)". Please be sure to define all numerical values at first use.

*Please include page numbers and line numbers in the manuscript file. Use continuous line numbers (do not restart the numbering on each page).

*Please cite the reference numbers in square brackets. Citations should precede punctuation.

FIGURES AND TABLES

*Please provide titles and legends for all figures and tables (including those in Supporting Information files).

*Please define all abbreviations used in each figure/table (including those in Supporting Information files).

*Please consider avoiding the use of red and green in order to make your figure more accessible to those with color blindness.

SUPPLEMENTARY MATERIAL

*Please note that supplementary material will be posted as supplied by the authors. Therefore, please amend it according to the relevant comments outlined here.

*Please cite your Supporting Information as outlined here: https://journals.plos.org/plosmedicine/s/supporting-information

REFERENCES

*PLOS uses the numbered citation (citation-sequence) method and first six authors, et al.

*Please ensure that journal name abbreviations match those found in the National Center for Biotechnology Information (NCBI) databases (http://www.ncbi.nlm.nih.gov/nlmcatalog/journals), and are appropriately formatted and capitalised.

*Where website addresses are cited, please include the complete URL and specify the date of access (e.g. [accessed: 12/06/2023]).

*Please also see https://journals.plos.org/plosmedicine/s/submission-guidelines#loc-references for further details on reference formatting.

OBSERVATIONAL STUDIES

*Abstract: Please include the study design, population and setting, number of participants, years during which the study took place (enrollment and follow up), length of follow up, and main outcome measures.

*Please ensure that the study is reported according to the STROBE (or appropriate STOBE extension) guideline (available from: https://www.equator-network.org/reporting-guidelines/strobe) and include the completed STROBE (or STROBE extension) checklist as Supporting Information. Please add the following statement, or similar, to the Methods: "This study is reported as per the Strengthening the Reporting of Observational Studies in Epidemiology (STROBE) guideline (S1 Checklist)." When completing the checklist, please use section and paragraph numbers, rather than page numbers.

*[FOR POPULATION HEALTH/REGISTRY STUDIES] Please ensure that the study is reported according to the RECORD guideline (available from https://www.record-statement.org) and include the completed checklist as Supporting Information. Please add the following statement, or similar, to the Methods: "This study is reported as per the Reporting of Studies Conducted using Observational Routinely-Collected Data (RECORD) guideline (S1 Checklist)." When completing the checklist, please use section and paragraph numbers, rather than page numbers.

*[FOR POPULATION HEALTH ESTIMATES] Please ensure that the study is reported according to the GATHER statement (available from https://www.equator-network.org/reporting-guidelines/gather-statement) and include the completed checklist as Supporting Information. Please add the following statement, or similar, to the Methods: "This study is reported as per the Guidelines for Accurate and Transparent Health Estimates Reporting (GATHER) statement (S1 Checklist)." When completing the checklist, please use section and paragraph numbers, rather than page numbers.

*For all observational studies, in the manuscript text, please indicate: (1) the specific hypotheses you intended to test, (2) the analytical methods by which you planned to test them, (3) the analyses you actually performed, and (4) when reported analyses differ from those that were planned, transparent explanations for differences that affect the reliability of the study's results. If a reported analysis was performed based on an interesting but unanticipated pattern in the data, please be clear that the analysis was data driven.

*Please state in the Methods section whether the study had a prospective protocol or analysis plan. If a prospective analysis plan (from your funding proposal, IRB or other ethics committee submission, study protocol, or other planning document written before analyzing the data) was used in designing the study, please include the relevant document(s) with your revised manuscript as a Supporting Information file to be published alongside your study and cite it in the Methods section. A legend for this file should be included at the end of your manuscript. If no such document exists, please make sure that the Methods section transparently describes when analyses were planned, and when/why any data-driven changes to analyses took place. Changes in the analysis, including those made in response to peer review comments, should be identified as such in the Methods section of the paper, with rationale.

# Author comment: Author response to Decision Letter 2

**DOI:** 10.1371/journal.pmed.1004950.r003

30 Apr 2026

Attachment Submitted filename: Response_to_reviewers.pdf

# Peer review: Decision Letter 2

**DOI:** 10.1371/journal.pmed.1004950.r004

**Authors:** Andreia Cunha

8 Jun 2026

Dear Dr. Camacho,

Thank you very much for re-submitting your manuscript "Short-term risk of falls among initiators of controlled-release tapentadol versus oxycodone: A population-based cohort study" (PMEDICINE-D-26-00438R2) for review by PLOS Medicine.

I have discussed the paper with my colleagues and the academic editor and it was also seen again by the original reviewers. I am pleased to say that provided the remaining reviewer, editorial and production issues are dealt with we are planning to accept the paper for publication in the journal.

The remaining issues that need to be addressed are listed at the end of this email. Any accompanying reviewer attachments can be seen via the link below. Please take these into account before resubmitting your manuscript:

[LINK]

***Please note while forming your response, if your article is accepted, you may have the opportunity to make the peer review history publicly available. The record will include editor decision letters (with reviews) and your responses to reviewer comments. If eligible, we will contact you to opt in or out.***

In revising the manuscript for further consideration here, please ensure you address the specific points made by each reviewer and the editors. In your rebuttal letter you should indicate your response to the reviewers' and editors' comments and the changes you have made in the manuscript. Please submit a clean version of the paper as the main article file. A version with changes marked must also be uploaded as a marked up manuscript file.

Please also check the guidelines for revised papers at http://journals.plos.org/plosmedicine/s/revising-your-manuscript for any that apply to your paper. If you haven't already, we ask that you provide a short, non-technical Author Summary of your research to make findings accessible to a wide audience that includes both scientists and non-scientists. The Author Summary should immediately follow the Abstract in your revised manuscript. This text is subject to editorial change and should be distinct from the scientific abstract.

In addition to these revisions, you may need to complete some formatting changes, which you will receive in a follow up email. A member of our team will be in touch with a set of requests shortly. If you do not receive a separate email within a few days, please assume that checks have been completed, and no additional changes are required.

We expect to receive your revised manuscript within 1 week. Please email us (plosmedicine@plos.org) if you have any questions or concerns.

We ask every co-author listed on the manuscript to fill in a contributing author statement. If any of the co-authors have not filled in the statement, we will remind them to do so when the paper is revised. If all statements are not completed in a timely fashion this could hold up the re-review process. Should there be a problem getting one of your co-authors to fill in a statement we will be in contact. YOU MUST NOT ADD OR REMOVE AUTHORS UNLESS YOU HAVE ALERTED THE EDITOR HANDLING THE MANUSCRIPT TO THE CHANGE AND THEY SPECIFICALLY HAVE AGREED TO IT.

Please ensure that the paper adheres to the PLOS Data Availability Policy (see http://journals.plos.org/plosmedicine/s/data-availability), which requires that all data underlying the study's findings be provided in a repository or as Supporting Information. For data residing with a third party, authors are required to provide instructions with contact information for obtaining the data. PLOS journals do not allow statements supported by "data not shown" or "unpublished results." For such statements, authors must provide supporting data or cite public sources that include it.

To enhance the reproducibility of your results, we recommend that you deposit your laboratory protocols in protocols.io, where a protocol can be assigned its own identifier (DOI) such that it can be cited independently in the future. Additionally, PLOS ONE offers an option to publish peer-reviewed clinical study protocols. Read more information on sharing protocols at https://plos.org/protocols?utm_medium=editorial-email&utm_source=authorletters&utm_campaign=protocols

Please review your reference list to ensure that it is complete and correct. If you have cited papers that have been retracted, please include the rationale for doing so in the manuscript text, or remove these references and replace them with relevant current references. Any changes to the reference list should be mentioned in the rebuttal letter that accompanies your revised manuscript.

Please note, when your manuscript is accepted, an uncorrected proof of your manuscript will be published online ahead of the final version, unless you've already opted out via the online submission form. If, for any reason, you do not want an earlier version of your manuscript published online or are unsure if you have already indicated as such, please let the journal staff know immediately at plosmedicine@plos.org.

If you have any questions in the meantime, please contact me or the journal staff on plosmedicine@plos.org.

We look forward to receiving the revised manuscript by Jun 15 2026 11:59PM.

Sincerely,

Andreia Cunha, PhD

Senior Editor

PLOS Medicine

plosmedicine.org

------------------------------------------------------------

Requests from Editors:

GENERAL EDITORIAL REQUESTS

* Please confirm that your title complies with PLOS Medicine's style. Your title must be nondeclarative and not a question. It should begin with main concept if possible. "Effect of" should be used only if causality can be inferred, i.e., for an RCT. Please place the study design ("A randomized controlled trial," "A retrospective study," "A modelling study," etc.) in the subtitle (ie, after a colon).

* Please confirm that your abstract complies with our requirements, including format (three sections: Background, Methods and Findings, and Conclusions) and providing all the information relevant to this study type https://journals.plos.org/plosmedicine/s/submission-guidelines#loc-abstract

* In the abstract, please include the important dependent variables that are adjusted for in the analyses.

* In the author summary, in the final bullet point of 'What Do These Findings Mean?', please include the main limitations of the study in non-technical language.

* Please ensure that the Introduction ends with a clear description of the study question or hypothesis.

* Please ensure that all abbreviations are defined at first use throughout the text.

* Please confirm that all numbers presented in the abstract are present and identical to numbers presented in the main manuscript text.

GENERAL

* Please disclose any use of AI tools and technologies to the study or article content at the end of the Methods. Please specify the name(s) and version(s) of any tool(s) used and a description of their use, including how the AI-assisted or generated content was evaluated by authors. Please also include a statement specifying who retains responsibility for the final manuscript. Please see our guidelines for more information: https://journals.plos.org/plosmedicine/s/ethical-publishing-practice#loc-artificial-intelligence-tools-and-technologies.

* Please review your text for any use of statistical terms (such as trend or significant) and check these are supported data, and if not, please remove them.

* Please remove the 'conclusions' subheading from the discussion. Please also remove any other subheadings from the discussion.

* Statistical reporting: Please revise throughout the manuscript, including tables and figures.

- Please report statistical information as follows to improve clarity for the reader (95% CI [13,28]; p</=).

- Please separate upper and lower bounds with commas instead of hyphens as the latter can be confused with reporting of negative values.

- Please repeat statistical definitions (HR, CI etc.) for each set of parentheses.

FUNDING STATEMENT

* The funding statement should include: specific grant numbers, initials of authors who received each award, URLs to sponsors’ websites. Also, please state whether any sponsors or funders (other than the named authors) played any role in study design, data collection and analysis, the decision to publish, or preparation of the manuscript. If they had no role in the research, include this sentence: “The funders had no role in study design, data collection and analysis, decision to publish, or preparation of the manuscript.”

* It appears that one or more study authors are affiliated with one or more of the agencies that funded the study. Thus, the statement “The funders had no role in study design, data collection and analysis, decision to publish, or preparation of the manuscript” does not apply. Please revise the Financial Disclosure accordingly, as in "[Author name] is [author's role] at [funding agency]. The funders had no other role in study design…..”

COMPETING INTERESTS STATEMENT

* All authors must declare their relevant competing interests per the PLOS policy, which can be seen here: https://journals.plos.org/plosmedicine/s/competing-interests For authors with ties to industry, please indicate whether any of the interests has a financial stake in the results of the current study.

DATA AVAILABILITY

* The Data Availability Statement (DAS) requires revision. For the data that are freely or publicly available, in a public repository please include the DOI or accession number.

ETHICS AND CONSENT

* Please indicate the form of consent obtained (written/oral) or the reason that consent was not obtained (e.g. the data were analyzed anonymously). Please ensure that the research complies with the PLOS policy in full: https://journals.plos.org/plosmedicine/s/human-subjects-research#loc-patient-privacy-and-informed-consent-for-publication

FIGURES

* Please ensure all figures and tables (including those in Supporting Information files) have titles and legends.

* Please define all acronyms used in each figure or table in its corresponding legend.

* When a p value is given, please specify the statistical test used to determine it in the legend.

* Please consider avoiding the use of red and green in order to make your figure more accessible

* Please provide the unadjusted comparisons as well as the adjusted comparisons in all relevant Tables

* Please specify the variables controlled for in all relevant Tables

OBSERVATIONAL, COHORT, CROSS-SECTIONAL, AND CASE CONTROL STUDIES

* Please ensure that the study is reported according to the RECORD guideline (available from https://www.record-statement.org) and include the completed checklist as Supporting Information. Please add the following statement, or similar, to the Methods: "This study is reported as per the Reporting of Studies Conducted using Observational Routinely-Collected Data (RECORD) guideline (S1 Checklist)." When completing the checklist, please use section and paragraph numbers, rather than page numbers

* Did your study have a prospective protocol or analysis plan? Please state this (either way) early in the Methods section.

a) If a prospective analysis plan (from your funding proposal, IRB or other ethics committee submission, study protocol, or other planning document written before analyzing the data) was used in designing the study, please include the relevant prospectively written document with your revised manuscript as a Supporting Information file to be published alongside your study, and cite it in the Methods section. A legend for this file should be included at the end of your manuscript.

b) If no such document exists, please make sure that the Methods section transparently describes when analyses were planned, and when/why any data-driven changes to analyses took place.

c) In either case, changes in the analysis-- including those made in response to peer review comments-- should be identified as such in the Methods section of the paper, with rationale."

* Your study is observational and therefore causality cannot be inferred. Please remove language that implies causality and refer to associations instead.

* For all observational studies, in the manuscript text, please indicate: (1) the specific hypotheses you intended to test, (2) the analytical methods by which you planned to test them, (3) the analyses you actually performed, and (4) when reported analyses differ from those that were planned, transparent explanations for differences that affect the reliability of the study's results. If a reported analysis was performed based on an interesting but unanticipated pattern in the data, please be clear that the analysis was data-driven.

Comments from Reviewers:

Reviewer #1:

Thank you for Table S19 and additions to the text in reply to my comments. I think Table S19 is a useful addition.

Thank you, also, for the comments to the other reviewers.

Congratulations on the good work.

Reviewer #2: Thank you for addressing my previous comments.

I suggest you use the term "injurious falls" throughout as that is what you have been studying, and not falls.

Reviewer #3: I have no further comments - the authors have responded to and addressed all the reviewer comments adequately.

Reviewer #4: Thank-you for the responses and clarifications to the comments provided. No further changes requested.

Any attachments provided with reviews can be seen via the following link:

[LINK]

# Author comment: Author response to Decision Letter 3

**DOI:** 10.1371/journal.pmed.1004950.r005

28 Jun 2026

Attachment Submitted filename: Response_to_reviewers_auresp_3.pdf

# Editor report: Decision Letter 3

**DOI:** 10.1371/journal.pmed.1004950.r006

**Authors:** Andreia Cunha

29 Jun 2026

Dear Dr Camacho,

On behalf of my colleagues and the Academic Editor, Dr Rana Hinman, I am pleased to inform you that we have agreed to publish your manuscript "Short-term risk of falls among initiators of controlled-release tapentadol versus oxycodone: A population-based cohort study" (PMEDICINE-D-26-00438R3) in PLOS Medicine.

Before your manuscript can be formally accepted you will need to complete some formatting changes, namely to the title and supplementary files, which you will receive in a follow up email. Please be aware that it may take several days for you to receive this email; during this time no action is required by you. Once you have received these formatting requests, please note that your manuscript will not be scheduled for publication until you have made the required changes.

In the meantime, please log into Editorial Manager at http://www.editorialmanager.com/pmedicine/, click the "Update My Information" link at the top of the page, and update your user information to ensure an efficient production process.

PRESS

We frequently collaborate with press offices. If your institution or institutions have a press office, please notify them about your upcoming paper at this point, to enable them to help maximise its impact. If the press office is planning to promote your findings, we would be grateful if they could coordinate with medicinepress@plos.org. If you have not yet opted out of the early version process, we ask that you notify us immediately of any press plans so that we may do so on your behalf.

We also ask that you take this opportunity to read our Embargo Policy regarding the discussion, promotion and media coverage of work that is yet to be published by PLOS. As your manuscript is not yet published, it is bound by the conditions of our Embargo Policy. Please be aware that this policy is in place both to ensure that any press coverage of your article is fully substantiated and to provide a direct link between such coverage and the published work. For full details of our Embargo Policy, please visit http://www.plos.org/about/media-inquiries/embargo-policy/.

To enhance the reproducibility of your results, we recommend that you deposit your laboratory protocols in protocols.io, where a protocol can be assigned its own identifier (DOI) such that it can be cited independently in the future. Additionally, PLOS ONE offers an option to publish peer-reviewed clinical study protocols. Read more information on sharing protocols at https://plos.org/protocols?utm_medium=editorial-email&utm_source=authorletters&utm_campaign=protocols

Thank you again for submitting to PLOS Medicine. We look forward to publishing your paper.

Sincerely,

Andreia Cunha, PhD

Senior Editor

PLOS Medicine
